# Development and virtual validation of a novel digital workflow to rehabilitate palatal defects by using smartphone-integrated stereophotogrammetry (*SPINS*)

**DOI:** 10.1038/s41598-021-87240-9

**Published:** 2021-04-19

**Authors:** Taseef Hasan Farook, Nafij Bin Jamayet, Jawaad Ahmed Asif, Abdul Sattar Din, Muhammad Nasiruddin Mahyuddin, Mohammad Khursheed Alam

**Affiliations:** 1 School of Dental Sciences, Health Campus, Universiti Sains Malaysia, Kelantan, Malaysia; 2 Division of Clinical Dentistry (Prosthodontics), School of Dentistry, International Medical University, Jalan Jalil Perkasa-19, Bukit Jalil, 57000 Kuala Lumpur, Malaysia; 3 Consultant Oral and Maxillofacial Surgeon, Prince Mutaib Bin Abdul Aziz Hospital, Ministry of Health, Al-Jouf, Kingdom of Saudi Arabia; 4 School of Electrical and Electronic Engineering, Universiti Sains Malaysia, Penang, Malaysia; 5 College of Dentistry, Jouf University, Sakaka, Kingdom of Saudi Arabia

**Keywords:** Dentistry, Health care economics, Health services, Software, Biomedical engineering, Electrical and electronic engineering

## Abstract

Palatal defects are rehabilitated by fabricating maxillofacial prostheses called obturators. The treatment incorporates taking deviously unpredictable impressions to facsimile the palatal defects into plaster casts for obturator fabrication in the dental laboratory. The casts are then digitally stored using expensive hardware to prevent physical damage or data loss and, when required, future obturators are digitally designed, and 3D printed. Our objective was to construct and validate an economic in-house smartphone-integrated stereophotogrammetry (SPINS) 3D scanner and to evaluate its accuracy in designing prosthetics using open source/free (OS/F) digital pipeline. Palatal defect models were scanned using SPINS and its accuracy was compared against the standard laser scanner for virtual area and volumetric parameters. SPINS derived 3D models were then used to design obturators by using (OS/F) software. The resultant obturators were virtually compared against standard medical software designs. There were no significant differences in any of the virtual parameters when evaluating the accuracy of both SPINS, as well as OS/F derived obturators. However, limitations in the design process resulted in minimal dissimilarities. With further improvements, SPINS based prosthetic rehabilitation could create a viable, low cost method for rural and developing health services to embrace maxillofacial record keeping and digitised prosthetic rehabilitation.

## Introduction

Removable obturator prostheses are commonly provisioned to those with palatal defects, acquired or congenital. The conventional procedure of fabricating an obturator requires taking an impression of the upper dental arch and defected palate following a series of complex methods which are both technique sensitive and pose serious risks to the patient. Risks include dislodgement of impression material into the defect cavity, immunological reactions toward foreign body within a healing cavity and secondary infections necessitating hospitalisation^1,2^.

If taken successfully, the impression of the palate is converted into dental cast models, upon which a temporary prosthesis is fabricated and periodically readjusted to facilitate proper healing. Clinicians may also wish to refer to the defect cast models during readjustment phase. After provision of temporary obturators, the models are stored away and retrieved upon future needs; which is usually during fabrication of definitive prostheses after completion of healing^3^. The physical cast models are frequently damaged, deteriorated, misplaced, or weathered which warrant taking another set of impressions prior to definitive prostheses fabrication. This ordeal creates inconveniences for both the patient and the clinician, prolonging treatment durations and increase the likelihood of compromised clinical success. In recent years, CAD–CAM and rapid prototyping in prosthetic dentistry have introduced methods of averting these issues^4^.

Advanced healthcare facilities within urban vicinities have slowly transitioned toward digital record keeping and the use of proprietary 3D scanners with CAD systems for complete digital prosthetic rehabilitations. After scanning, the prosthetic moulds/templates are then virtually designed in CAD, and 3D printed on demand; averting impression-related risks to the patient while saving valuable time for the clinicians^4^. This allows for the cast models to be stored indefinitely within a virtual space averting the risks of weathering and accidental damage.

While proprietary 3D scanners with medical grade CAD systems dominate the standard pathways for digitised rehabilitation, state-of-the-art practices such as these are almost exclusively limited to urban and wealthy establishments^5^. Rehabilitation care provided at remote practices often lack the funds and necessary support to facilitate a fully digitized workflow^6^. Furthermore, majority of the registered patients requiring prosthetic rehabilitation in suburban and rural are from middle to low socio-economic demographics^7^. Proprietary Scanning and CAD technology is expensive to purchase and upkeep, the cost of which cannot be economically justified without subsidisation when digitization is attempted for patients who require financial aid within peripheral clinical practices.

Increased smartphone usage and a plethora of associated affordable technological advances have created substantial inclusivity worldwide^8^. Feasible, constantly improving, open source and portable technology is slowly bridging the gap between developing and developed countries^6^ as the world advances toward space revolution with NASA’s latest Mars exploration unit (‘Ingenuity’) also reported to have been powered by smartphone processors^9^.

Therefore, it is now appropriate to ask the following questions:

1. Can a smartphone be used as an alternative to scan defect cast models as accurately as one of the standard laser scanners?
2. Can a cost-effective digital workflow be developed which utilise the smartphone scanned models to accurately facilitate digital obturator templates?

To answer these questions, the current study attempted to construct and virtually validate an in-house, low-cost *S*mart*P*hone *IN*tegrated *S*tereophotogrammetry (*SPINS*) 3D scanner to scan simulated defect models. The data from the scans were then utilised to design obturator bulbs by using open source/free CAD software (OS/F). It was hypothesised that there will be no significant virtual differences between:

1. 3D cast models derived from SPINS versus models obtained from standard laser scanning.
2. obturator templates designed using OS/F versus the standard proprietary digital workflows.

## Material and methods

**Figure 1:**
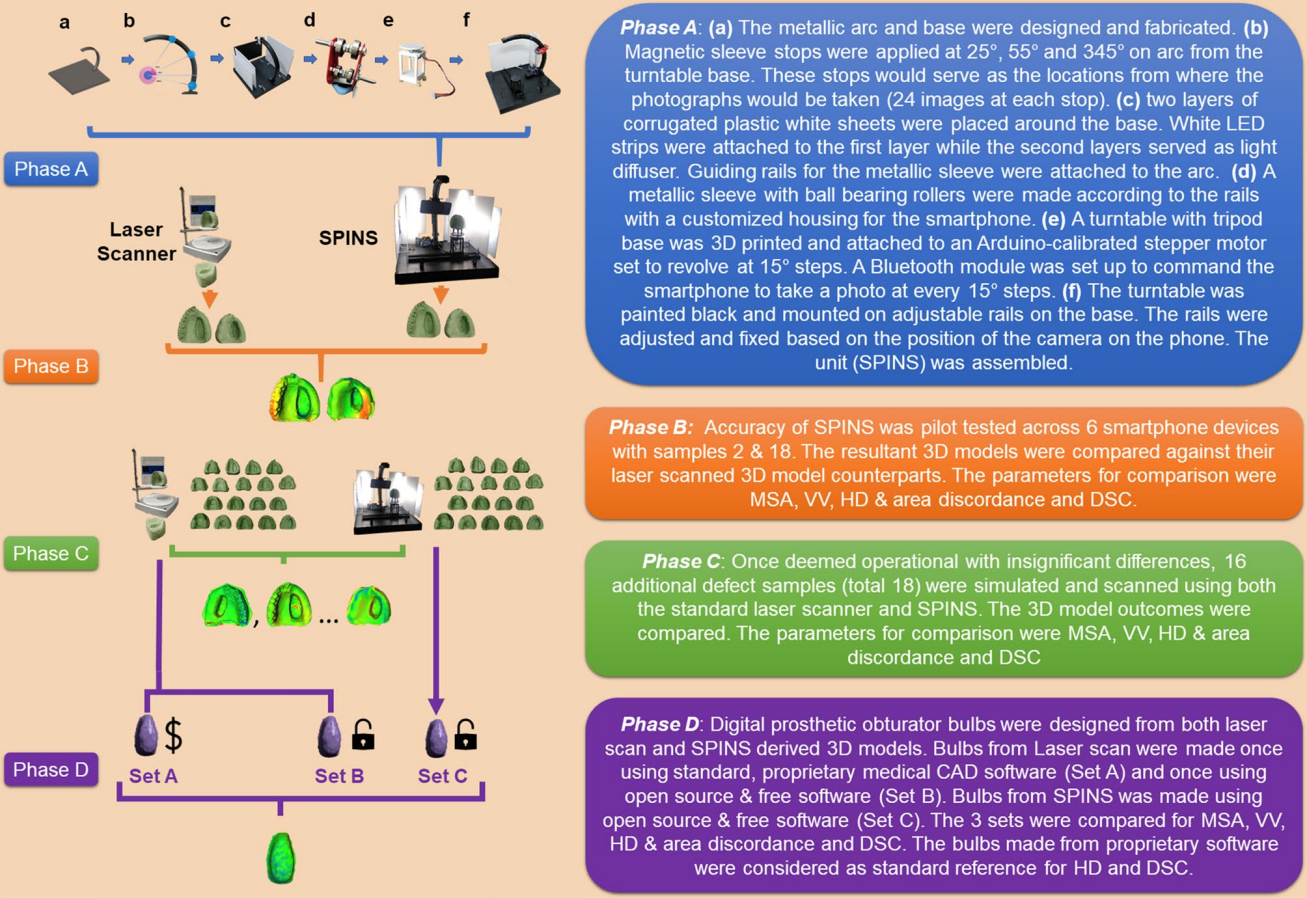
A graphical flowchart summary of the workflow followed in the study. The phases (A–D) and their respective descriptions are colour coded. *SPINS* smartphone-integrated stereophotogrammetry, *MSA* mesh surface area, *VV* virtual volume, *HD* Hausdorff’s distance, *DSC* dice similarity coefficient.

This study was conducted in four phases (A–D) which have been explained in Fig. 1. To improve readability; details of the study design, the technical specifications, operational codes, software commands and relevant data have been presented within Supplementary A & B and cited as appropriate within this text. Supplementary A is further subdivided into sections 1–5, while Supplementary B is structured according to data relevant to the phase (A–D).

SPINS development was based on the principles of photogrammetry, where multiple partially overlapping images of a static object are ‘stitched’ to produce a single 3D model. The workflow was developed and cast models were scanned following experts’ advice and recommendations obtained from previous literature^10,11^. The development process and the relevant tools have been elaborately detailed in Supplementary A, sections 1 and 2. The codes used to program the microcontroller-based turn table have been detailed in Supplementary A, section 3. All physical cast models in this study were fabricated from pre-existing silicone moulds and hence no human samples were required. The defects were designed alongside maxillofacial surgeons to ensure realistic recreation of palatal defects. A standard desktop laser scanner (NextEngine, Santa Monica) was used as control reference to validate SPINS^12^.

For Phase C, an effect size of 0.8 (Cohen’s d) with α = 0.10 and power of 0.80 suggested a total of 30 samples. A similar study^10^ determined an effect of 6.18 (G-power^13^) and therefore a large effect size was deemed appropriate to observe significant changes. The upper limit of the conventional range for α (0.01–0.10)^14^ was considered fair as avoiding Type II errors was deemed more important for validating the current hypotheses, thus requiring higher power (1 − β)^14^. To mitigate possible discrepancies, an additional 20% samples were placed in each group to create a total sample size of 36 with an actual power of 0.86.

**Figure 2:**
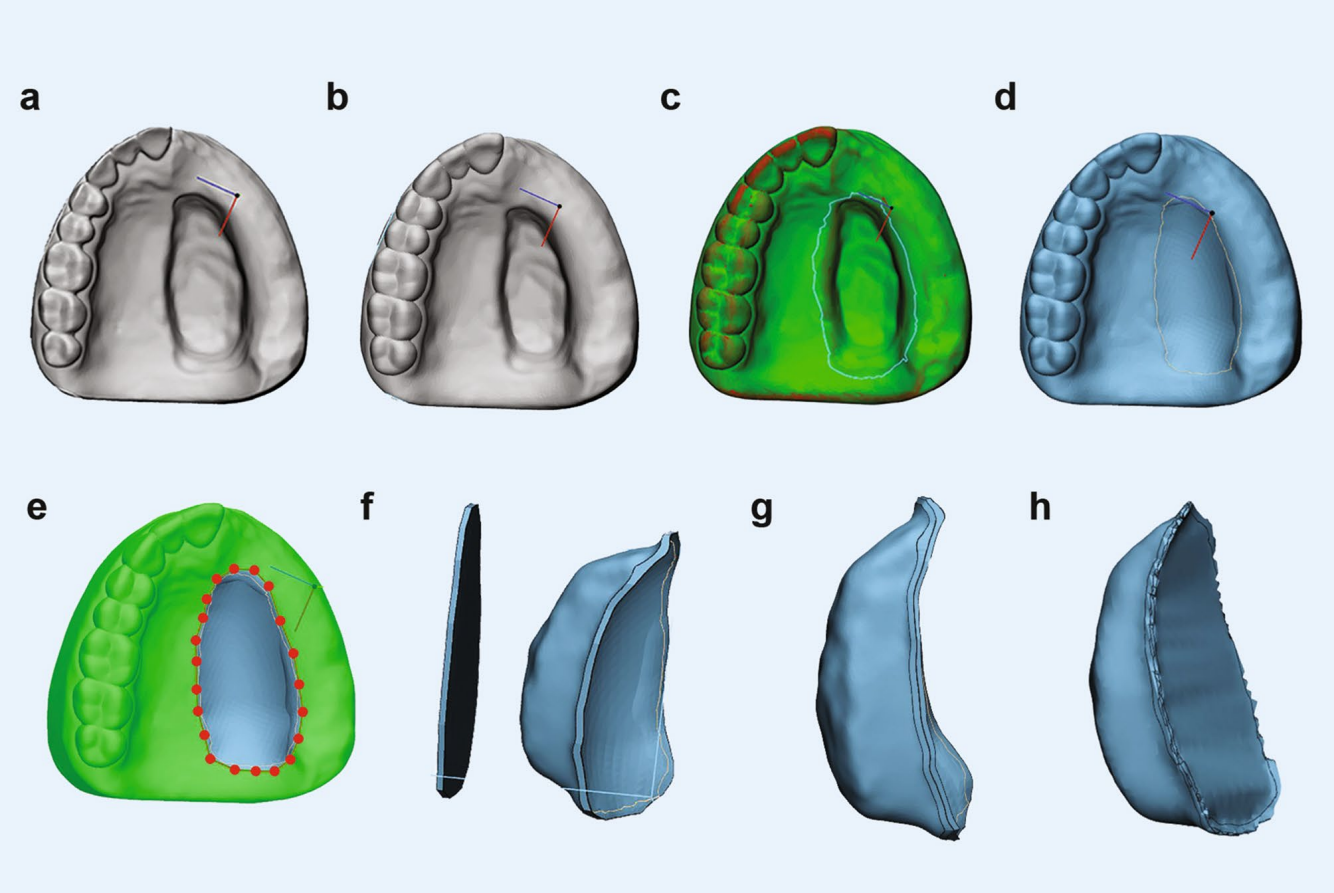
Design of bulbs using 3-matics (**a**) import STL, (**b**) make hollow, (**c**) design curve around defect, (**d**) reconstruct surface, (**e**) trim the reconstruction zone, (**f**) remove any excess mesh, (**g**) create chamfered margins, (**h**) wrap, smooth and export prosthesis.

**Figure 3:**
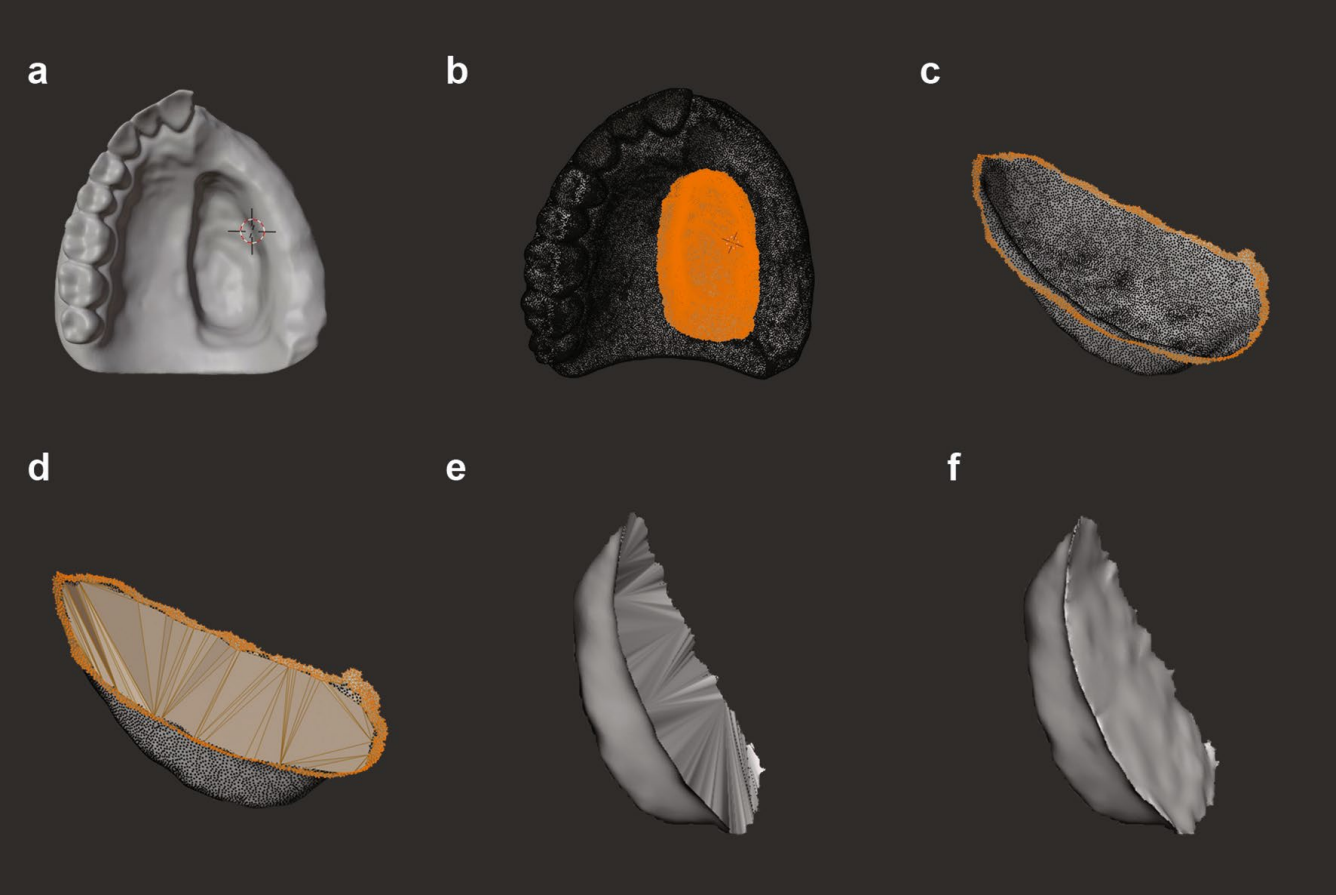
Design of bulbs using OS/F (**a**) import STL, (**b**) select the defect, (**c**) flip normal and select horizontal peripheral vertices, (**d**) fill outer surface, (**e**) analyse and auto repair to make watertight, (**f**) sculpt and export prosthesis.

For Phase D, An effect size of 0.505 was derived from a previous study^15^ with α = 0.05 and power of 0.80 (G-power^13^). Considering the possibility of human-generated errors, an additional 30% samples were considered, resulting in 54 samples and an actual power of 0.91. Digital bulbs were first designed by proprietary medical grade CAD software (3-matics, Materialise, Belgium) (Fig. 2) and then using OS/F software (Blender 2.82, Blender Foundation, Netherlands^16^; Meshmixer, AutoDesk Inc, USA^17^) (Fig. 3). Materialise software suite was chosen as control as it had been most frequently used for maxillofacial prosthetic rehabilitation^4,18^. All the software commands applied within the current study have been detailed in Supplementary A, section 4.

The virtual parameters for comparison in phases B, C and D (Fig. 1) were mesh surface area (MSA), virtual volume (VV), Hausdorff’s distance (HD) & Area discordance and Dice similarity coefficient (DSC). These values were generated by using open-source solutions; Meshlab^19^ and CloudCompare^20^ and were based on previous research^12,21,22^. The acceptability thresholds for HD was set at < 0.5mm^23^ and DSC was set at 0.70^24,25^. MSA, VV, HD & Area discordance were computer generated values while DSC was calculated by using the following formula:

$$\frac{2*(A\cap B)}{A+B}$$

where A is the VV of the standard reference and B is the VV of the comparison model. All statistical analyses in this study were performed using SPSS v.24 (IBM Corp. USA) and have been detailed within the “Results” section.

## Results

### Phases A & B

For all 6 smartphones, there were no significant differences in MSA (*P* = 0.97) and VV (*P* = 0.94) (Supplementary B, Phases A & B). In addition, an average HD of < 0.5 mm and DSC > 0.9 on two completely different sets of dentitions (Models 2 and 18) suggest that the SPINS 3D models generated from all 6 smartphones were very similar and therefore the choice of smartphone would likely have negligible influence on the comparative outcomes for Phases C and D.

### Phase C

**Figure 4:**
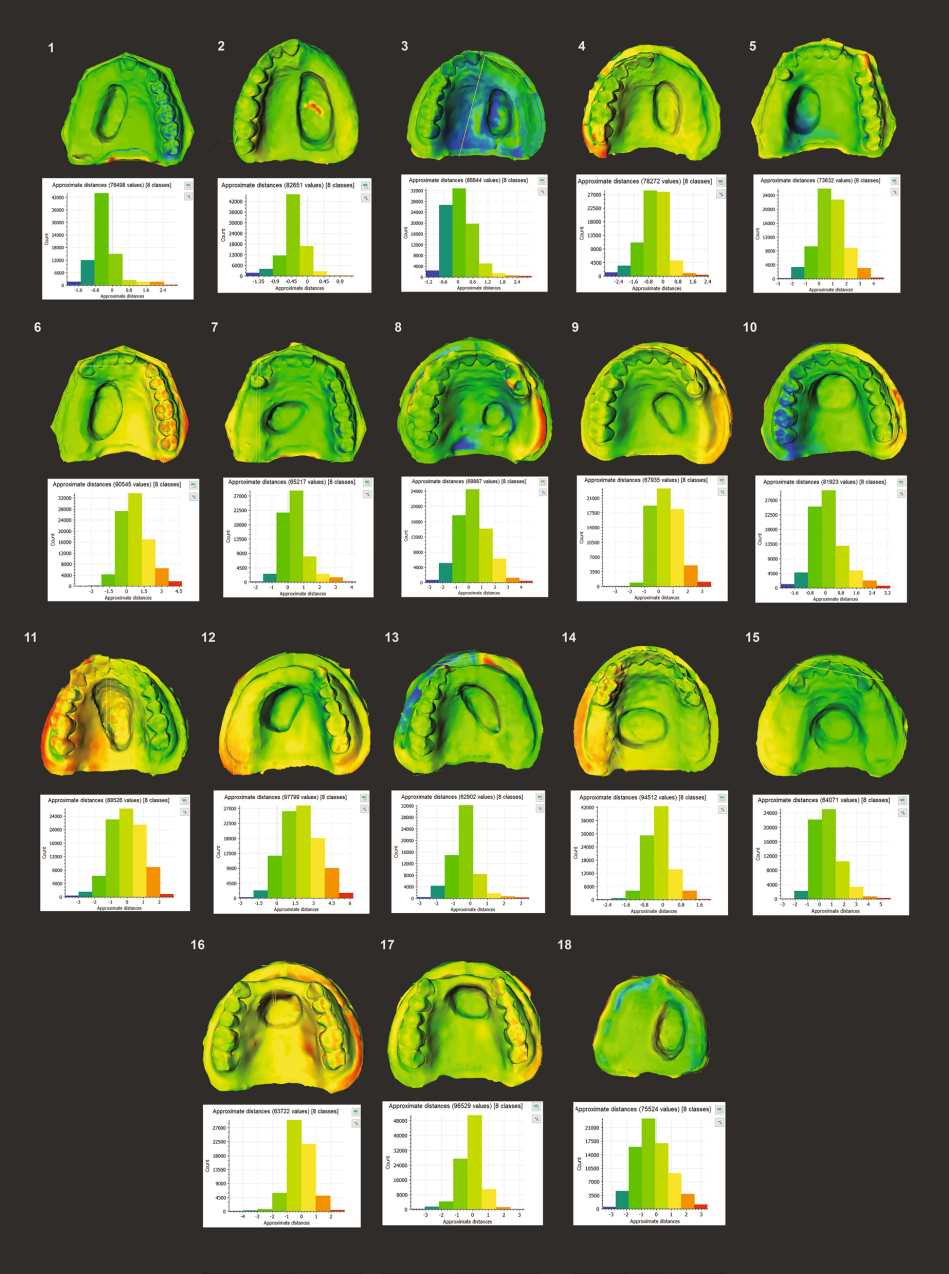
Area discordance reports for Phase C. The amount of HD variation per model comparison is shown in the bar chart and is colour coded. The exact location of the discrepancy is shown on the model above with the respective colours.

Virtual property differences between models derived from laser scan and smartphone-based photogrammetry are presented in Table 1. There were no statistically significant differences in MSA (*P* = 0.55) and VV (*P* = 0.73) across all 36 models, with HD < 0.5 mm and an average similarity of 92.97% (Supplementary B, Phase C). Individual model analysis showed that 72.22% of the models independently met HD acceptability (< 0.5 mm) while all the models met DSC acceptability (> 0.7). DSC ranged between 0.90 and 0.99. Area discordance of the models (Fig. 4) demonstrated that majority of the point discrepancies and lack of spatial overlap comprised at the margins and fine anatomy of the dental arch, but less frequently in the defect area. Laser scan reproduced more accurate dental arches than SPINS set at 72 images per 3D model.

**Table 1:** Comparison of virtual properties between SPINS and laser scanned defect models.

Mesh surface area (MSA)				
	Median	IQR	Z-stat	*P* value^a^
SPINS	14,775.18	1473.75	− 0.60	0.55
Laser scan	14,468.59	1685.61		

Virtual volume (VV)				
	Mean VV (mm^3^)	SD	t-stat (*df*)	*P* value^b^
SPINS	72,202.36	11,259.14	0.35 (34)	0.73
Laser scan	70,932.60	10,630.00		

Hausdorff’s distance				
	Mean HD (mm)			
SPINS	0.44			
Laser scan				

Spatial overlap by dice similarity coefficient (DSC)				
	Mean DSC	Percentage DSC^c^		
SPINS	0.93	92.97%		
Laser Scan				

^a^Mann–Whitney U test. *P* value set at 0.05. Parametric assumptions for MSA were not met. Curve skewed to the right; Shapiro–Wilk test was significant (*P* < 0.05). *IQR* interquartile range

^b^Independent t-test. *P* value set at 0.05. All assumptions for parametric test were met. Kolmogorov Smirnov test and Shapiro–Wilk test not significant (*P* > 0.05). Levene’s test not significant (*P* = 0.983). Data was normally distributed. *SD* standard deviation, *df* degree of freedom

^c^Laser-scanned models selected as reference for HD and DSC. Percentage DSC = DSC × 100

### Phase D

There were no significant differences (*P* > 0.05) in all 4 parameters as shown in Table 2. OS/F satisfied DSC threshold of > 0.7 with an average similarity of over 80% but with mean HD of 0.55 for Set B and 0.65 for Set C. 61.1% and 94.44% of the models in Set B independently met HD and DSC acceptability thresholds, respectively. Set C, however, demonstrated that 44.44% and 94.44% of the models met acceptability thresholds. The area discordance data for each bulb is presented in Supplementary B, Phase D which show majority of the discrepancy points were located on the outer surface of the bulbs. Set C demonstrated more discrepancies on the inner bulb surfaces than Set B.

**Table 2:** Comparison of virtual properties of obturator bulbs designed via Sets A, B and C.

Mesh surface area (MSA)				
	Mean (mm^2^)	SD	F-stat (*df*)	*P* value^a^
Set A	1516.93	405.25	0.34 (2)	0.72
Set B	1426.20	410.43		
Set C	1417.82	392.42		

Virtual volume (VV)				
	Mean (mm^3^)	SD	F-stat (*df*)	*P* value^a^
Set A	2744.47	1317.68	1.18 (2)	0.32
Set B	3331.73	1458.93		
Set C	3158.38	1444.37		

Hausdorff’s distance				
	Mean (mm)	SD	t-stat (*df*)	*P* value^b^
Set B	0.55	0.40	− 0.81 (34)	0.42
Set C	0.65	0.38		

Spatial overlap by dice similarity coefficient				
	Mean	SD	t-stat (*df*)	*P* value^b^
Set B	0.82	0.09	0.60 (34)	0.55
Set C	0.80	0.09		

^a^One-way ANOVA, 3 equal groups (total n=54). *P* value set at 0.05. Kolmogorov–Smirnov test for MSA and VV is not significant (*P* > 0.05) for all groups. Data is normally distributed.

^b^Independent t-test, *P* value set at 0.05. All parameters for normal distribution met. Kolmogorov–Smirnov and Shapiro–Wilk tests not significant (*P* > 0.05). Levene test for HD and DSC were 0.593 and 0.994, respectively. HD and DSC were obtained by taking Set A as comparison reference.

## Discussion

This study was performed to create an affordable smartphone-integrated stereophotogrammetry workflow and validate whether prosthetic obturators could be designed in CAD using such a pipeline. Based on the results obtained, both null hypotheses could not be rejected as there were no significant differences (*P* > 0.05) in all four comparative parameters: MSA, VV, HD and DSC (Tables 1, 2). SPINS is not aimed at replacing the existing conventional laser or intraoral scanners, rather to propose a cost-effective option for rural and developing sectors globally to embrace digitalisation of maxillofacial prosthetic rehabilitation.

Based on the irregular nature of the defects within the current study and the convenience of computerised analyses, it was considered more practical to measure area and 3D parameters^10^ over linear measurements that were carried out in previous studies^11,15,26,27^. Surface area and volume were used to estimate the shape and dimensions of the prostheses. Hausdorff’s distance measured the mutual proximity of any given point on two similar objects, which was then visually represented by area discordance. DSC analysed the volumetric spatial overlap between two objects. As seen in previous studies^12,21,23,25^, these two parameters can provide a reliable estimate of how similar two virtual prostheses are to one another.

Majority of the software used in this research were open source and/or free as of 2021. The development of SPINS as an open-source platform entailed various issues of hardware calibration, wireless connectivity errors, transfer latency, cloud corruption and bugs within the CAD workflow. The methods applied to solve the major issues faced are highlighted in Supplementary A, section 5 and were all resolved prior to data collection. The challenges involved, along with the steep learning curve^28^ and possible input errors are likely reasons why such workflows are seldom explored^10,21,23,29^ in maxillofacial prosthetic dentistry. However, the large community support for open-source^16^, free biomedical service initiatives^6^ and recently established reliability of Blender^28^ and Meshmixer^4,29^ in prosthetic dentistry show promise of an economically viable maxillofacial prosthetic digitisation alternative. Autodesk Recap, although not open-source, was used to evaluate the viability of cloud-based photogrammetry initiated off a personal computer. Open-source platforms like Meshroom and VisualSFM can carry out the same functionalities as Autodesk Recap if dealt by a capable machine with Computer Unified Device Architecture (CUDA)^30^.

Since the digital parameters in this study were quantified through software and without human intervention, errors in measurement were considered to be minimal^31^. Thus, the large value deviations within the samples were likely due to the different model shapes and defect sizes that were simulated. Similar variations in volume were also found in Abdullah et al.’s study of skull models^32^. Emphasising on a single defect location or size would not be an accurate clinical representation as patients present with palatal defects of varying shapes and sizes, and no two defects are the same^25^.

**Figure 5:**
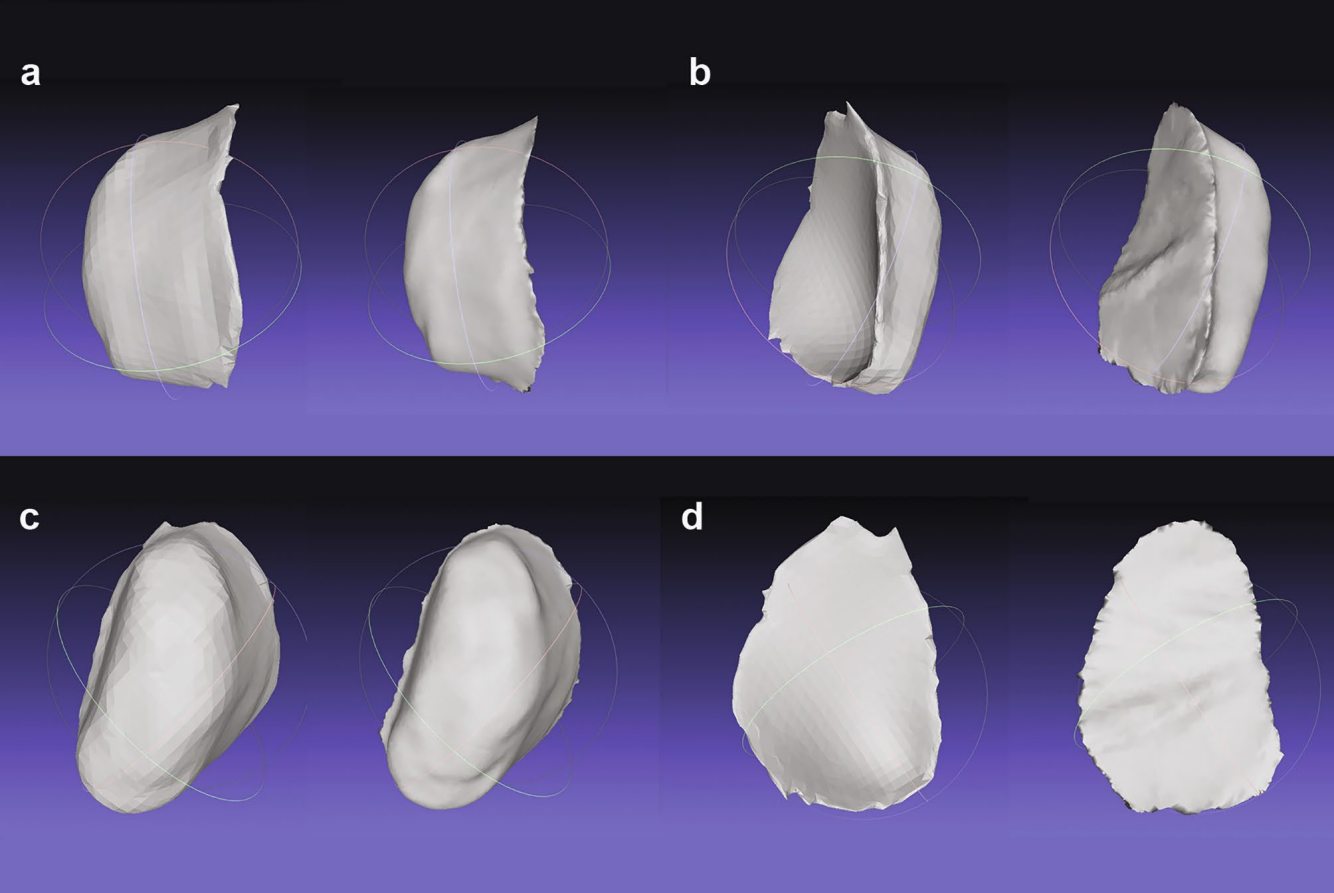
Visual comparison of proprietary and OS/F workflows on Set A (left) and Set B (right). (**a**) Right lateral surface, (**b**) left lateral surface, (**c**) inner surface, (**d**) outer surface.

The complex concaved architecture of the human palate was accurately recreated by 3-matics while constructing the prosthetic bulb. However, the authors could not reproduce that exact shape with OS/F which instead produced a flattened outer surface (Fig. 5). This flattened surface resulted in the reduction of surface area but raised the internal volume, thus creating an interpoint discrepancy when compared to 3-matics derived prostheses. Table 2 Thus, Sets B and C exceeded the mean HD acceptability threshold (0.5 mm). Although discrepancies were also found on the inner surfaces of the bulbs in Set C, these minimal differences were likely carried over from SPINS. Leon et al.^33^ explained that inadequate or unevenly distributed illumination affect mesh accuracies in 3D photogrammetry and solid-from-motion (SFM) scans which is eventually reflected on the surface of the 3D models. These minimal differences can be resolved by improving controlled illumination as well as increasing the number of images taken per cycle (> 24).

In the current study, SPINS was compared directly to a laser scanner instead of intraoral 3D scanners. intraoral scanners are handheld devices capable of recording the oral environment in real time and do not rely on generating physical cast models^34^. The technology reportedly demonstrated varying degrees of accuracy in recording dentulous and edentulous arches^35,36^. Furthermore, the art of capturing and rehabilitating palatal defects with oral scanners alone is fairly new, have caveats, and are mostly discussed through preliminary reports or case descriptions^34,37,38^. Various reports also suggest that an additional ionizing/magnetic medical scan (CT, CBCT or MRI) is required alongside intraoral scans to appropriately record the defect undercuts^12,38–40^. Aside from the obvious radiation hazards posed by these medical scans^41^, the high proprietary costs of dental intraoral scanners as well as the required investments in CT/CBCT-based imaging technology must also be considered. Howbeit, as oral scanning technology is gradually receiving wider acceptability with updated features, it will be imperative to compare the accuracy of SPINS to intraoral scanners in the near future.

While the current technique does not bypass the primary step of impression taking, it can offer a number of affordable smartphone-centric digitisation alternatives for rural service providers who may not have access to state-of-the-art facilities. When paired with a centralised cloud computing platform, remote clinics/academic centres can adopt digital maxillofacial record keeping, commence remote consultation by referring to the 3D casts, promote patient-oriented distance-learning for students and researchers of prosthetic rehabilitation and aid non-profit organisations to feasibly render digitised maxillofacial prosthetic services to cancer patients.

This research was limited to an in-vitro simulation of possible palatal defects on hard dental casts and therefore did not account for soft tissue variations. Future research recommendations for SPINS development include virtual validation against intraoral scanners, physical cast model validations and in-vivo studies of 3D printed templates and subjecting them to various clinically applicable conditions.

## Conclusion

Findings from the current in-vitro experiment suggested that open-source smartphone-based stereophotogrammetry, although not yet perfected, can be a viable, low-cost alternative to the standard laser scanner for palatal defect rehabilitation and digitisation of defect record keeping.

## Supplementary Information

Supplementary Information 1.

Supplementary Information 2.

## Data Availability

All data relevant to this study have been made available within Supplementary A and B.

## Abbreviations

3D: Three dimensional
SPINS: Smartphone-integrated stereophotogrammetry
OS/F: Open source and/or free software combination
IDE: Integrated development environment
MSA: Mesh surface area
VV: Virtual volume
HD: Hausdorff’s distance
DSC: Dice similarity coefficient
CAD: Computer-aided design
CT: Computed tomography
CBCT: Cone beamed computed tomography
MRI: Magnetic resonance imaging
CUDA: Computer unified device architecture
SFM: Solid from motion
