# Supplementary material for: Development and virtual validation of a novel digital workflow to rehabilitate palatal defects by using smartphone-integrated stereophotogrammetry (SPINS)
Source: Sci Rep. 2021 Apr 19;11:8469. doi: 10.1038/s41598-021-87240-9 (PMC8055911; doi:10.1038/s41598-021-87240-9)
Supplement: Supplementary file 1 — Supplementary Information 1. [file 41598_2021_87240_MOESM1_ESM.pdf]

SUPPLEMENTARY A: TECHNICAL NOTES FOR SPINS PALATAL REHABILITATION WORKFLOW

Table of Contents

Section 1: A Detailed Methodology 3
1.1 Phase A: Development of SPINS 3
1.2 Phase B: Preliminary test for precision of SPINS using different smartphones 4
1.3 Phase C: Test for accuracy of SPINS against standard laser scanning 5
1.4 Phase D: Validation of functionality in the design of obturator bulbs 5
Section 2: Electronic hardware used: 6
2.1 Data Capture: 6
2.2 Data processing: 6
Section 3: Arduino codes for stepper motor revolution 7
Section 4: Software operational commands 8
4.1 SPINS model fabrication by stereophotogrammetry: 8
4.1.1 AutoDesk Recap Photo 8
4.1.2 AutoDesk Meshmixer 8
4.2 Laser Scan model fabrication using NextEngine laser scanner 8
4.3 Comparison of parameters for models and designed bulb prostheses 9
4.3.1 Mesh surface area by MeshLab 9
4.3.2 Mesh Volume by Cloud Compare 9
4.3.3 Hausdorff's Distance by Cloud Compare 9
4.3.4 Dice Similarity Coefficient by Cloud Compare 9
4.4 Design of obturator bulb prostheses using 3-Matics (Materialise) 9
4.5 Calibrate 3-matics design workflow using conventional bulb parameters 10
4.6 Design of obturator bulb using open source – free software 10
4.6.1 Blender 2.82 (open source) 10
4.6.2 AutoDesk Meshmixer (free) 11
4.7 Final inspection on whether the 3D prosthetic bulb is fit for additive manufacturing 11
4.7.1 Meshlab 11
4.7.2 CloudCompare 11
4.7.3 Ultimaker Cura 4.6.1 11
Section 5: Challenges faced while designing the experiment 12

5.1 Challenges during data acquisition 12
5.1.1. Drastic changes in position of model in between one of the three cycles 12
5.1.2. Depth of field effect selected by default to focus on the model 13
5.1.3. Use of ultrawide angle (fish-eye) action cameras 14
5.1.4 Insufficient lighting on the defect 15
5.1.5. Obstruction of a surface/ reflection of un-diffused light onto a polished model surface .. 16
5.1.6. Standard focused picture and resultant model 17
5.1.7 Other issues during data acquisition 18
5.2 Challenges during CAD design 19
5.2.1 Issues with normal faces when the model contain holes/ other polygon defects (3-matics) 19
5.2.2 The need for horizontal peripheral vertices surrounding the defect (Blender – Meshmixer) 20
4.2.3 absence of horizontal peripheral vertices surrounding the defect (Blender – Meshmixer) 21
5.2.4 Incomplete selection of horizontal peripheral vertices (Blender – Meshmixer) 22
5.2.5 unconnected/excess peripheral vertices surrounding the defect (Blender – Meshmixer) . 23
5.2.6 Faces are not generated when model is imported into Meshmixer from Blender 25
5.3 Inaccuracies on SPINS model carried over to CAD design 26

Section 1: A Detailed Methodology

1.1 Phase A: Development of SPINS

The SPINS structure consisted of a turntable driven by a stepper motor, and an arc-shaped smartphone mount. The stepper motor was controlled by an Arduino UNO microcontroller board and a stepper motor driver. The Arduino board was programmed using an Arduino integrated Development Environment (IDE) software. The turntable was programmed to make a 360° turn in 24 steps, where each step was equivalent to 15° of rotation. The stepper motor used in this project had a revolution of 2048 steps per revolution in full-stepping mode. So, to make an exact 15° angle of turn, the stepper motor needed to make 85.33 steps of rotation. Since a stepper motor can only turn in an exact number of steps, the closest it could get to 15° angle of rotation was to make 85 steps of rotation, which produced 14.94°. The motor was programmed to stop for 500ms after each 85 steps (~15°), during which, the smartphone camera was wirelessly triggered by a Bluetooth shutter module to capture an image of the sample on the turntable. To trigger the image capture, the Arduino board was programmed to send a high signal for 100ms to the Bluetooth remote shutter. The cycle of 15° rotation and the image capture was repeated until the turntable made a full 360° turn, which resulted in a total of 24 images captured. Details of the hardware used, and associated Arduino codes are mentioned in later on in **Supplementary A**, **Sections 2 & 3**. The images were projected in real-time on to the user's laptop using a screen mirror tool (Airdroid, Sand Studio). Each cycle of 24 images was controlled by an Arduino switch. 24 images of the model were taken at each of the three sleeve stops (25°, 55° and 345° on the arc) while the arc position was manually switched after each 24-image capture cycle. This resulted in a total of 72 images per model after moving across all three sleeve stops.

Corrugated white plastic sheets with white 15-diode 12V LED strips acted as primary diffused light source. A diffused ring light facing perpendicularly downward onto the model was also fixed on the crest of the arc to serve as secondary light source. The luminosity at the centre of the turntable was

recorded at 1252 Lux (Lux Light Meter, Doggo Apps, Russia). A black sheet was placed in the background to prevent loss of camera focus in between shots.

Smartphones took focused images using their default smart camera systems. The images were transferred via cloud to Recap Photo (Autodesk Inc., USA); a software which automatically matched common points in each image and stitched the points to form a 3D model. The 3D models were scaled to actual size by measuring three successive linear reference distances on the physical model and entering the values for the stitched 3D model using dedicated software commands. The 3D model was then exported as STL with a maximum triangle budget of 200,000 ±10,000 triangles. The software commands have been detailed in **Supplementary A, Section 4.1**. The scans were kept in their original unoptimized format to prevent minute losses in feature and therefore the final 3D models contained more triangles (approximately 200,000 tris) as opposed to highly optimised models generated from proprietary dental scanning software (approximately 15,000 tris). Models derived from both laser scanner (NextEngine, Santa Monica, USA) and SPINS were decimated to maintain this budget and prevent an unfair mismatch during comparison.

1.2 Phase B: Preliminary test for precision of SPINS using different smartphones

Two physical models of simulated palatal defects (Model no. 2 & 18) were selected and laser scanned (NextEngine, Santa Monica) for pilot testing in phase B and software calibrations in Phase D.

6 smartphones released from 2015-2019 were chosen to pilot test SPINS. Detailed camera specification of each smartphone is mentioned in **Supplementary A, Section 2.1**. Models 2 and 18 were scanned by the smartphones and later processed by ReCap to produce 3D models. MSA, VV, HD, and DSC were analysed for all 6 smartphone results.

1.3 Phase C: Test for accuracy of SPINS against standard laser scanning

The models were digitally captured using smartphone-4 attached to SPINS (n=18) and compared against their scanned counterparts from NextEngine laser-scanner (n=18). The two sets of models were compared for MSA, VV, HD & Area Discordance and DSC.

1.4 Phase D: Validation of functionality in the design of obturator bulbs

Digital bulbs were first designed using proprietary medical grade CAD software (3-matics, Materialise, Belgium). Physical obturators for models 2 and 18 were fabricated using conventional methods by a prosthodontist, laser scanned and compared with their digitally designed counterparts. The HD and DSC acceptability thresholds were met at default settings and therefore, no calibrations were made within the software. The bulbs were designed accordingly and labelled 'Set A'.

Digital bulbs were then designed using OS/F, for both laser scan (Set B) and SPINS (Set C). The software used were Blender 2.82 (Blender Foundation, Netherlands) and Meshmixer (AutoDesk Inc, USA). The commands for both proprietary and OS/F are detailed in **Supplementary A, Sections 4.4-4.6**.

To ensure reliability of the prosthetic outputs; first, the extent of horizontal peripheral vertices (prosthetic flange) of each bulb was reviewed by two prosthodontic specialists for acceptability and progressed after both reviewers agreed on the outcome. Second, all designed bulbs were analysed using Meshlab, CloudCompare and Cura 4.6.1 (Ultimaker, Netherlands) following a simple set of software commands **(Supplementary A, Section 4.7).** The bulbs were scanned by all 3 open source platforms to ensure that they were 'watertight', error-free, and thus 3D-printable. Sets A, B and C were then evaluated for MSA, VV, HD & Area discordance and DSC.

Section 2: Electronic hardware used:

2.1 Data Capture:

1. Arduino UNO R3 (ATmega328) board with stepper motor & driver (ULN2003)
2. Bluetooth shutter printed circuit board with Bluetooth 4.0 receiver
3. Smartphone 1 (2015): 12MP, f/2.2, single camera sensor
4. Smartphone 2 (2016): 13MP, f/1.9, single camera sensor
5. Smartphone 3 (2018): 16MP, f/2.0, dual camera sensors
6. smartphone 4 (2017): 16MP, f/1.7, dual camera sensors
7. smartphone 5 (2019): 12MP, f/1.5-2.4, dual camera sensors
8. smartphone 6 (2019): 12MP, f/1.5-2.4, triple camera sensors

(All images were captured on default settings with no digital filters applied)

2.2 Data processing:

AMD Ryzen 5 2500u 15W TDP laptop (2018). 8GB DDR4 SODIMM 2400Hz RAM, 240GB m.2 NVMe SSD

Section 3: Arduino codes for stepper motor revolution

```
// Include the Arduino Stepper.h library:
#include <Stepper.h>
// Define number of steps per rotation:
const int stepsPerRevolution = 2048;
// Wiring:
// Pin 8 to IN1 on the ULN2003 driver
// Pin 9 to IN2 on the ULN2003 driver
// Pin 10 to IN3 on the ULN2003 driver
// Pin 11 to IN4 on the ULN2003 driver
// Create stepper object called 'myStepper', note the pin order:
Stepper myStepper = Stepper(stepsPerRevolution, 8, 10, 9, 11);
int n=0;

void setup() {
// Set the speed to 5 rpm:
myStepper.setSpeed(5);
pinMode(2, OUTPUT);

// Begin Serial communication at a baud rate of 9600:
Serial.begin(9600);

}
void loop() {
// Step one revolution in one direction:

if(n<24)
{
Serial.println("clockwise");
myStepper.step(85);
delay(500);

digitalWrite(2, HIGH);
delay(100);
digitalWrite(2, LOW);
delay(1500);
n++;
}

}
```

Section 4: Software operational commands

4.1 SPINS model fabrication by stereophotogrammetry:

4.1.1 AutoDesk Recap Photo

- Open Recap Photo > *object* > *add photos* > find location folder of select photos > *Ctrl + A* (select all) > *create* > name project
  [The project will upload, receive a registration number and wait in queue until ready to process]
- Download project from *my cloud drive* > *open* RCM file > *model setting* > *set scale and units* > set units to *mm* > scale by *select two-point distance* > select RD reference value > input physical value score
- Save and repeat *set scale and units* two more times (total 3 times) using another RD value (another landmark distance)
  Note: for dentulous models, use maximum mesiodistal width of any 3 teeth. For edentulous models, use height of ridge from base from 3 different points. RD value to be obtained using measuring callipers on physical models
- Edit > slice & fill > remove the tripod base of the turntable > apply
- *Export* > *Export model* > export as *STL* > target face count set to *200000* > *export* > save to directory

4.1.2 AutoDesk Meshmixer

- Import the STL into Meshmixer
- *Selec*t > highlight turntable base > *discard* (X)
- *Inspector* > *analysis* > *flat fill* > *auto-repair all* > *done*
- *Sculpt* > *shrink smooth* (str 65, size 55, depth -25) > manually smoothen turntable face > *done*
- *File* > *export as* > overwrite STL

4.2 Laser Scan model fabrication using NextEngine laser scanner

- Place model on scanner turntable > *scan* > *360 degree scan* > keep remaining settings at default > *start scan*
- Repeat scan with model repositioned at a different angulation
- *Trim* > *rectangular selector* > remove turntable and stand base
- *Align* > *place 3 data points* on central fossa of 1st molar, centre of defect, labial frenum > *fuse* > *save as* > *STL file*
- Open STL in meshmixer > *select all* > *reduce* > *triangle budget* > *preserve boundaries* > set to *200000*> *apply* > *export* > *overwrite* STL save

4.3 Comparison of parameters for models and designed bulb prostheses

4.3.1 Mesh surface area by MeshLab

Filters > *Quality Measures and Computations* > *Compute Geometric Measures* > scroll the lower right-hand box and record the value placed in 'Mesh Surface Area'

4.3.2 Mesh Volume by Cloud Compare

- Import the STL > *edit* > *normals* > *compute normal* > *per vertex*
- *Edit* > *Mesh* > *measure volumes*
  Note: the names of the STL models can be renamed in the DB tree using *F2* (*Fn + F2* in some computers). Renaming will make locating easier for steps 3 & 4

4.3.3 Hausdorff's Distance by Cloud Compare

- Import 'reference' model (Laser scanned) and compute normal
- Import 'aligned/compared' model (SPINS) model and compute normal
- Select both STL models in the DB tree using Ctrl + Left mouse button > *tools* > *registration* > *match bounding-box centers*
- *Tools* > *registration* > *fine registration ICP* > select reference and aligned models > apply and wait for 100% to complete
  (note: if bounding boxes are not symmetrical, use *translate/rotate* to roughly align both the models manually to overlap and then repeat fine registration ICP)
- With both STLs selected in the DB tree > *tools* > *distance* > *cloud/mesh distance* > select *compute* on the distance computation window > record the mean distance (HD) from the 'console bar' on the bottom of the screen (do not close the window) > scroll sideways on the distance computation window and select *approximate distances* > select histogram image > select export histogram as image
  Note: positive and negative inclinations were disregarded as the objective was to observe the amount of discrepancy, not the direction of it

4.3.4 Dice Similarity Coefficient by Cloud Compare

- Should follow immediately after Hausdorff's distance in the same operation instance
- Measure volume for both Laser scanned models and SPINS model individually and record
- Select *Plug-in* > *cork* > select *A ∩ B Boolean Operation* > wait for process to complete > select the newly generated 'intersect STL' in the DB tree and measure Mesh *volume*
- Calculate DSC using the formula DSC = 2 X volume Intersect STL / (volume of Laser scanned STL + volume of SPINS STL)

4.4 Design of obturator bulb prostheses using 3-Matics (Materialise)

- Open 3-matics > *file* > *Import part* > select model > *import STL* > select 'mm' in *scale coefficient* > select '1.00' in *user scale coefficient* > select *ok*
- *Fix* > *auto fix* > select 'model' as *entity* > *apply*
- *Design* > *hollow* > select *hollow type* as 'outside' > *apply*

- *Analyze* > Create Curvature Analysis > apply > *Curve* > *create curve* > plot curve points along the margin of defect according to the highspots and undercuts highlighted by curvature analysis > *design* > *surface construction* > select 'curve' as *entity* > *apply*
  (Note: If the curve is inadequately drawn, *edit curve* can be used to readjust.)
- *Finish* > *trim* > plot trimming points around the reconstructed defect > select 'remove outer' in *trimming method* > select 'model' as *entity* > apply
- Repeat *trim* to remove any extra mesh parts outside of reconstructed defect
  (Note: ensure that trim is done outside the limits of the reconstruction curve, otherwise face defects like inSection 4.2.1) will be observed.)
- *Finish* > *Chamfer edges* > select palatal border 'contour' > *apply*
- *Design* > *Wrap* > select model entity > *Apply*
- *Finish* > *Local Smoothening* > manually round the remaining sharp edges
- *File* > *Export* > *STL* > select model 'wrapped' as *entity* > select preferred *output directory* > *apply*
  (Note: run all the commands on default setting unless validation, 3.5 demands a change in certain parameters.)

4.5 Calibrate 3-matics design workflow using conventional bulb parameters

- Scan conventionally fabricated bulb using NextEngine laser scanner following 3.2.
- Compare volumetric parameters of conventional bulb against sample 2 to determine interpoint discrepancies (3.3.3) and spatial overlap (3.3.4).
  (Note: acceptability set at [HD < 0.5 mm] and [DSC > 0.90]. In this study, no modifications of the default parameters were required to meet acceptability threshold)

4.6 Design of obturator bulb using open source – free software

4.6.1 Blender 2.82 (open source)

- *File* > *import* > *stl* > select file from the directory > *import STL*
  (Note: If model is out of editing axis, use *rotate* and *move* within the *object mode* to manually bring it onto plane)
- *Modelling* > *select* > *none*
- *Modelling* > *select* > use *circle select* tool to highlight the defect using the left mouse button > *mesh* > *separate* > *selection*
  (Note: middle mouse button can be used to remove any excess highlights. Ensure that all undercuts of the defect are selected by revolving the model at various angles. If after separation, a hole is detected, use *Ctrl + Z* shortcut to undo the separation and correct the issue)
- Remove the original model from 'scene collection' db tree by applying right mouse click followed by *delete*
- Left click on the separated bulb from the 'scene collection' db tree > *edit mode* > *modelling* > *select* > *all*
- *Modelling* > *Mesh* > *normals* > *flip*
- *Select* > *circle select* > select the margins of the defect only (horizontal peripheral vertices) > *face* > *fill*

- Select any excess peripheral vertices and delete by *select* and *mesh* > *delete* > *vertices*
- File > export > stl > rename mesh and save

4.6.2 AutoDesk Meshmixer (free)

- File > import > load saved file
- *select* > roughly select the excess regions (outside of red ring margins) > *discard (X)*
- *Analysis* > *inspector* > *minimal fill* > *Auto Repair All*
  (Note: select the excess peripheral horizontal vertices with the red ring as reference. High accuracy is not needed as the analysis tool will auto adjust and fix the mesh accordingly)
- *Sculpt* > *robust smooth* > manually smoothen palatal face to obtain an even contour> *done*
- *File* > *export* > choose destination > *save*

4.7 Final inspection on whether the 3D prosthetic bulb is fit for additive manufacturing

4.7.1 Meshlab

Filters > *Quality Measures and Computations* > *Compute Geometric Measures* > scroll the lower right hand box and observe for volume output.

If the mesh is not 'watertight'/ has non-manifold edges/ not 3D printable, the following output will be given instead of the volume: *'Mesh is not 'watertight', no information on volume, barycenter and inertia tensor.'*

4.7.2 CloudCompare

Edit > *Mesh* > *measure volumes*

If the mesh is not 3D printable, the following output will be given instead of volume: '*the mesh has holes'*

4.7.3 Ultimaker Cura 4.6.1

- Open Cura > *marketplace* > select 'mesh tools' > agree and *install*
- Open STL file > select model > extension > mesh tools > check models

If the model is not 3D printable, the following message will be displayed: *'.stl is not watertight and may not print properly'*

Section 5: Challenges faced while designing the experiment

5.1 Challenges during data acquisition

5.1.1. Drastic changes in position of model in between one of the three cycles

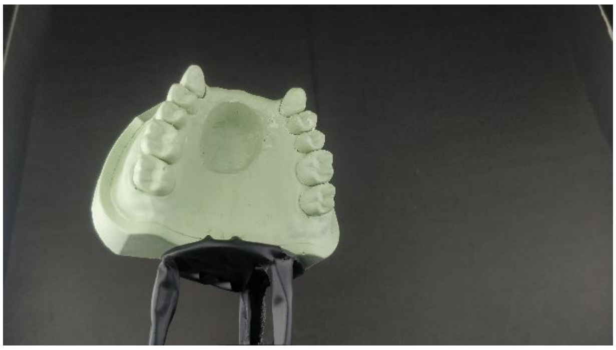

Figure 1: Sample Image of the capture cycle

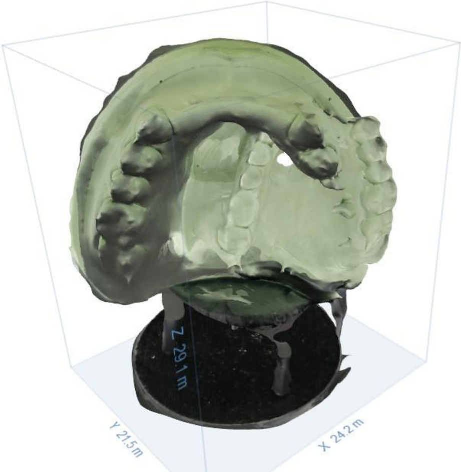

Figure 2: The distorted 3D model upon model movement

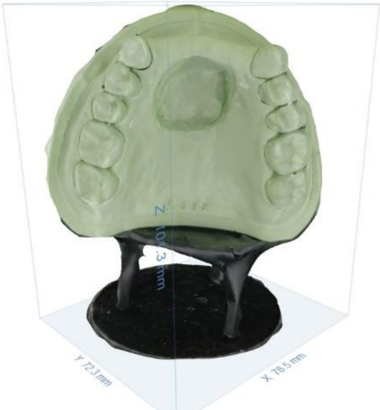

Figure 3: Results after repeating the scan while ensuring no movement to model in between cycles

5.1.2. Depth of field effect selected by default to focus on the model

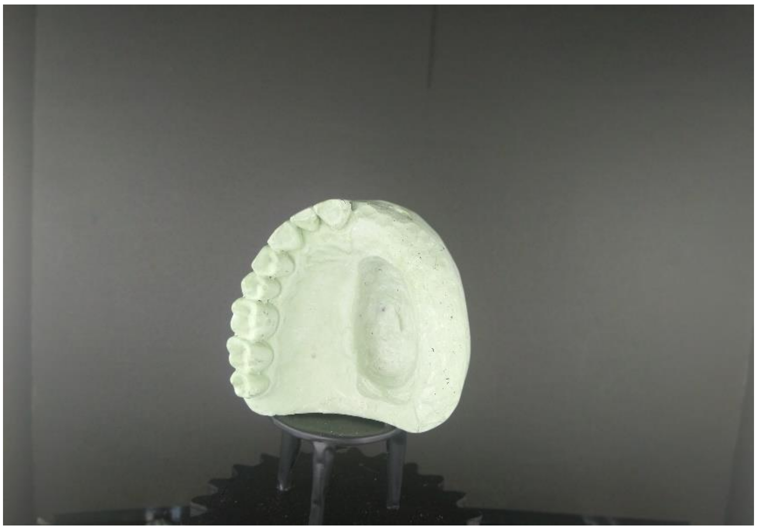

Figure 1: Example of image after setting depth of field effect as default

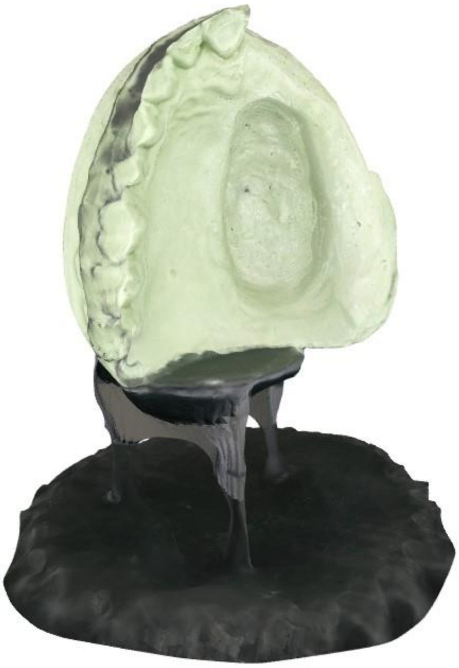

Figure 2: distortion of minute details present on the dental arch due to blurring effect

5.1.3. Use of ultrawide angle (fish-eye) action cameras

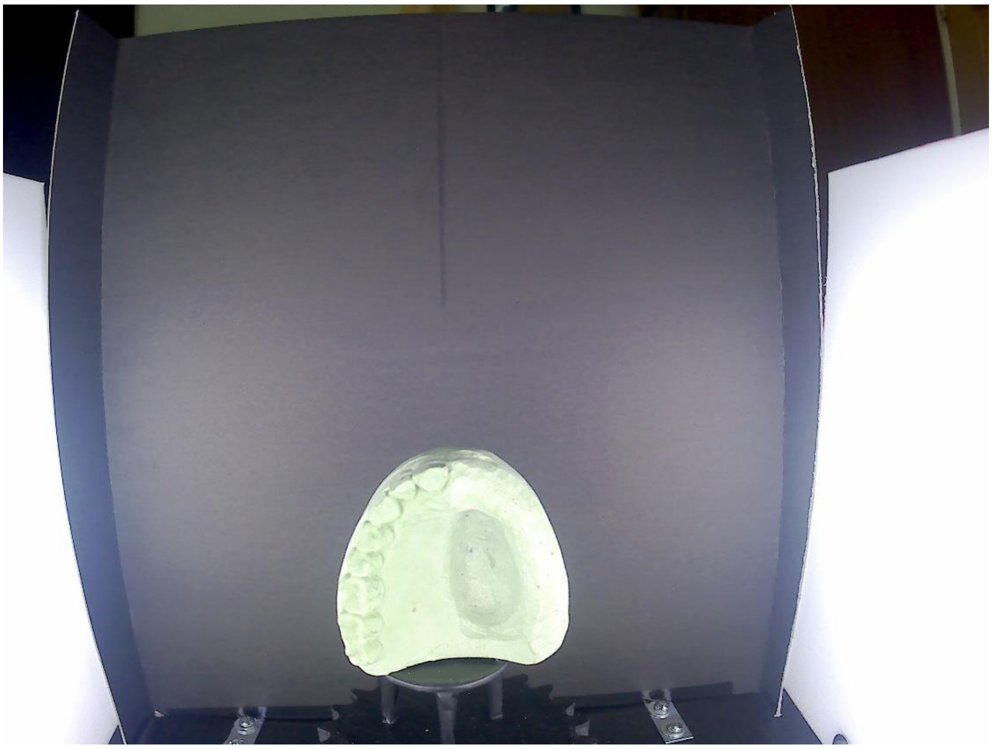

Figure 1: Sample of an ultra-wide angle shot

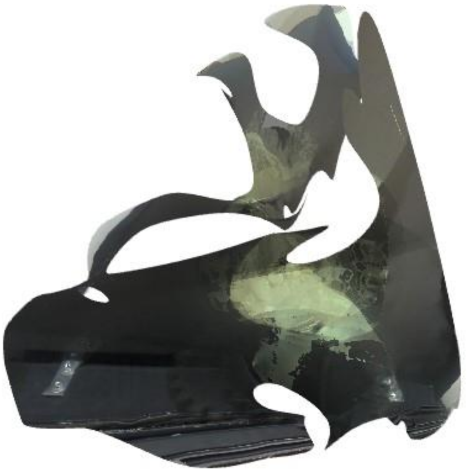

Figure 2: Resultant distortion. This was the result of the software trying to stitch multiple background stationary points which were repeatedly present in all images.

5.1.4 Insufficient lighting on the defect

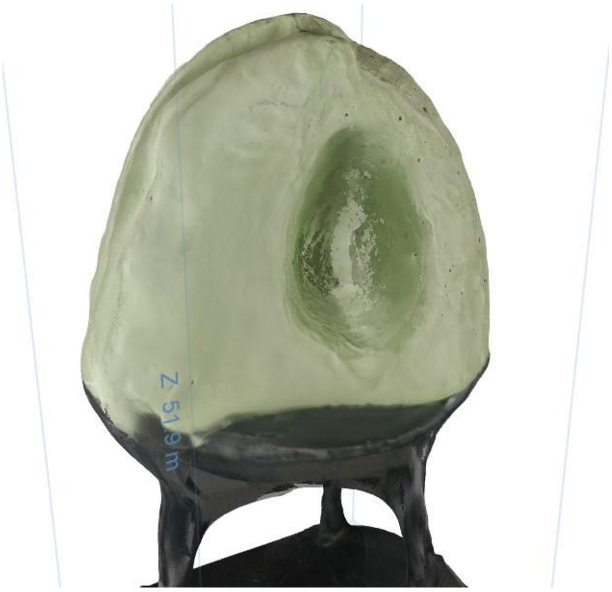

Figure 1: The 3D model resulting from insufficient lighting

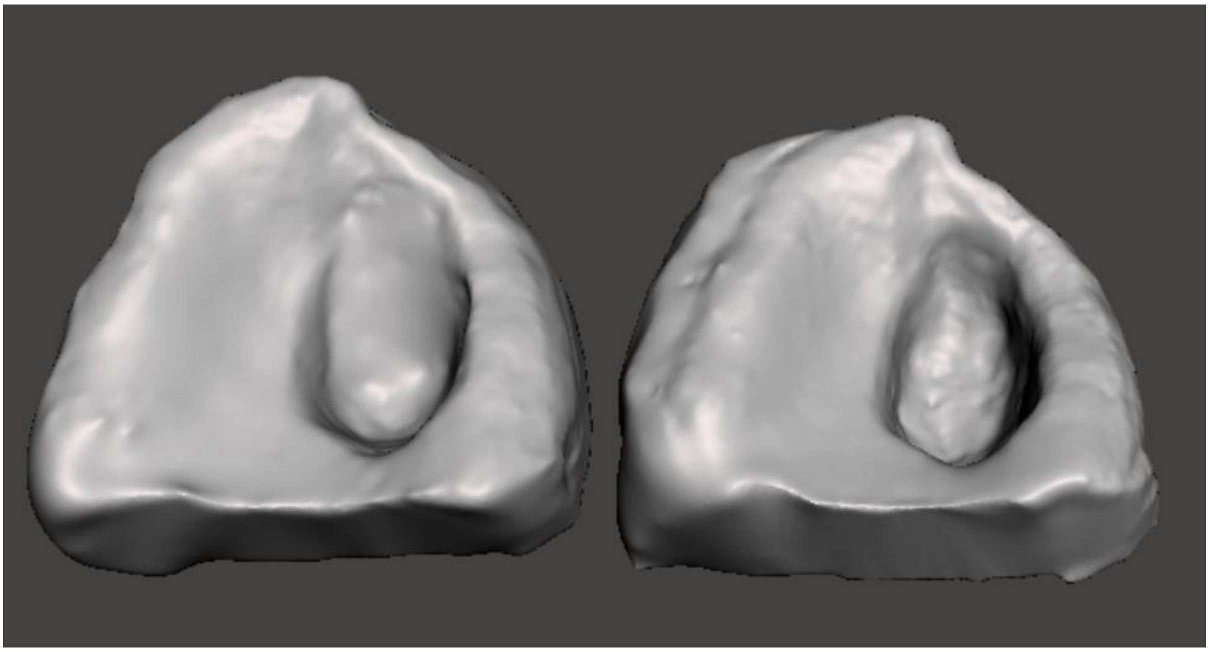

Figure 2: Comparison of the effect of insufficient lighting (left) vs adequate lighting (right) on the defect

5.1.5. Obstruction of a surface/ reflection of un-diffused light onto a polished model surface

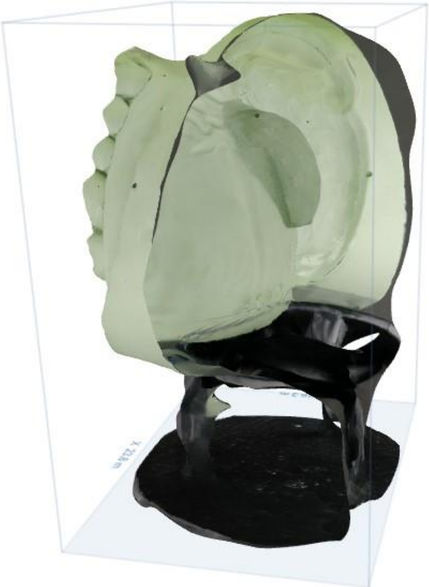

Figure 1: Inability to reconstruct the posterior base of the dental model

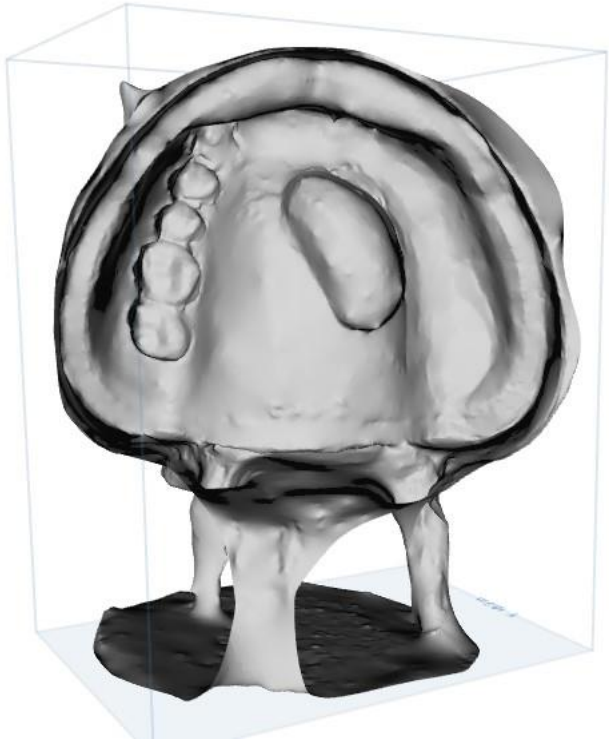

Figure 2: Although the posterior face is missing, the remainder of the dental model was reproduced accurately

5.1.6. Standard focused picture and resultant model

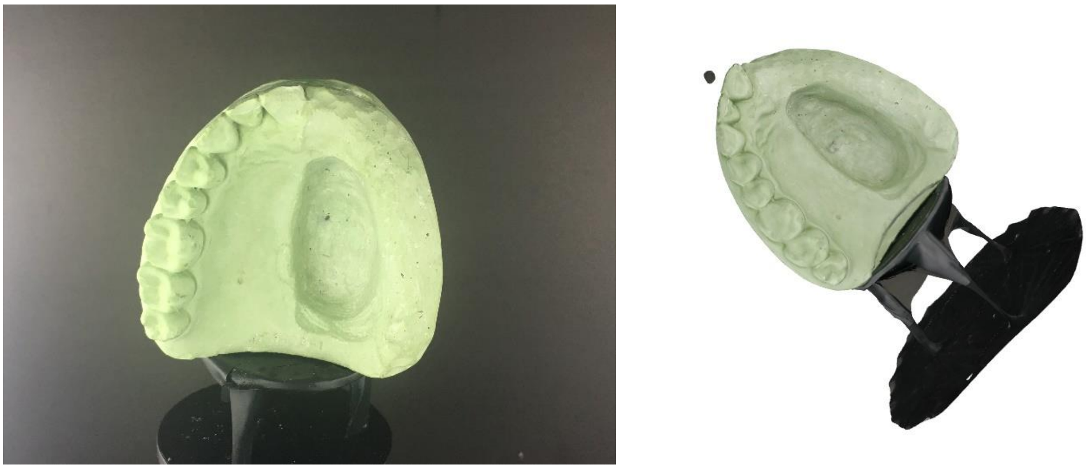

Figure 1: Example of an ideal image with resultant 3D model (Sample 2)

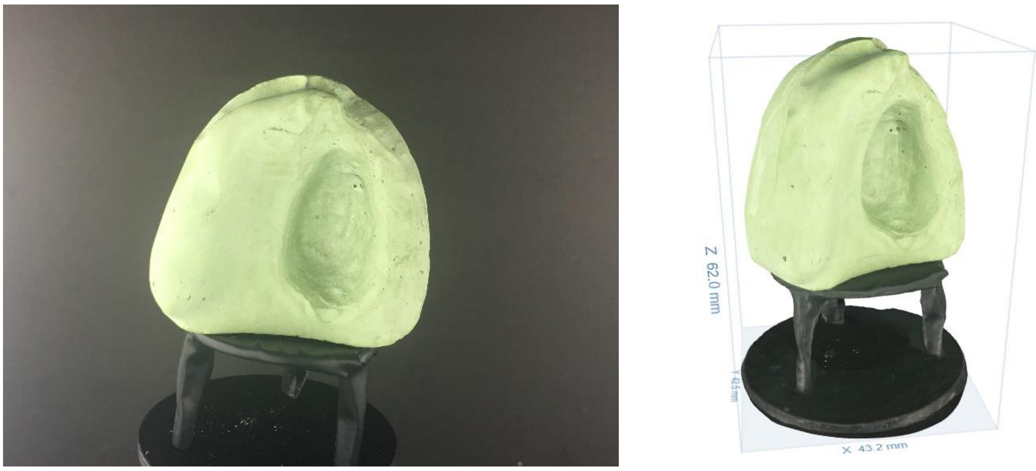

Figure 2: Example of an ideal image with resultant 3D model (Sample 18)

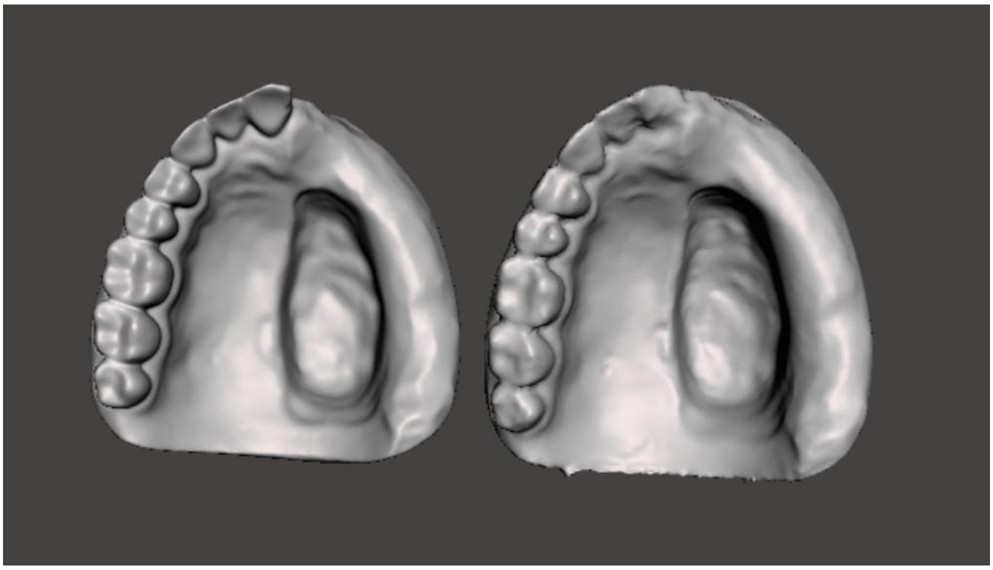

Figure 3: Comparison of laser scanned model (left) vs an ideally taken SPINS model (right)

5.1.7 Other issues during data acquisition

- A tripod base had to be mounted on the flat turntable for the software to be able to separate the apparently stationary turntable from the revolving 3D model.
- Wide-angle action cameras were initially proposed alongside smartphones for image capture, but a subsequent pilot test demonstrated substantial distortion in the resultant 3D models. Action cameras were therefore discontinued from this study.
- Bluetooth connectivity issues were experienced and likely due to the use of an older generation module (3.0). Using newer generation modules (>5.0) or other forms of wireless communications could resolve the issue in future improvements.
- Latency of cloud transfer and (less frequently) image corruption was experienced on 4G network. This was resolved by utilising wi-fi connectivity and archiving the 72-image set as a single '.rar' file prior to cloud transfer. This issue can be resolved when 5G connectivity becomes widely available.

5.2 Challenges during CAD design

5.2.1 Issues with normal faces when the model contain holes/ other polygon defects (3-matics)

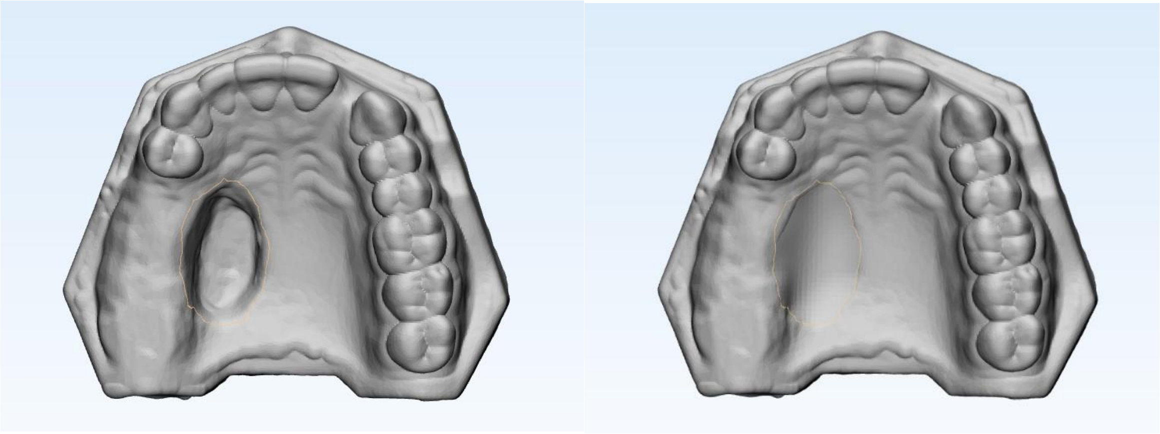

Figure 1: An example of ideal reconstruction for a model with no face errors

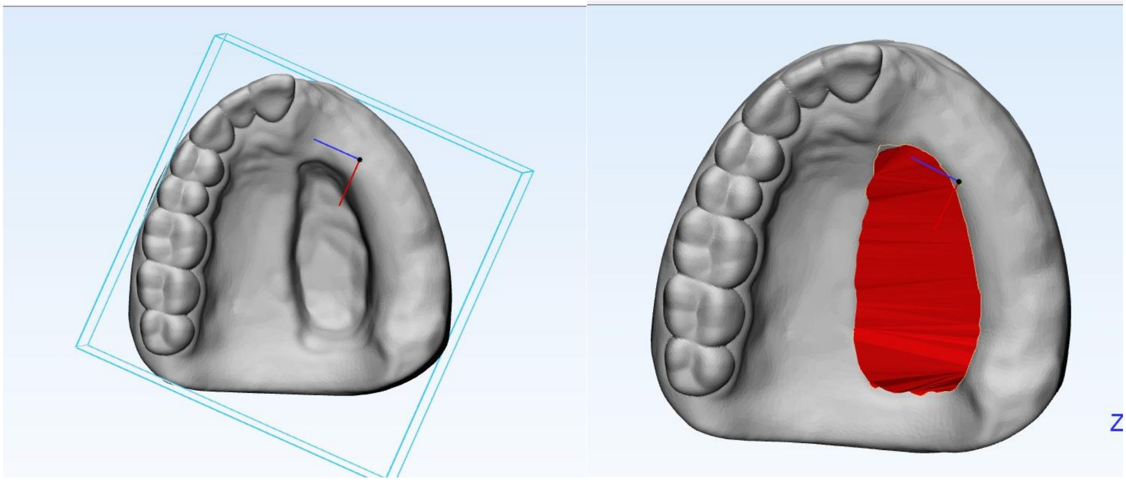

Figure 2: Face errors within the defect resulting in inverted face reconstruction. (Undetectable initially but can be solved by repairing the model prior to prosthesis design)

5.2.2 The need for horizontal peripheral vertices surrounding the defect (Blender – Meshmixer)

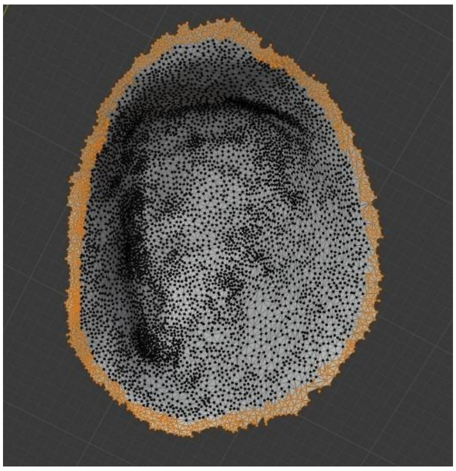

Figure 1: The horizontal peripheral vertices of a moderately sized defect. These vertices are required to act as reconstruction limiting guides for the defect.

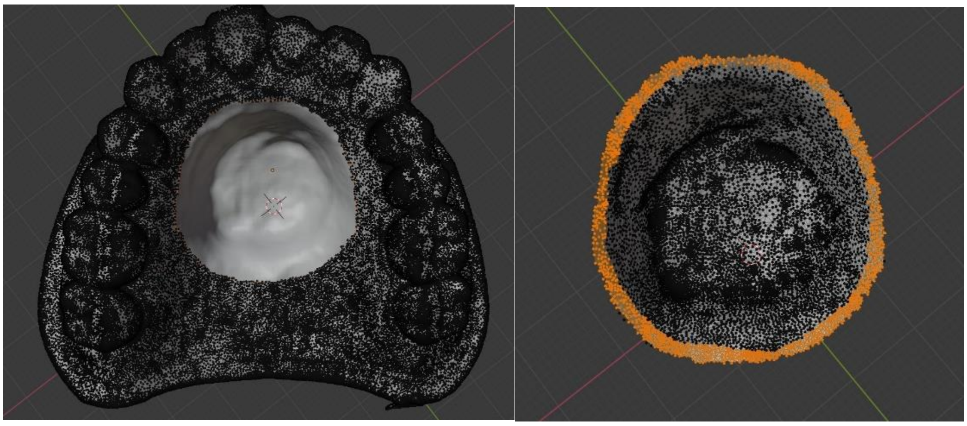

Figure 2: Anterior defects required wider horizontal peripheral vertices to allow for more accurate reconstruction of the slope of the anterior palate

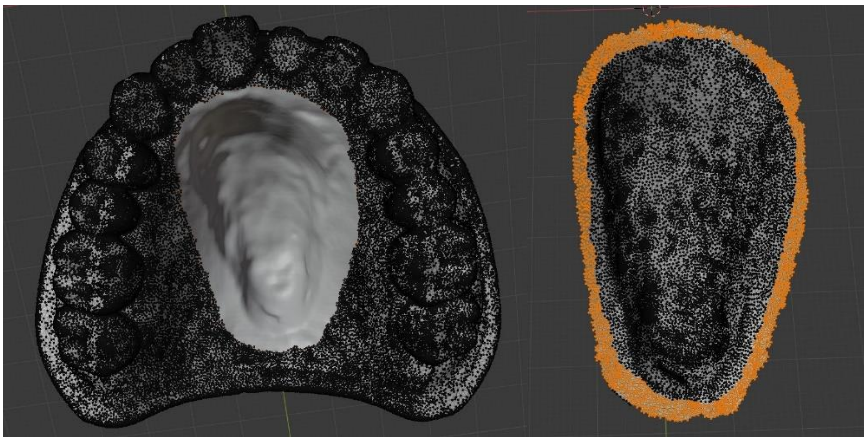

Figure 3: Larger defects required wider peripheral vertices to improve accuracy and avoid major holes during the reconstruction

4.2.3 absence of horizontal peripheral vertices surrounding the defect (Blender – Meshmixer)

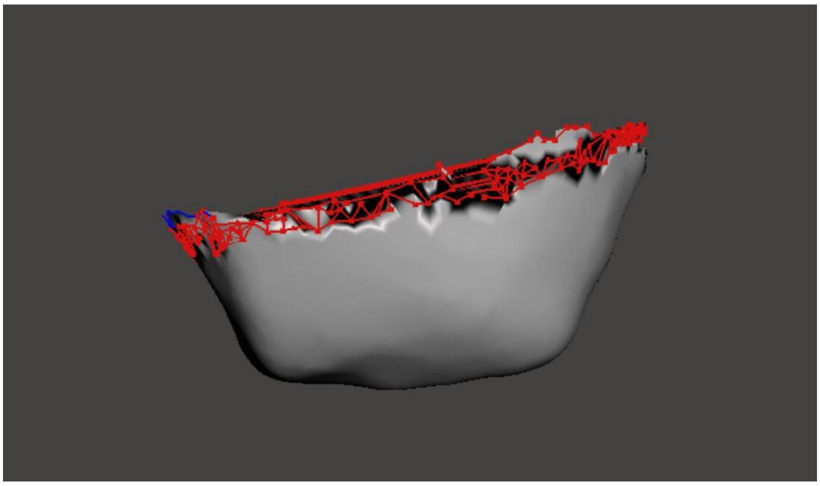

Figure 1: Reconstruction leaves out free vertices on the outer third of the defect which are then recorded as anomalies

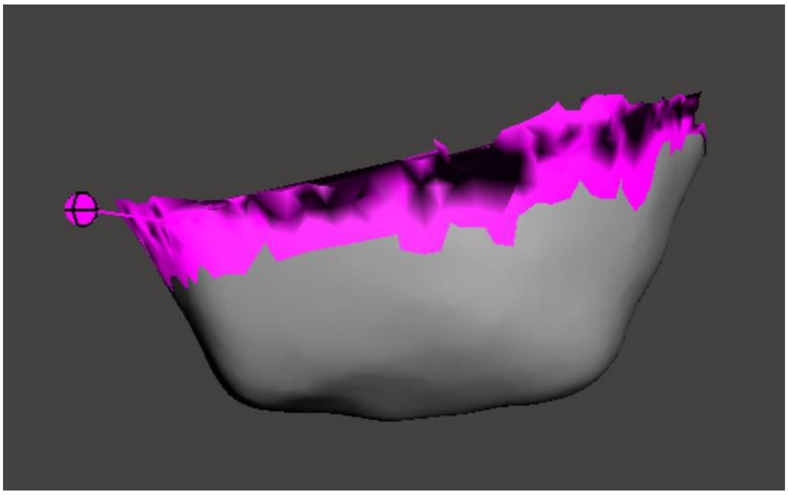

Figure 2: Inspector tool records the anomaly and disregards the actual extent of the defect

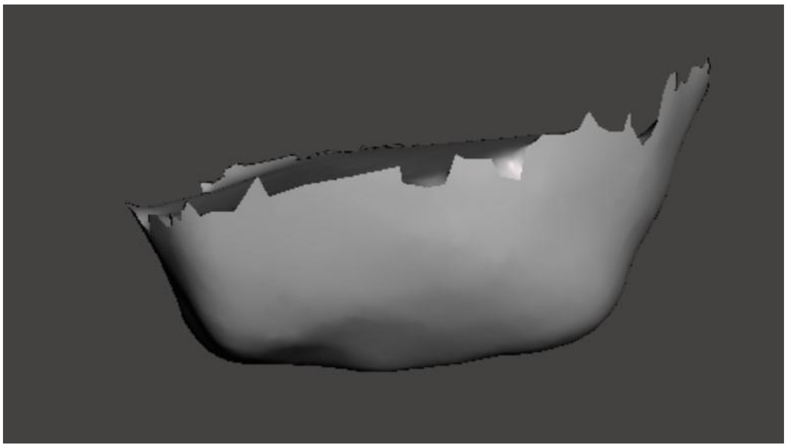

Figure 3: Inspector tool automatically removes the anomaly. Solution: keep adequate peripheral horizontal vertices.

5.2.4 Incomplete selection of horizontal peripheral vertices (Blender – Meshmixer)

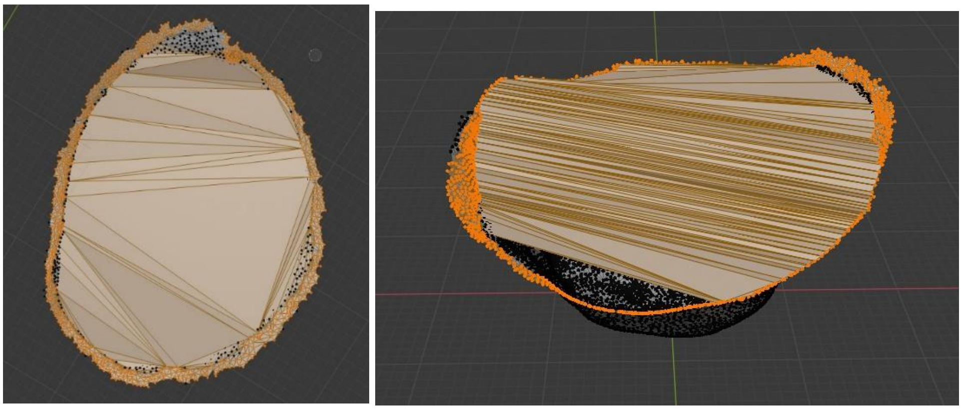

Figure 1: Inadequate selection (at the top) resulted in a hole in reconstruction.

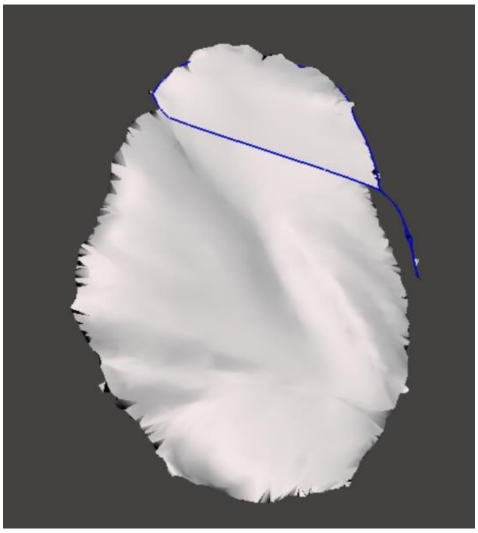

Figure 2: Inspector tool creates an extra layer over the defect in addition to filling the hole

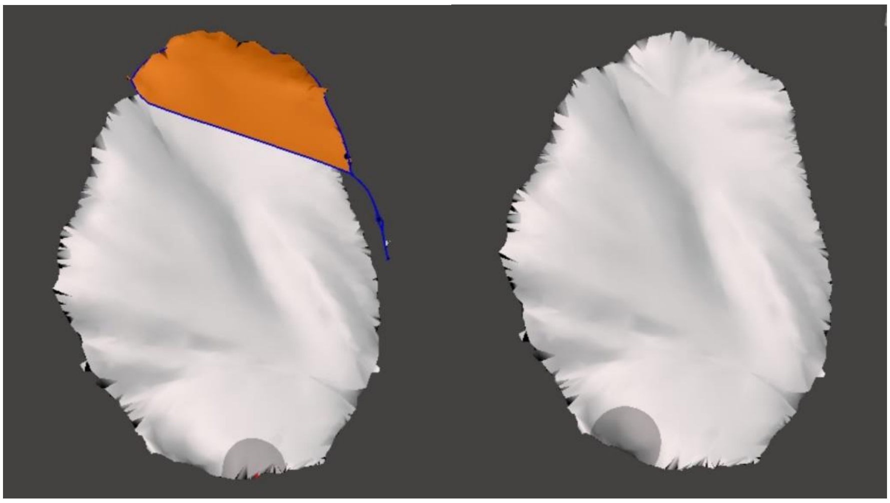

Figure 3: Can be solved by removing the extra layer using *select* and *discard (X)*. This can also be done for surplus peripheral flanges prior to repairing all defects

5.2.5 unconnected/excess peripheral vertices surrounding the defect (Blender – Meshmixer)

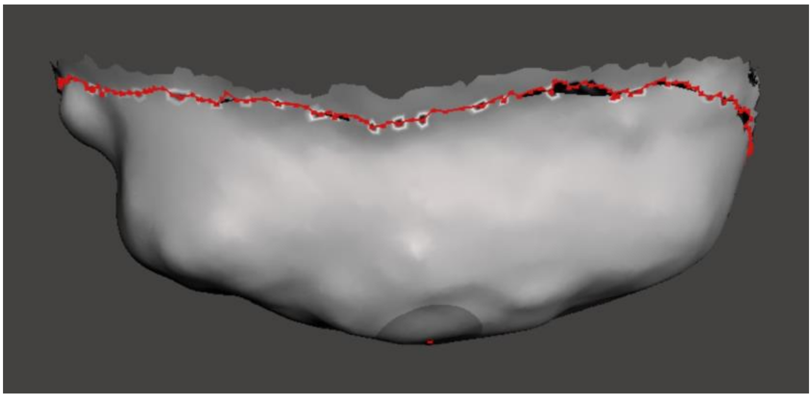

Figure 1: the excess, unconnected vertices are treated as the margins of a second defect above the original reconstruction

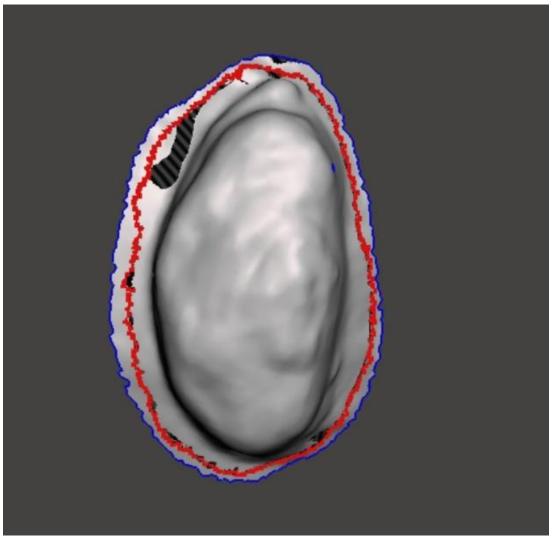

Figure 2: This can be caused by inadequate removal of the excess margins/ failure of hole repair in Blender workflow

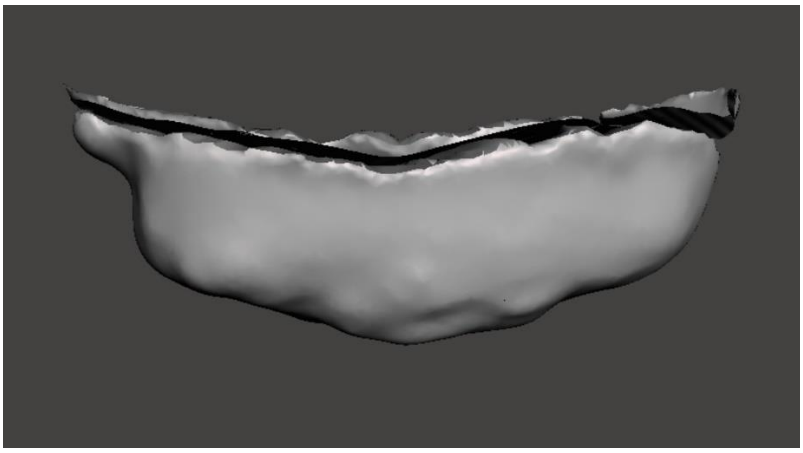

Figure 3: This creates an additional layer above the original bulb if auto-repaired without removing the excess margin

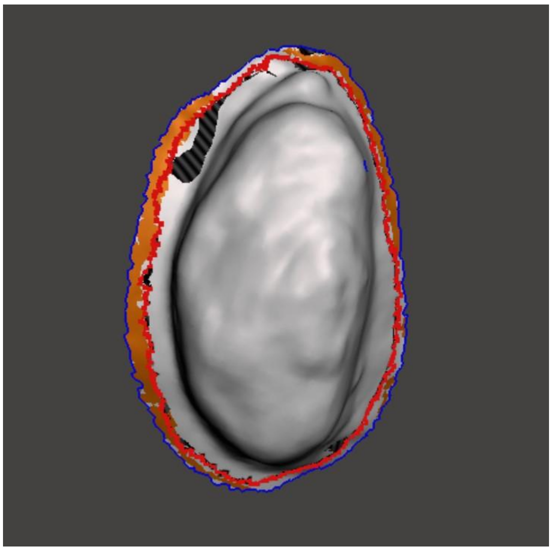

Figure 4: To solve the issue, select and discard some of the excess outside the red ring

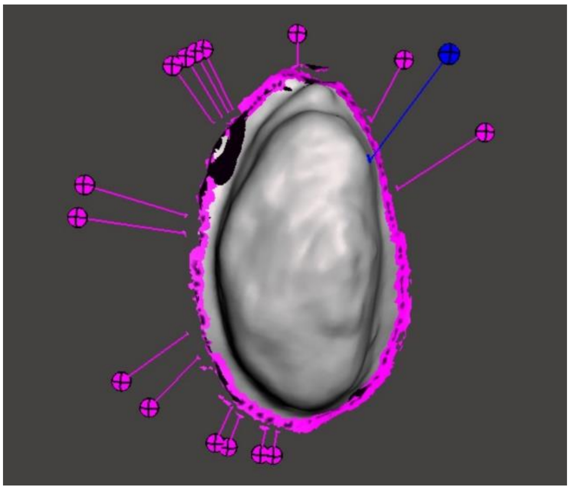

Figure 5: Then the inspector tool will automatically detect and repair the rest of it

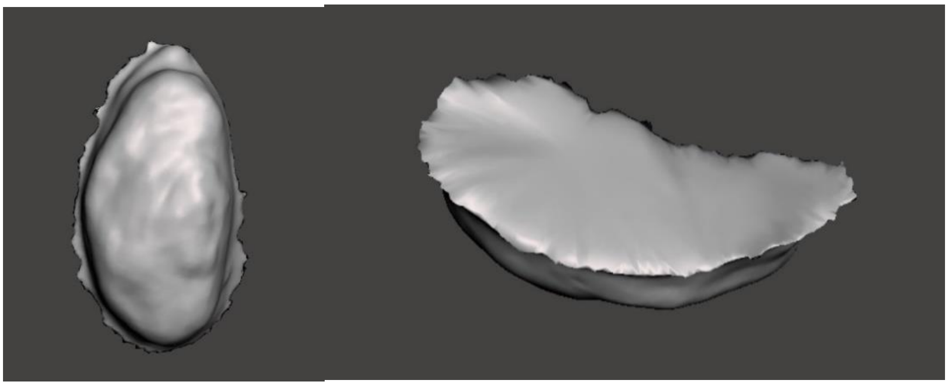

Figure 6: Doing so will solve the issue

5.2.6 Faces are not generated when model is imported into Meshmixer from Blender

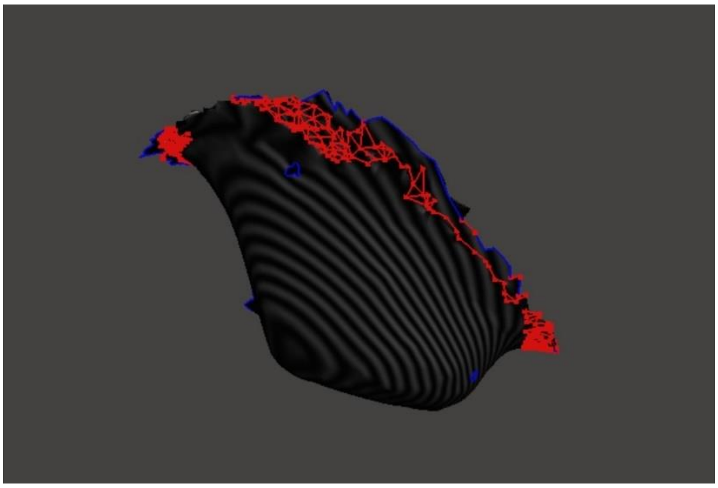

Figure 1: Faces are not generated if 'flip normal' function was not applied in Blender

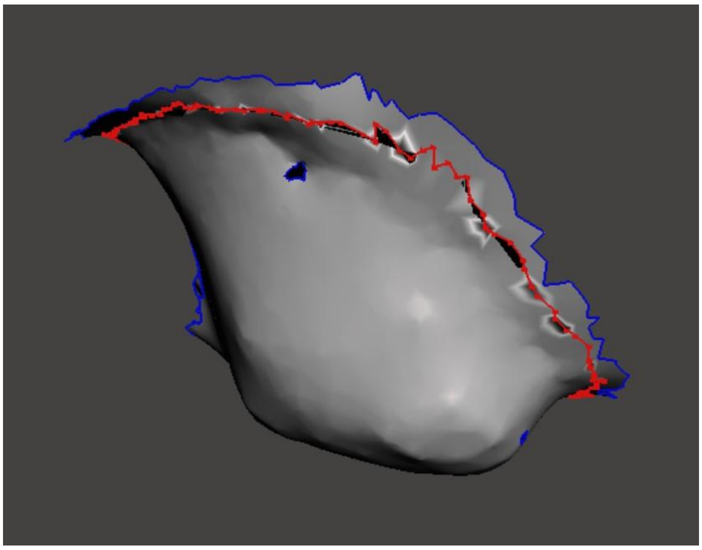

Figure 2: Go back to Blender and carry out all the steps from the beginning. *Flip normal* where indicated to fix the issue

5.3 Inaccuracies on SPINS model carried over to CAD design

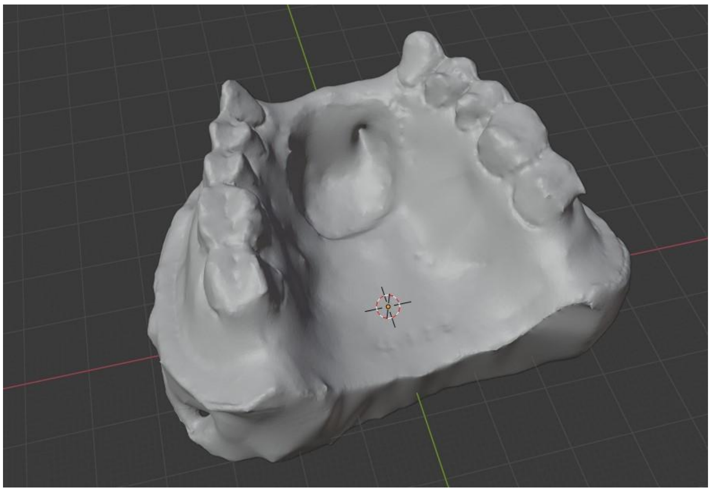

Figure 1: Inaccuracies in the mesh which will affect the final prosthetic outcome

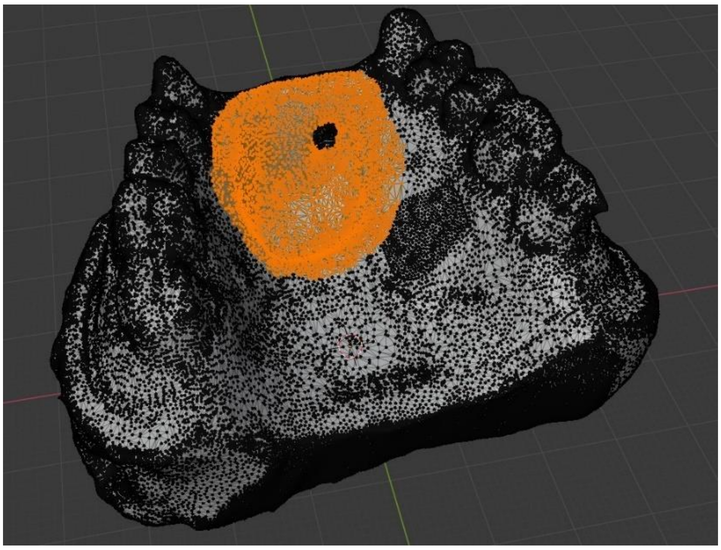

Figure 2: Select around the defect and separate. The unselected region will be treated as a hole

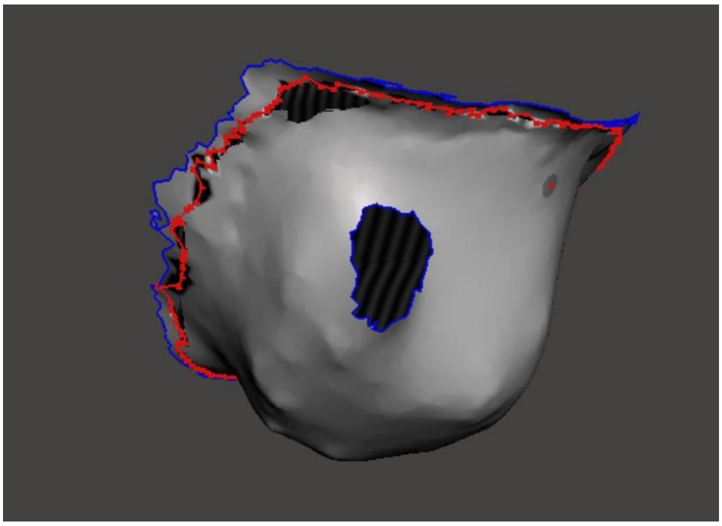

Figure 3: Import to Meshmixer and use analyze tool to auto-repair the hole

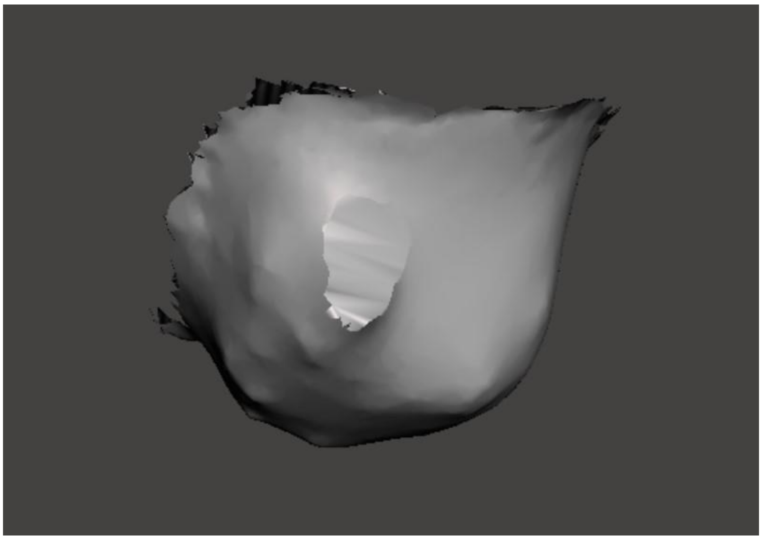

Figure 4: After auto-repair proceed to sculpt, to smoothen the surface

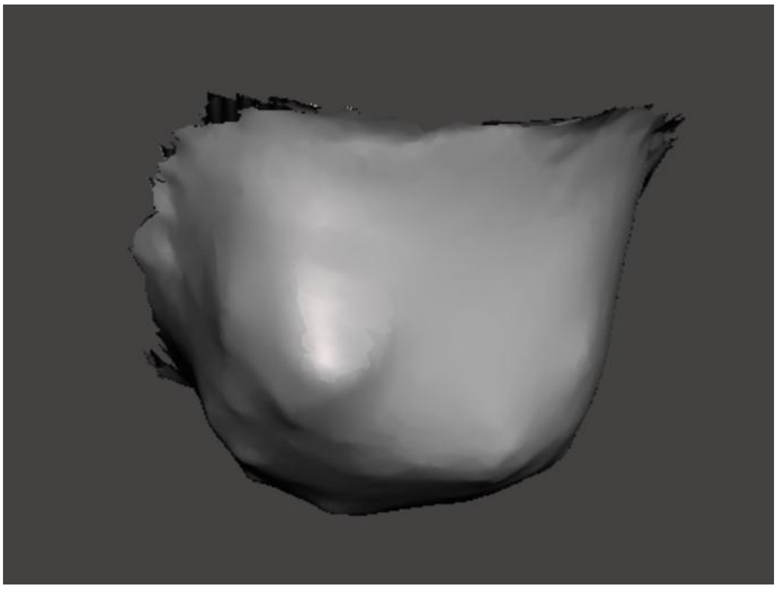

Figure 5: final outcome after sculpt
