# Supplementary material for: Development and virtual validation of a novel digital workflow to rehabilitate palatal defects by using smartphone-integrated stereophotogrammetry (SPINS)
Source: Sci Rep. 2021 Apr 19;11:8469. doi: 10.1038/s41598-021-87240-9 (PMC8055911; doi:10.1038/s41598-021-87240-9)
Supplement: Supplementary file 2 — Supplementary Information 2. [file 41598_2021_87240_MOESM2_ESM.docx]

**SUPPLEMENTARY B: ADDITIONAL TABLES AND RAW DATA**

Contents

Phase A & B 2

Table 1: Pilot data of comparison of 6 different smartphone cameras on outcomes in SPINS 2

Table 2: Statistical comparison of virtual property differences with different smartphones 3

HD & overlapping area discordance data for phase A & B 4

Phase C 10

Table 3: Data on mesh surface area (mm^2^) for outcomes on SPINS and Laser scan 10

Table 4: Data on mesh Volume (mm^3^) for outcomes on SPINS and Laser scan 11

Table 5: Hausdorff’s Distance (mm) comparison SPINS vs Laser Scan 12

Table 6: Dice similarity co-efficient comparison SPINS vs Laser Scan 13

Phase D 15

Calibration reports of 3-matics command parameters 15

Table 7: Data on mesh surface area (mm^2^) for prosthetic bulbs of Set A, B and C 17

Table 8: Data on mesh Volume (mm^3^) for prosthetic bulbs of Set A, B and C 18

Table 9: Hausdorff’s Distance (mm) values of Set B and Set C 19

HD & overlapping area discordance data for phase D 20

Set B 20

Set C 26

Table 10: Dice similarity co-efficient values for Set B and Set C 32

# Phase A & B

## Table 1: Pilot data of comparison of 6 different smartphone cameras on outcomes in SPINS

|  | **Model** | **Smartphones** | | | | | |
| --- | --- | --- | --- | --- | --- | --- | --- |
|  |  | **smartphone 1** | **Smartphone 2** | **smartphone 3** | **smartphone 4** | **smartphone 5** | **smartphone 6** |
| **mesh surface area (mm^2^)** | 2 | 15814.8427 | 14573.343750 | 14360.958984 | 14673.780273 | 15589.642578 | 15198.484375 |
|  | 18 | 8771.906250 | 9822.003906 | 9503.373047 | 9326.047852 | 10931.781250 | 9360.908203 |
| **virtual volume (mm^3^)** | 2 | 97318.6 | 87249.9 | 84906.6 | 85942.4 | 93499.2 | 91709.5 |
|  | 18 | 39138.0 | 41485.4 | 43227.2 | 42489.5 | 54897.6 | 46265.4 |
| **Inter-point discrepancy (mm) by Hausdorff’s Distance (HD)*** | 2 | 0.473198 | 0.241864 | 0.422581 | 0.36816 | 0.117718 | 0.043685 |
|  | 18 | 0.67712 | 0.23463 | 0.03515 | 0.25081 | 0.93200 | 0.12377 |
| **Spatial overlap by Dice Similarity coefficient (DSC)*** | 2 | 0.95870 | 0.93242 | 0.95975 | 0.96815 | 0.94942 | 0.97758 |
|  | 18 | 0.91843 | 0.92048 | 0.95825 | 0.90079 | 0.86840 | 0.93561 |

* HD and DSC carried out with laser scanned samples of 2 & 18 being held as reference respectively

## Table 2: Statistical comparison of virtual property differences with different smartphones

| **Mesh Surface Area (MSA)** | | | | |
| --- | --- | --- | --- | --- |
| **Smartphones** | | ***ꭓ^2^*-stat** | | ***P*-value*** |
| 1, 2 3, 4, 5 and 6 | | 0.923 | | 0.969 |
| **Virtual Volume (VV)** | | | | |
| **Smartphones** | | ***ꭓ^2^*-stat** | | ***P*-value*** |
| 1, 2, 3, 4, 5 and 6 | | 1.231 | | 0.942 |
| **Interpoint Discrepancy by Hausdorff’s Distance**** | | | | |
|  | **Mean HD (mm)** | | | |
| Smartphone 1 | 0.575 | | | |
| Smartphone 2 | 0.238 | | | |
| Smartphone 3 | 0.228 | | | |
| Smartphone 4 | 0.309 | | | |
| Smartphone 5 | 0.525 | | | |
| Smartphone 6 | 0.084 | | | |
| **Spatial overlap by Dice Similarity Coefficient**** | | | | |
|  | **Mean DSC** | | **Percentage DSC***** | |
| Smartphone 1 | 0.944 | | 94.4% | |
| Smartphone 2 | 0.926 | | 92.6% | |
| Smartphone 3 | 0.959 | | 95.9% | |
| Smartphone 4 | 0.935 | | 93.5% | |
| Smartphone 5 | 0.909 | | 90.9% | |
| Smartphone 6 | 0.957 | | 95.7% | |

* Kruskal Wallis one-way test, significance at 0.05. Parametric assumptions for Mesh Surface Area (MSA) and Virtual Volume (VV) were not met. Kolmogorov-Smirnov test was significant (P=0.06). Median and IQR not reported due to small dataset

** HD and DSC carried out with laser scanned samples of 2 & 18 being held as reference. The values were approximated to 3 decimal places

***Percentage Similarity Coefficient derived from the formula %DSC= Mean DSC X 100

## HD & overlapping area discordance data for phase A & B

Smartphone 1

**
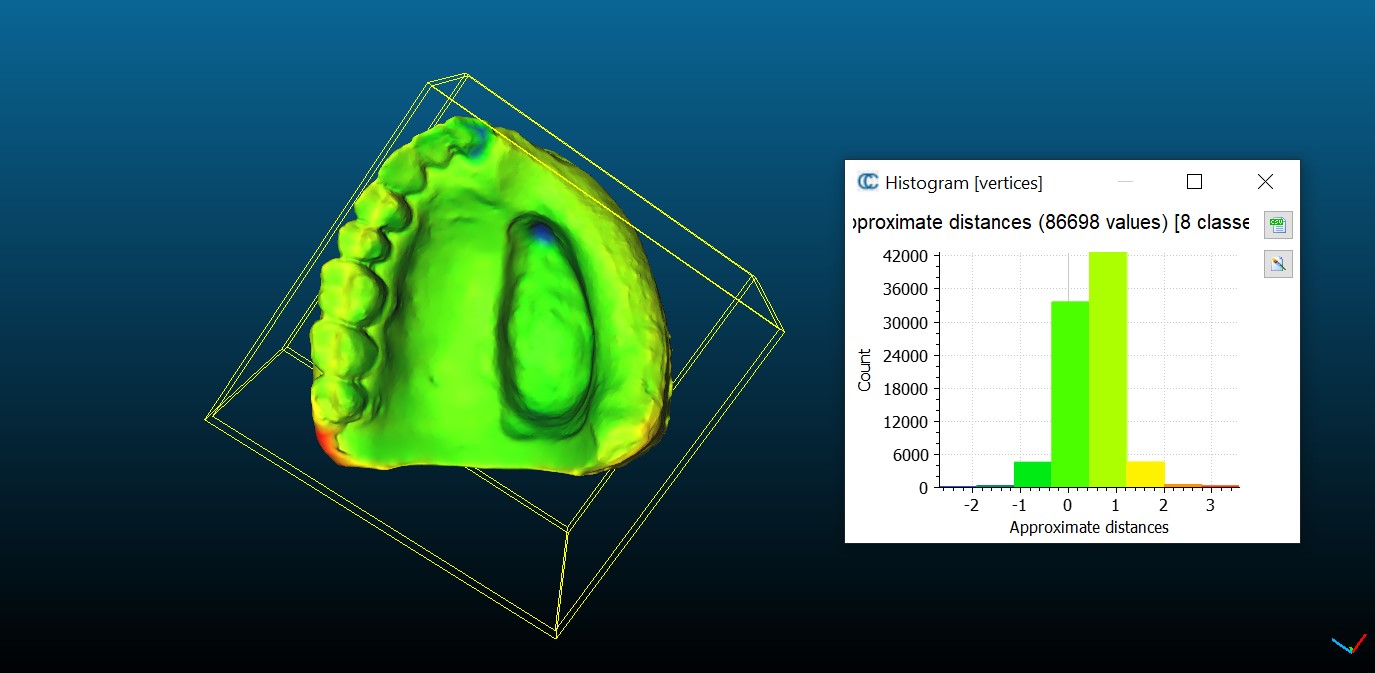

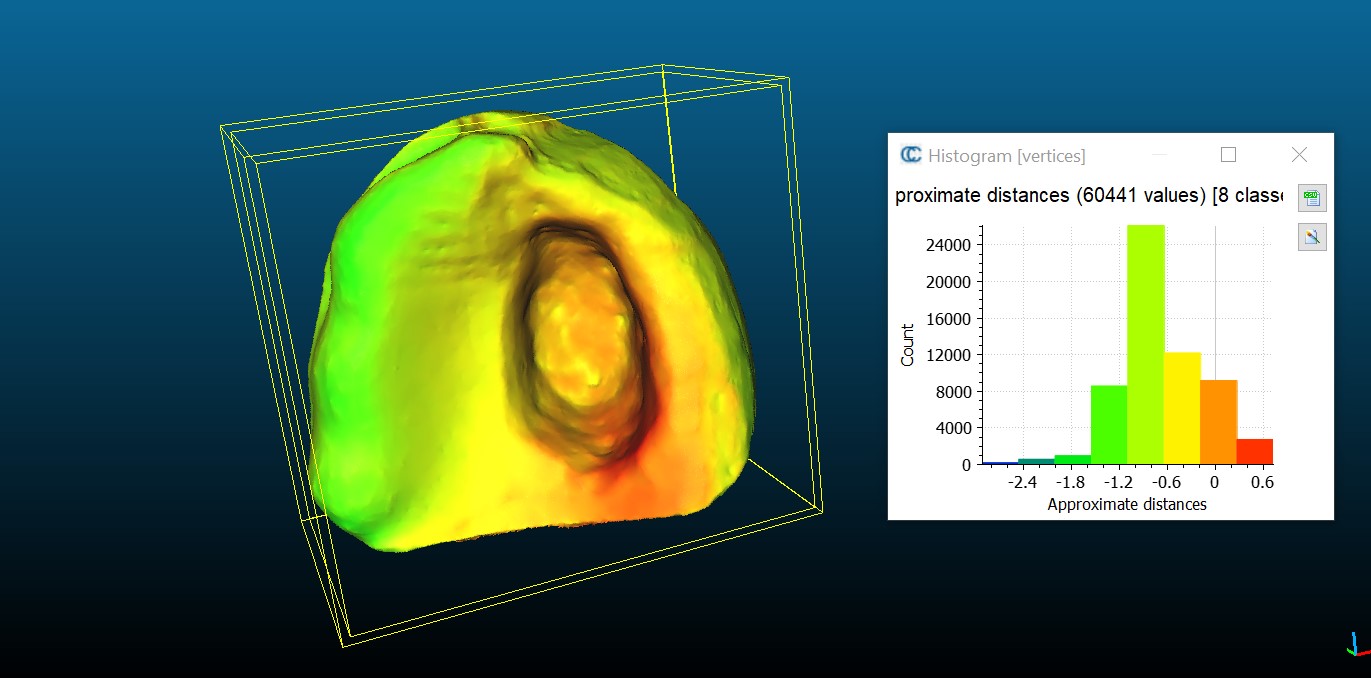
**

Smartphone 2

**
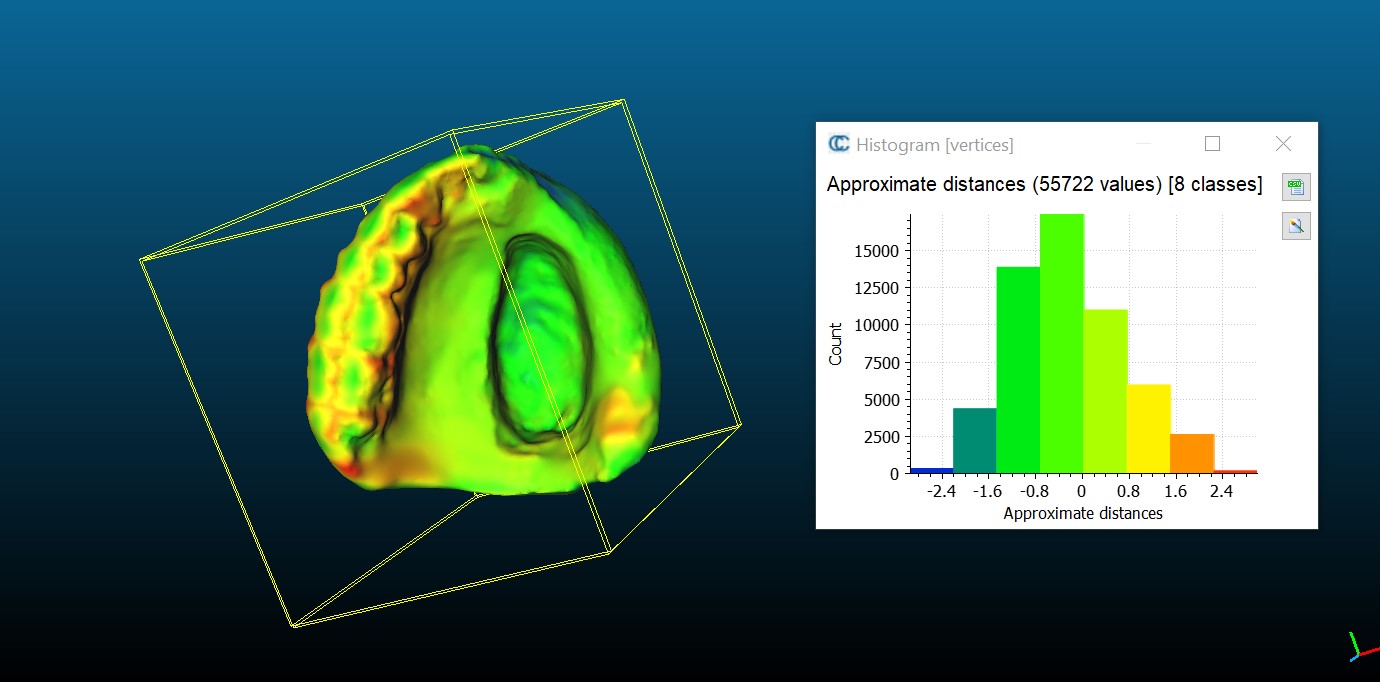

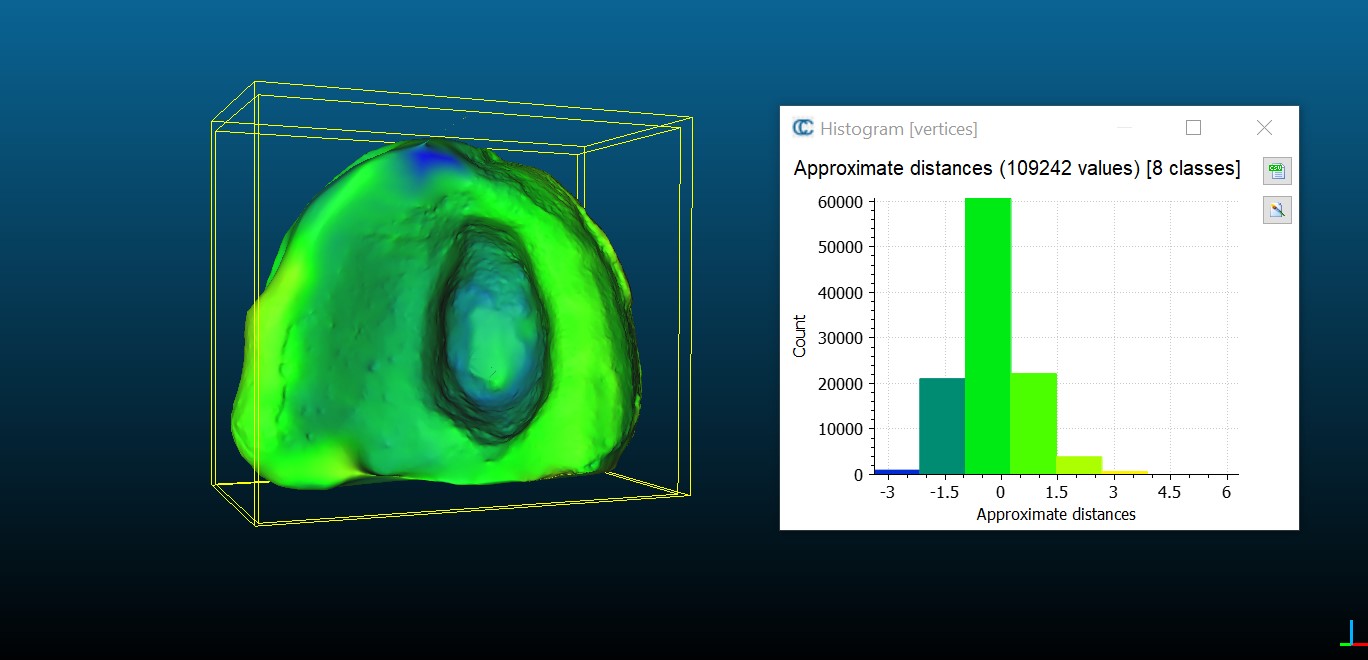
**

Smartphone 3

**
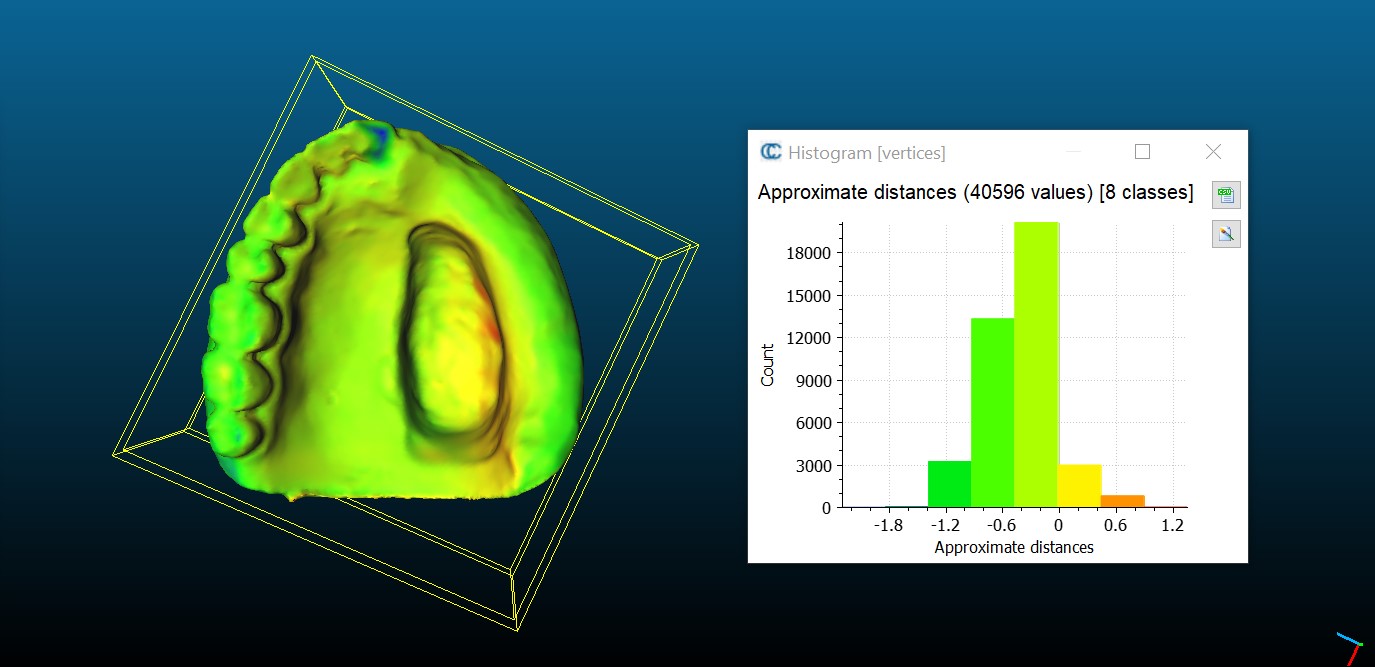

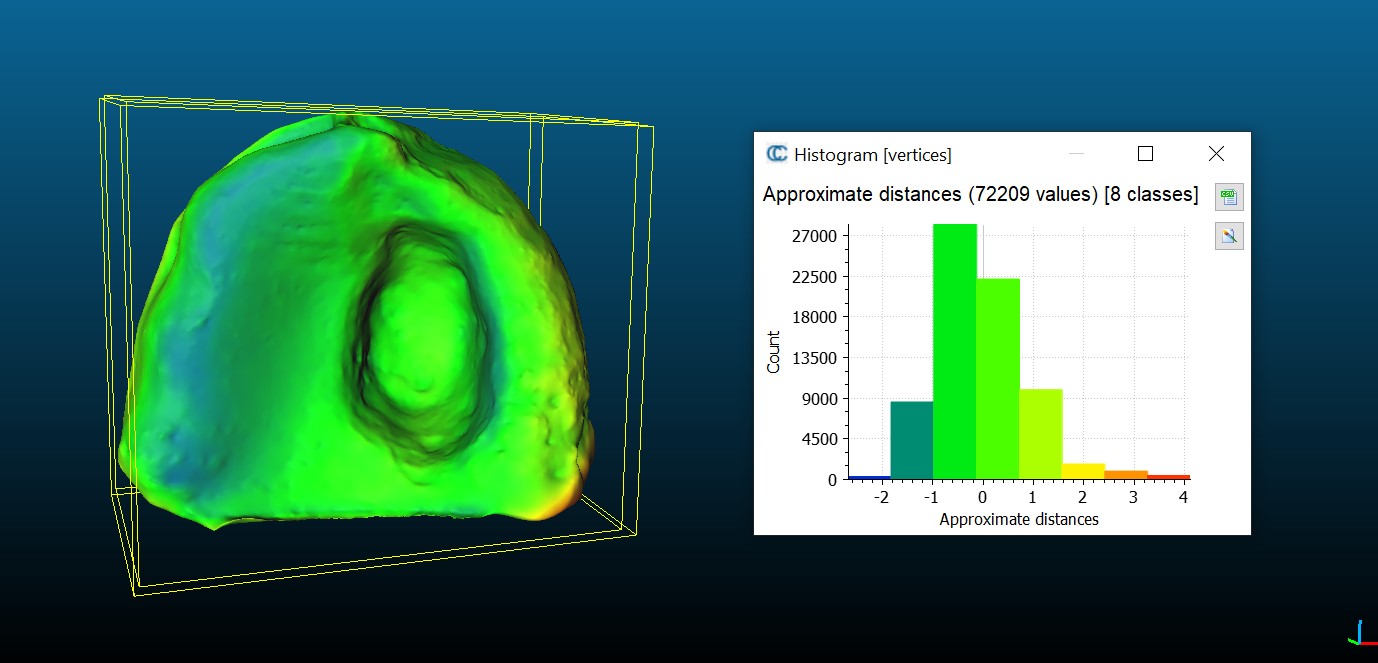
**

Smartphone 4

**
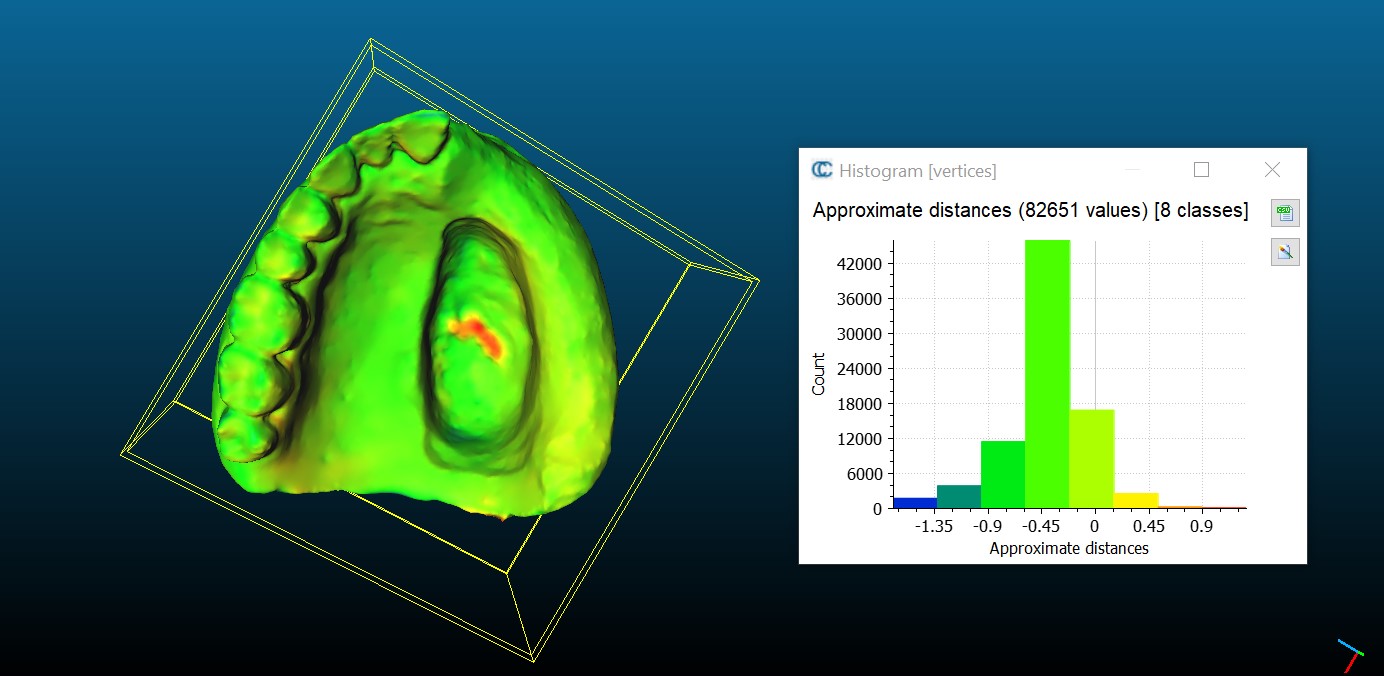

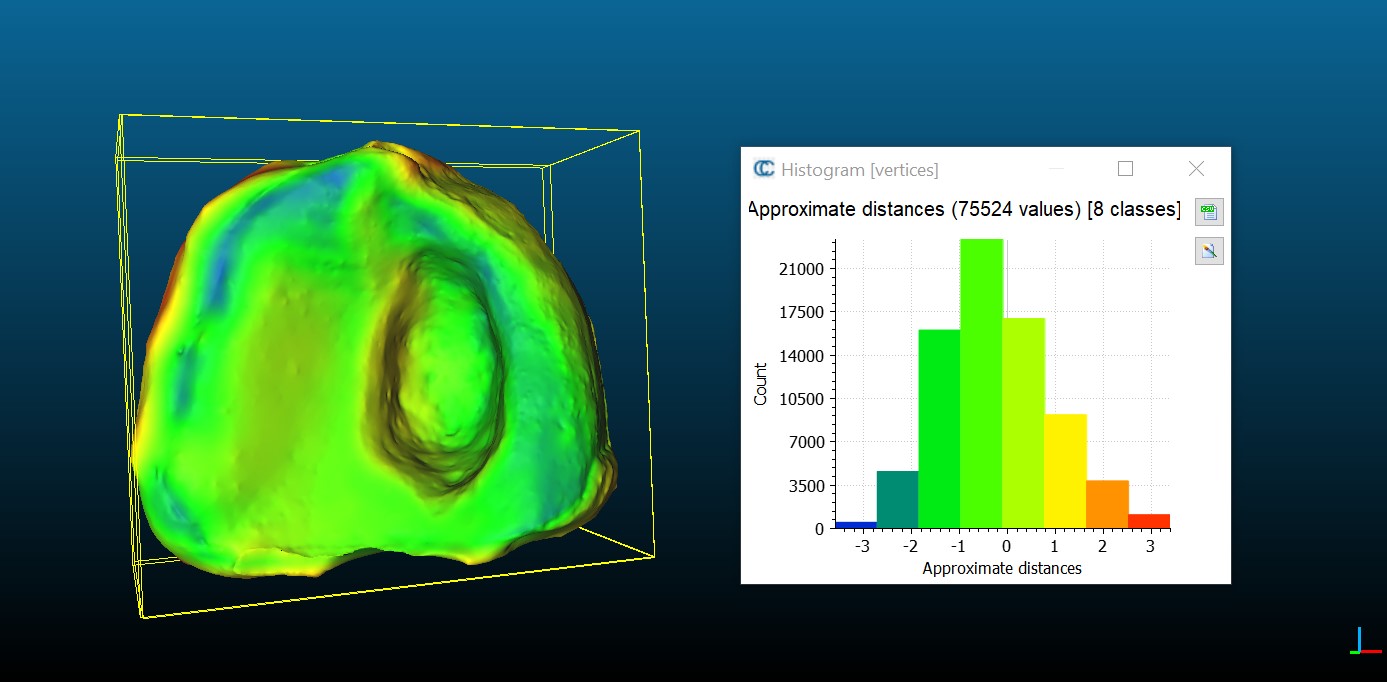
**

Smartphone 5

**
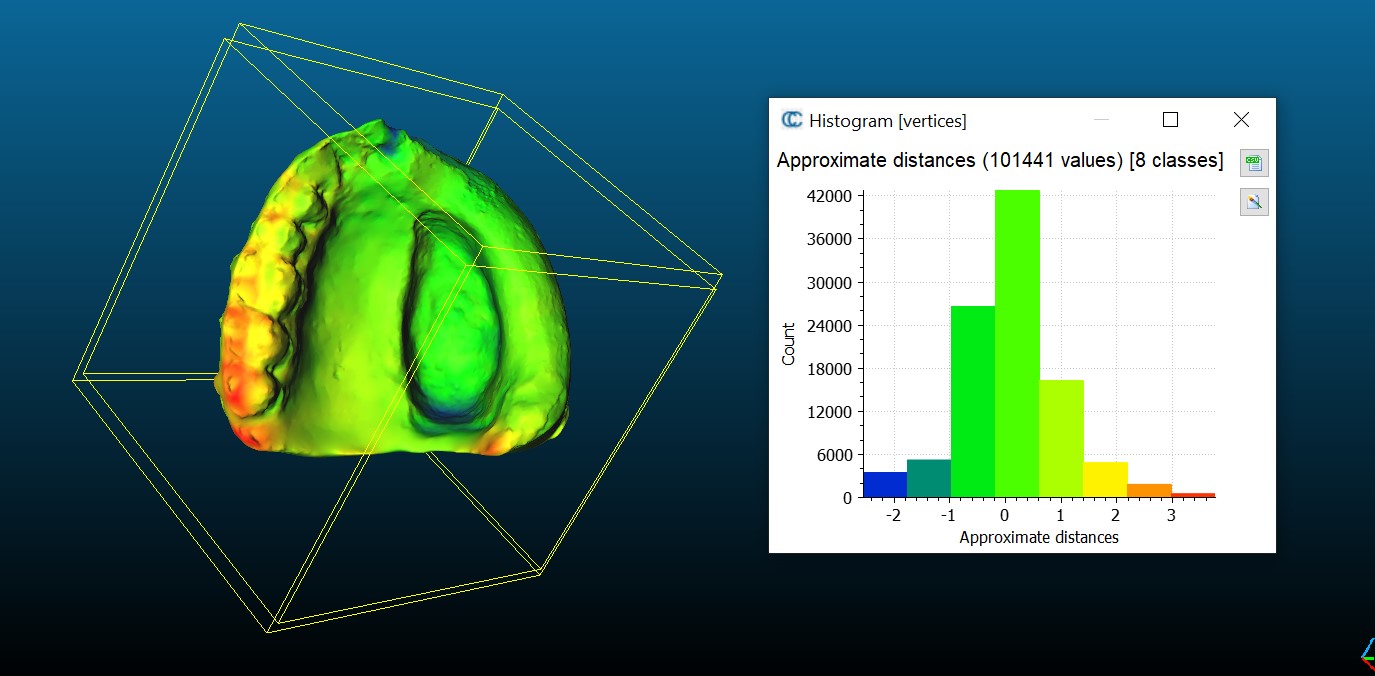

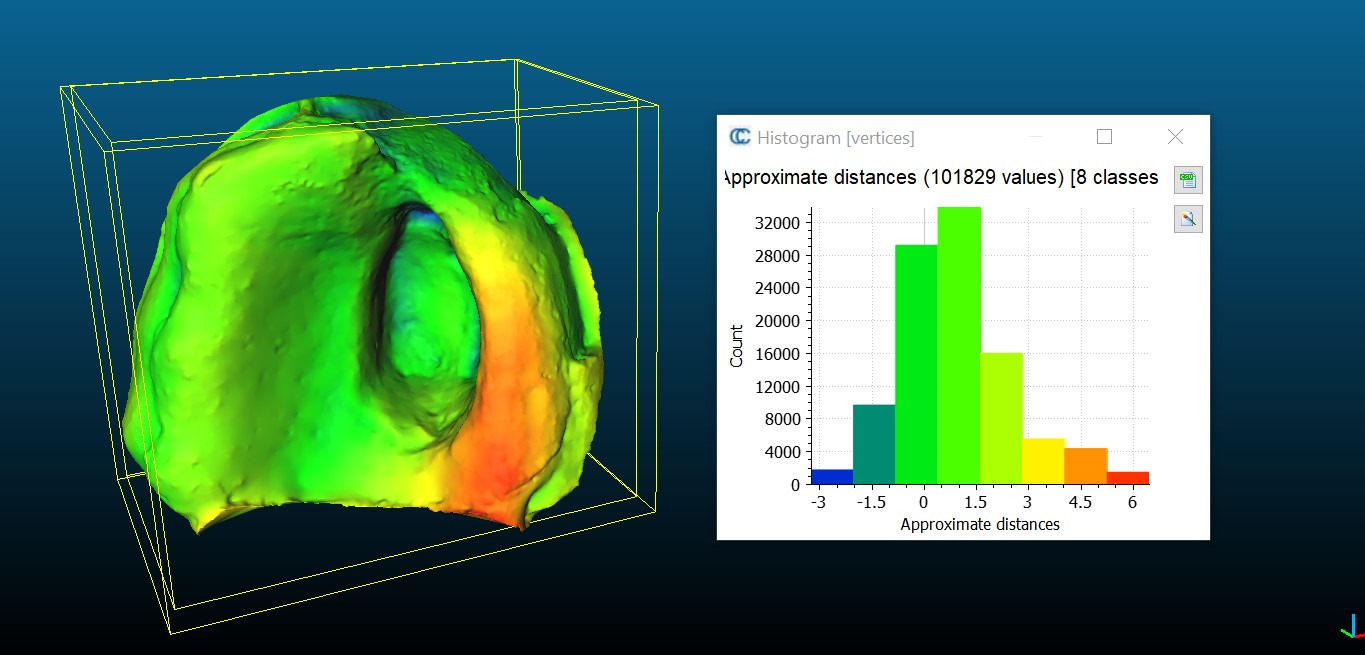
**

Smartphone 6

**
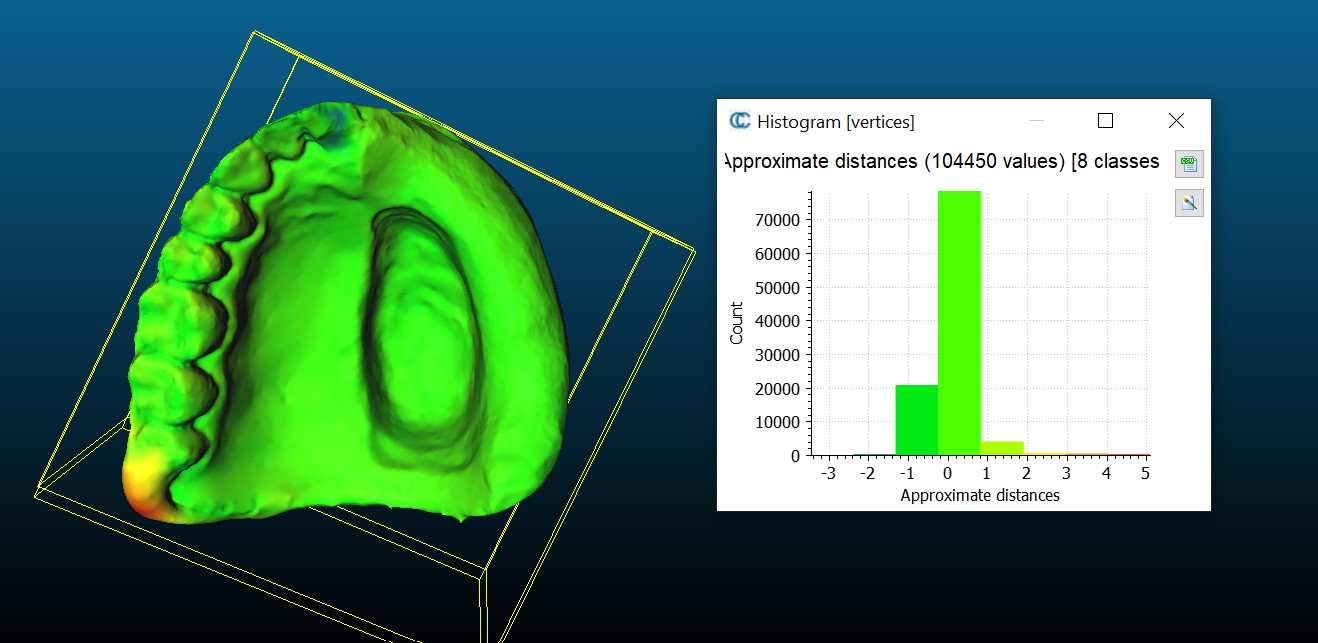

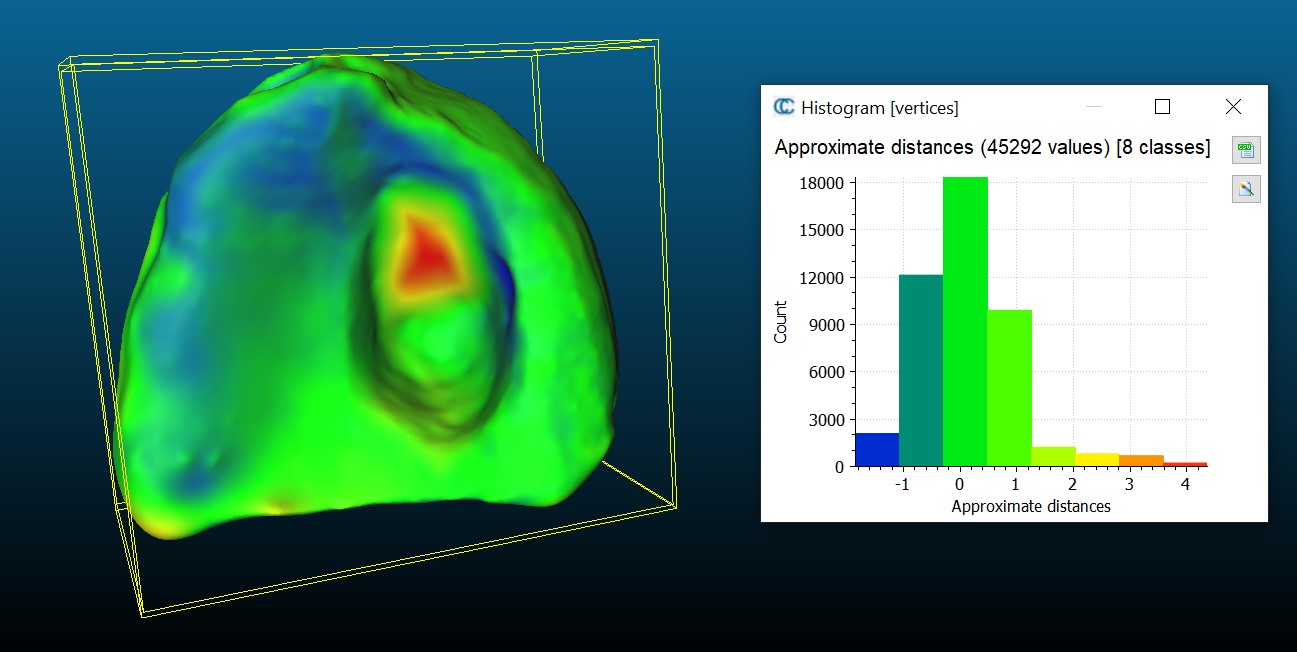
**

# Phase C

## Table 3: Data on mesh surface area (mm^2^) for outcomes on SPINS and Laser scan

| **Model Number** | **SPINS** | **Laser Scan** |
| --- | --- | --- |
| 1 | 12663.196289 | 13346.339844 |
| 2 | 14673.780273 | 15066.920898 |
| 3 | 15032.512695 | 14896.963867 |
| 4 | 12593.956055 | 13526.327148 |
| 5 | 14366.956055 | 13166.641602 |
| 6 | 14837.927734 | 13839.636719 |
| 7 | 13852.838867 | 13440.957031 |
| 8 | 15221.167969 | 14923.341797 |
| 9 | 15569.631836 | 14542.904297 |
| 10 | 15134.981445 | 15210.894531 |
| 11 | 15279.843750 | 15632.036133 |
| 12 | 15362.867188 | 13303.266602 |
| 13 | 13682.073242 | 14292.247070 |
| 14 | 15827.028320 | 16019.908203 |
| 15 | 15191.708008 | 14394.273438 |
| 16 | 13788.758789 | 14556.639648 |
| 17 | 14712.434570 | 15401.836914 |
| 18 | 9326.047852 | 9503.620117 |

Computer generated (default) output of 6 decimal places

## Table 4: Data on mesh Volume (mm^3^) for outcomes on SPINS and Laser scan

| **Model Number** | **SPINS** | **Laser Scan** |
| --- | --- | --- |
| 1 | 62999.6 | 68618.0 |
| 2 | 85942.6 | 91313.4 |
| 3 | 83584.4 | 84085.2 |
| 4 | 54202.2 | 61464.2 |
| 5 | 67639.8 | 60085.7 |
| 6 | 81184.4 | 72884.6 |
| 7 | 69749.8 | 65585.1 |
| 8 | 76763.2 | 73943.8 |
| 9 | 70220.9 | 66274.5 |
| 10 | 71305.9 | 70751.7 |
| 11 | 75587.0 | 76586.1 |
| 12 | 85156.3 | 60472.7 |
| 13 | 72601.6 | 78849.5 |
| 14 | 72212.1 | 75322.7 |
| 15 | 83980.4 | 76360.1 |
| 16 | 65159.0 | 66311.9 |
| 17 | 78863.7 | 82442.2 |
| 18 | 42489.5 | 45435.3 |

Computer generated (default) output of 1 decimal place

## Table 5: Hausdorff’s Distance (mm) comparison SPINS vs Laser Scan

| **Model Number** | **SPINS vs Laser Scan** |
| --- | --- |
| 1 | 0.373528 |
| 2 | 0.368160 |
| 3 | 0.099698 |
| 4 | 0.518712 |
| 5 | 0.826548 |
| 6 | 0.692940 |
| 7 | 0.406433 |
| 8 | 0.318937 |
| 9 | 0.465651 |
| 10 | 0.121910 |
| 11 | 0.053355 |
| 12 | 1.934922 |
| 13 | 0.411541 |
| 14 | 0.179631 |
| 15 | 0.639920 |
| 16 | 0.138587 |
| 17 | 0.168251 |
| 18 | 0.250810 |

Computer generated (default) output of 6 decimal places.

## Table 6: Dice similarity co-efficient comparison SPINS vs Laser Scan

| **Model Number** | **SPINS vs Laser Scan** |
| --- | --- |
| 1 | 0.987968 |
| 2 | 0.968153 |
| 3 | 0.965912 |
| 4 | 0.922773 |
| 5 | 0.905625 |
| 6 | 0.914880 |
| 7 | 0.947169 |
| 8 | 0.909268 |
| 9 | 0.923912 |
| 10 | 0.940121 |
| 11 | 0.916349 |
| 12 | 0.819573 |
| 13 | 0.938769 |
| 14 | 0.954052 |
| 15 | 0.927361 |
| 16 | 0.938617 |
| 17 | 0.950957 |
| 18 | 0.900797 |

***DSC calculation breakdown:***

$$\frac{2*(A\cap B)}{A+B}$$

| Model 1: $\frac{2* 62053.1}{62999.6+62618.0}$ = 0.987968 | Model 10:$\frac{2* 66775.7}{71305.9+70751.7}$ = 0.940121 |
| --- | --- |
| Model 2: $\frac{2* 85805.5}{85942.6+91313.4}$ = 0.968153 | Model 11:$\frac{2* 69721.8}{75587.0+76586.1}$ = 0.916349 |
| Model 3:$\frac{2* 80977.0}{83584.4+84085.2}$ = 0.965912 | Model 12:$\frac{2* 59676.8}{85156.3+60472.7}$ = 0.819573 |
| Model 4:$\frac{2* 53367.0}{54202.4+61464.2}$ = 0.922773 | Model 13:$\frac{2* 71088.8}{72601.6+78849.5}$ = 0.938769 |
| Model 5:$\frac{2* 57835.7}{67639.8+60085.7}$ = 0.905625 | Model 14:$\frac{2* 70377.9}{72212.1+75322.7}$ = 0.954052 |
| Model 6:$\frac{2* 70477.3}{81184.4+72884.6}$ = 0.914880 | Model 15:$\frac{2* 74346.8}{83980.4+76360.1}$ = 0.927361 |
| Model 7:$\frac{2* 64092.5}{69749.8+65585.1}$ = 0.947169 | Model 16:$\frac{2* 61700.4}{65159.0+66311.9}$ = 0.938617 |
| Model 8:$\frac{2* 68516.5}{76763.2+73943.8}$ = 0.909268 | Model 17:$\frac{2* 76697.5}{78863.7+82442.2}$ = 0.950957 |
| Model 9:$\frac{2* 63042.4}{70220.9+66274.5}$ = 0.923912 | Model 18:$\frac{2* 39601.2}{42489.5+45435.3}$ = 0.900797 |

# Phase D

## Calibration reports of 3-matics command parameters

[At default settings – 3matics (Materialise)]

**Model 2:**

HD = 0.113139 (value <0.5mm, hence deemed acceptable)

Area Discordance:


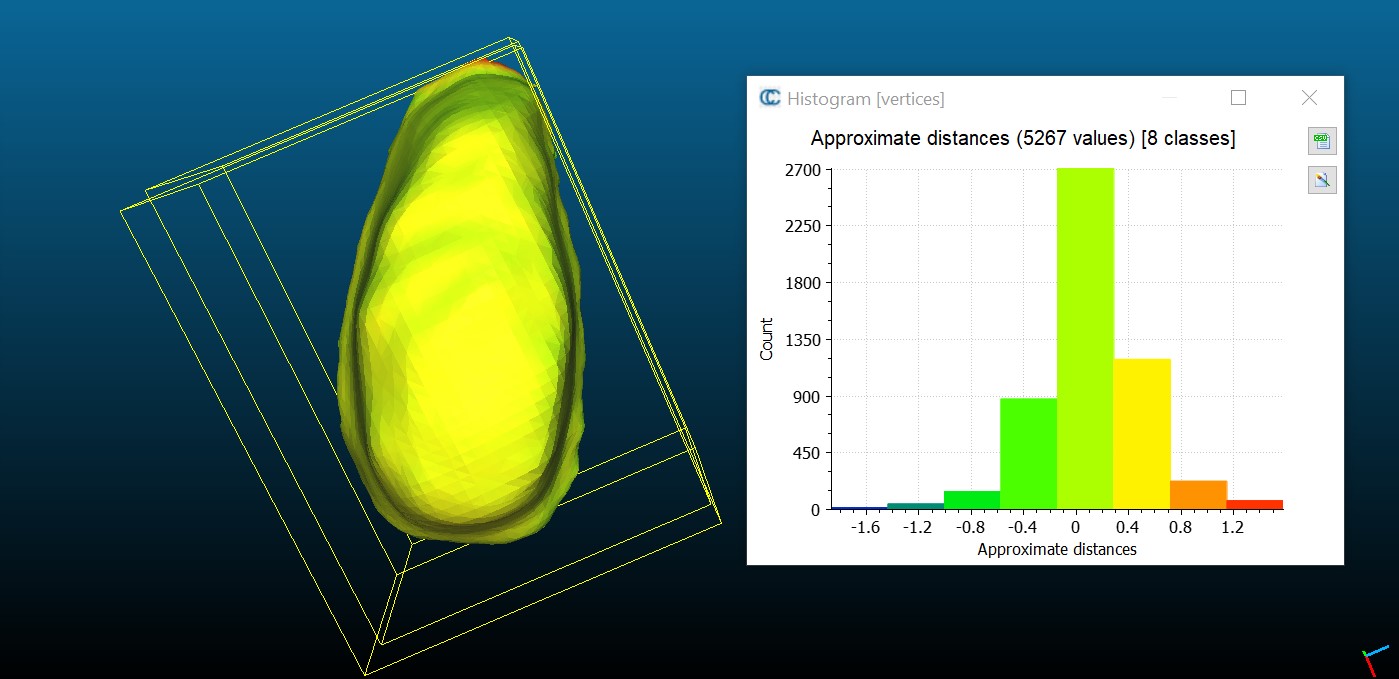

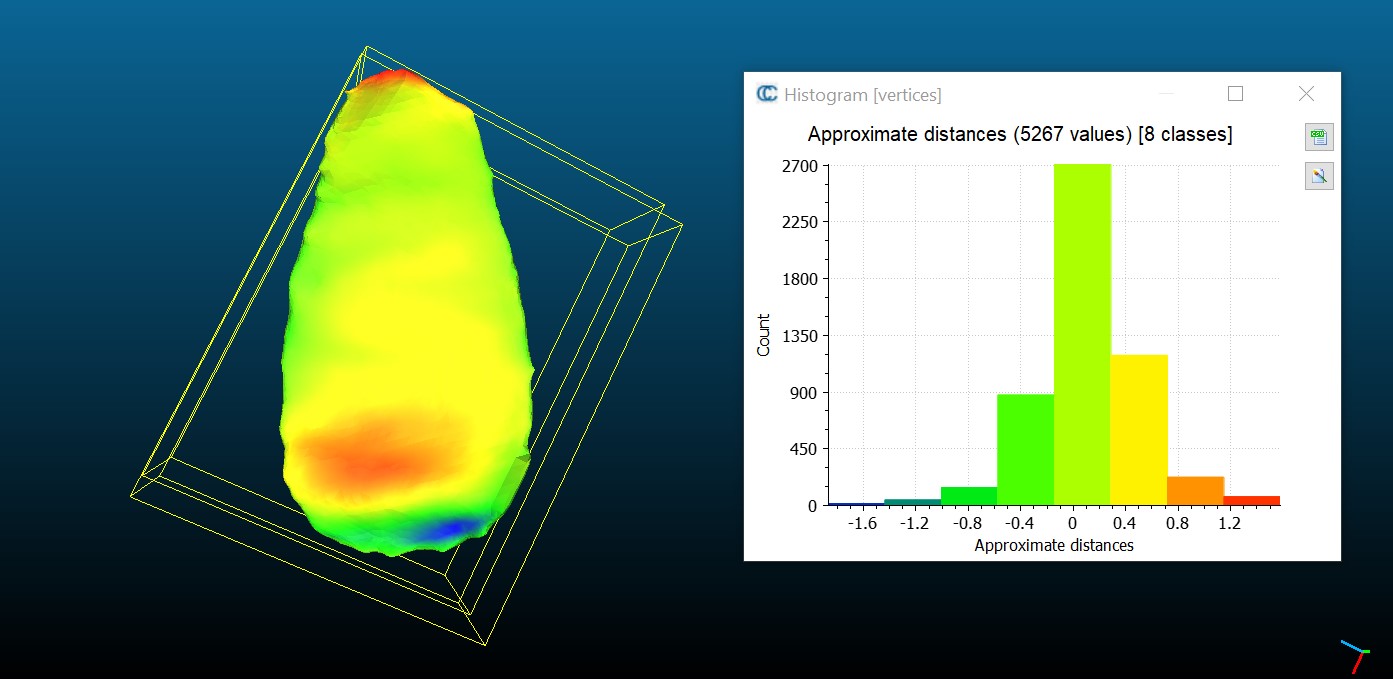


DSC = 0.9461853

Default calibration satisfied >0.7 condition, therefore no modifications were made in parameters

**Model 18:**

HD = 0.041375 (value <0.5mm, hence deemed acceptable)

Area Discordance:


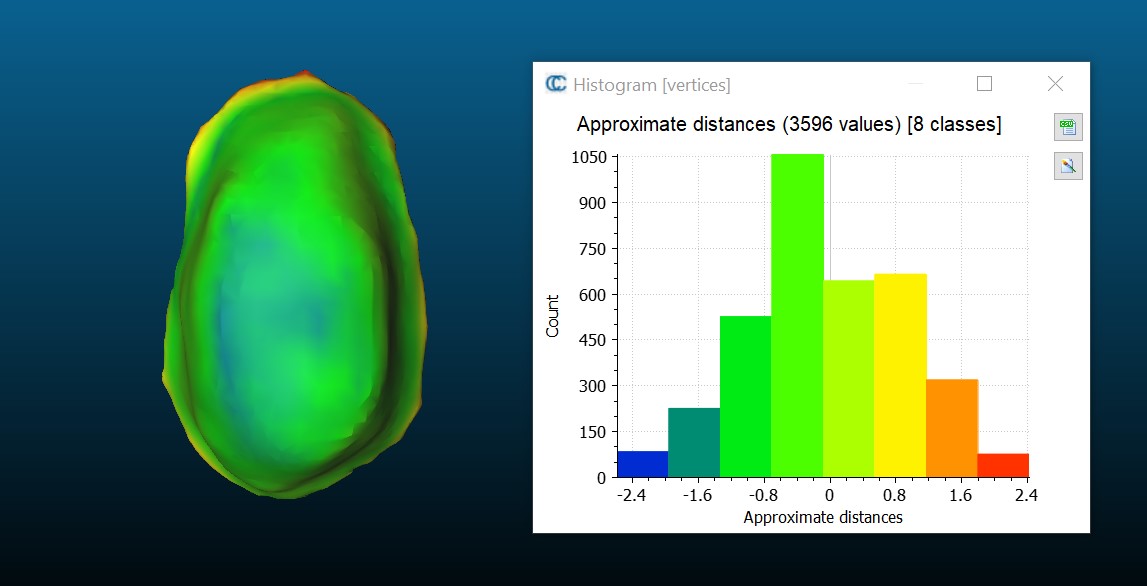


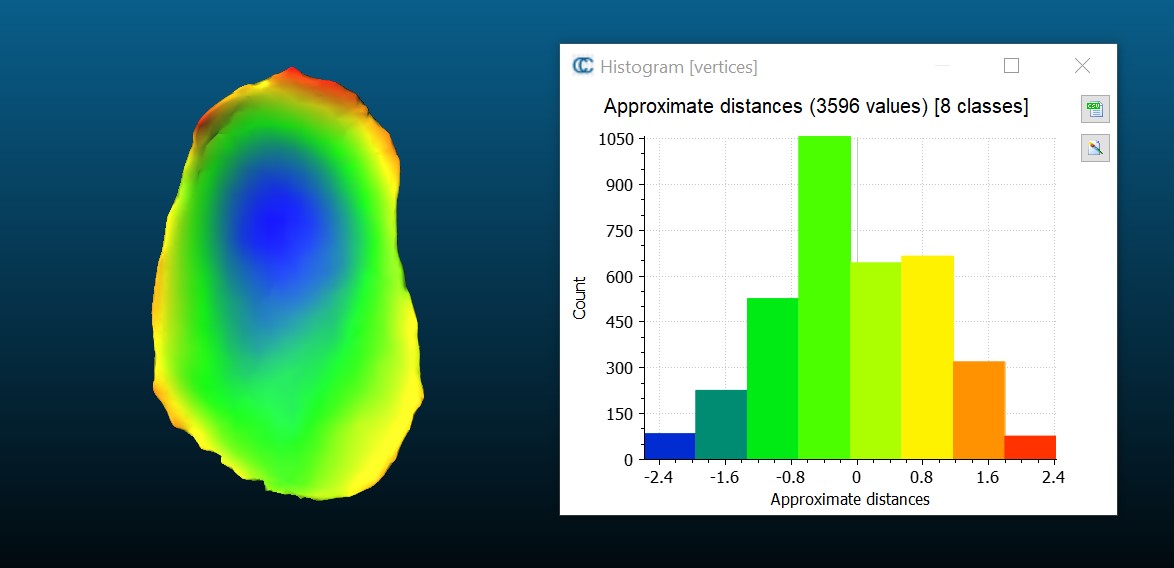


DSC = 0.836741

Default calibration satisfied >0.7 condition, therefore no modifications were made in 3-matics parameters

## Table 7: Data on mesh surface area (mm^2^) for prosthetic bulbs of Set A, B and C

| **Model** | **Set A** | **Set B** | **Set C** |
| --- | --- | --- | --- |
| 1 | 1398.110107 | 1314.050415 | 1218.453857 |
| 2 | 2377.051025 | 2138.978760 | 2096.219971 |
| 3 | 1233.502319 | 1237.814453 | 1293.447510 |
| 4 | 1254.528442 | 1414.324951 | 1364.139893 |
| 5 | 1216.173218 | 1244.994263 | 1238.002319 |
| 6 | 1620.373413 | 1398.393799 | 1337.620361 |
| 7 | 1257.924316 | 1117.688843 | 1084.503296 |
| 8 | 755.744263 | 539.123047 | 625.327209 |
| 9 | 1083.570313 | 1107.966064 | 1114.554077 |
| 10 | 1958.188965 | 1914.635376 | 2005.310547 |
| 11 | 2120.021240 | 2229.107910 | 2029.818726 |
| 12 | 1395.348389 | 1333.301880 | 1420.356079 |
| 13 | 1461.932129 | 1333.085327 | 1329.120239 |
| 14 | 1681.010864 | 1562.270020 | 1617.469849 |
| 15 | 1947.267700 | 1880.720093 | 1926.310791 |
| 16 | 1382.997925 | 1360.551270 | 1124.452026 |
| 17 | 1316.929810 | 1034.520508 | 1115.611084 |
| 18 | 1844.174561 | 1510.234497 | 1580.111450 |

## Table 8: Data on mesh Volume (mm^3^) for prosthetic bulbs of Set A, B and C

| **Model** | **Set A** | **Set B** | **Set C** |
| --- | --- | --- | --- |
| 1 | 2975.47 | 2698.32 | 2435.20 |
| 2 | 6868.75 | 5894.06 | 5770.23 |
| 3 | 1847.84 | 2582.72 | 2660.80 |
| 4 | 1529.83 | 3096.25 | 2713.29 |
| 5 | 2639.87 | 2579.56 | 2983.36 |
| 6 | 2519.89 | 2821.32 | 2764.05 |
| 7 | 2611.89 | 2140.13 | 2267.59 |
| 8 | 928.03 | 822.41 | 1078.88 |
| 9 | 1411.17 | 2168.10 | 2426.48 |
| 10 | 3054.66 | 5658.69 | 6508.51 |
| 11 | 3945.07 | 6000.74 | 5126.16 |
| 12 | 2334.99 | 2841.92 | 3414.29 |
| 13 | 2145.84 | 2646.90 | 2641.59 |
| 14 | 2969.52 | 3503.08 | 3644.61 |
| 15 | 3459.53 | 5154.82 | 5550.52 |
| 16 | 2259.55 | 3151.10 | 2501.94 |
| 17 | 1931.06 | 2113.35 | 2643.88 |
| 18 | 3967.56 | 4097.72 | 4049.83 |

## Table 9: Hausdorff’s Distance (mm) values of Set B and Set C

| **Model** | **Set B** | **Set C** |
| --- | --- | --- |
| 1 | 0.132807 | 0.317946 |
| 2 | 0.349355 | 0.417358 |
| 3 | 0.638440 | 0.735324 |
| 4 | 1.128566 | 0.956966 |
| 5 | 0.010615 | 0.295171 |
| 6 | 0.352714 | 0.354508 |
| 7 | 0.346302 | 0.210964 |
| 8 | 0.054787 | 0.463034 |
| 9 | 0.771476 | 0.990601 |
| 10 | 1.412291 | 1.688051 |
| 11 | 1.060736 | 0.710374 |
| 12 | 0.448918 | 0.781318 |
| 13 | 0.484959 | 0.692067 |
| 14 | 0.419987 | 0.488710 |
| 15 | 1.053463 | 1.186459 |
| 16 | 0.732015 | 0.516511 |
| 17 | 0.326564 | 0.733361 |
| 18 | 0.133424 | 0.221406 |

## HD & overlapping area discordance data for phase D

### Set B


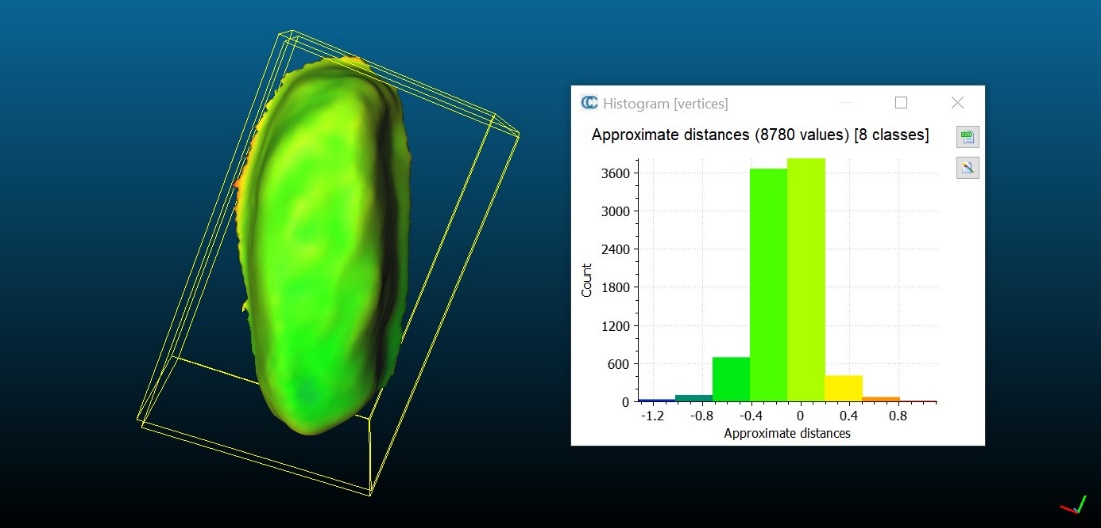


Bulb 1


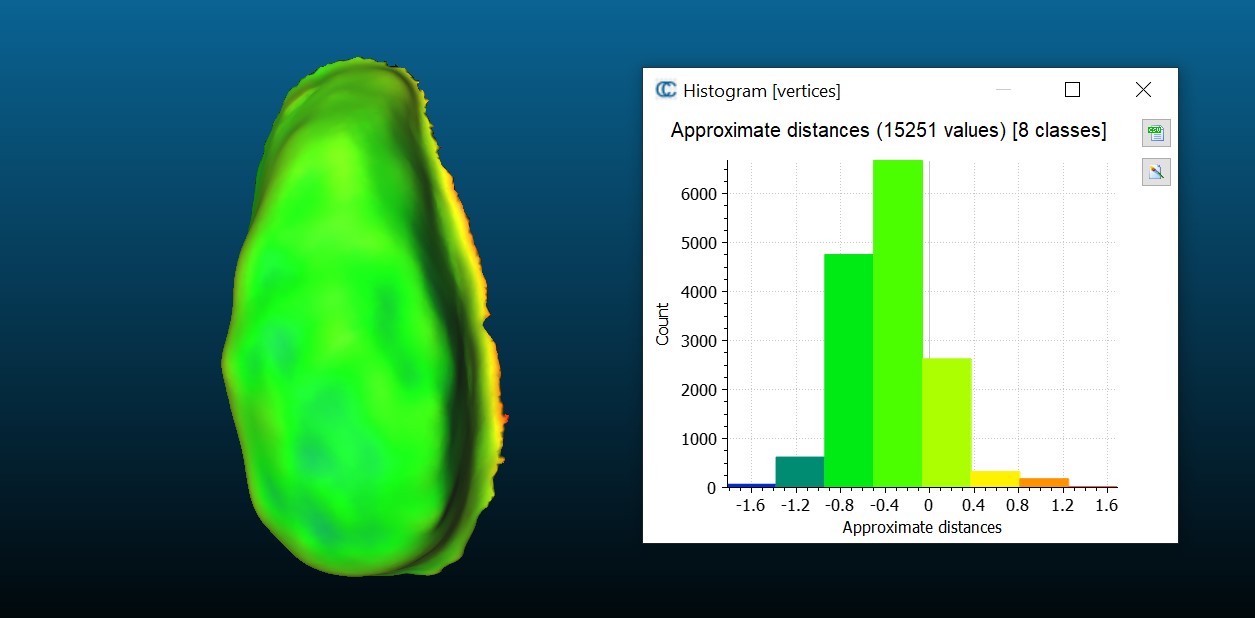


Bulb 2


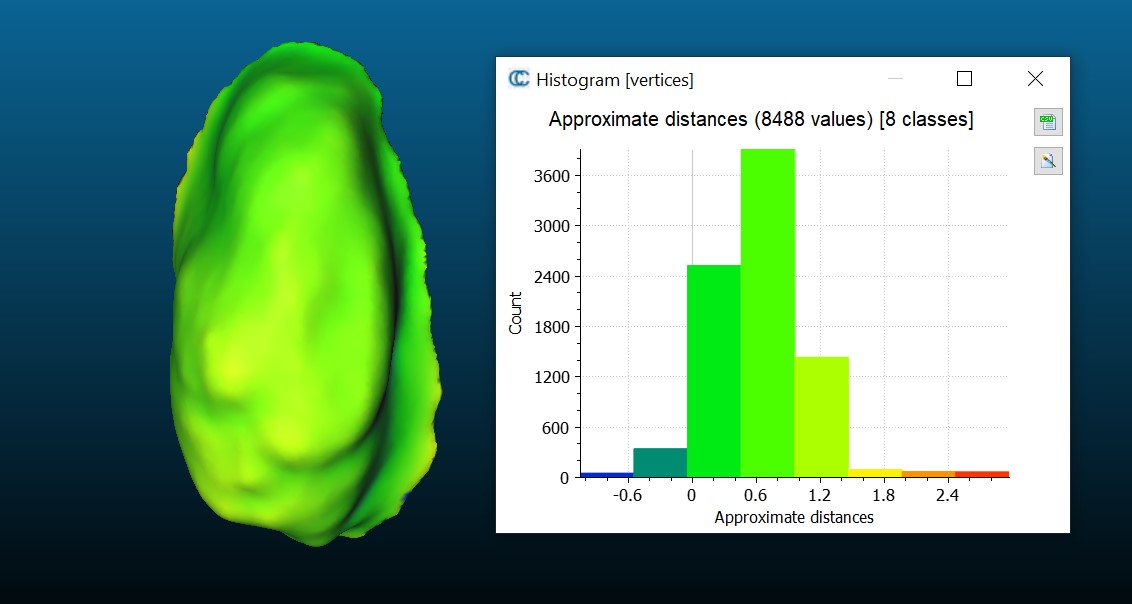


Bulb 3


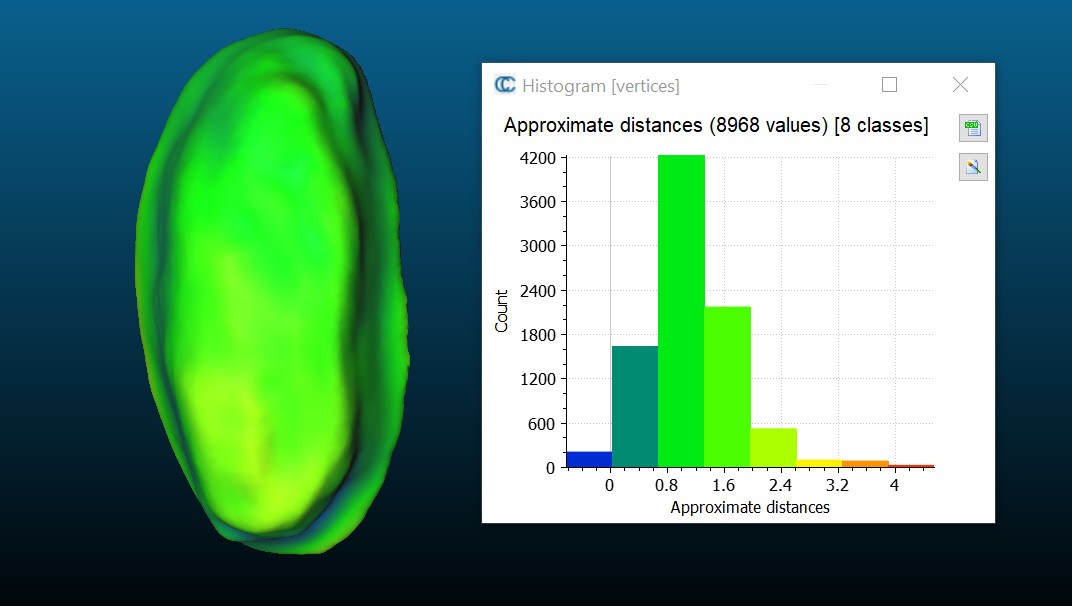


Bulb 4


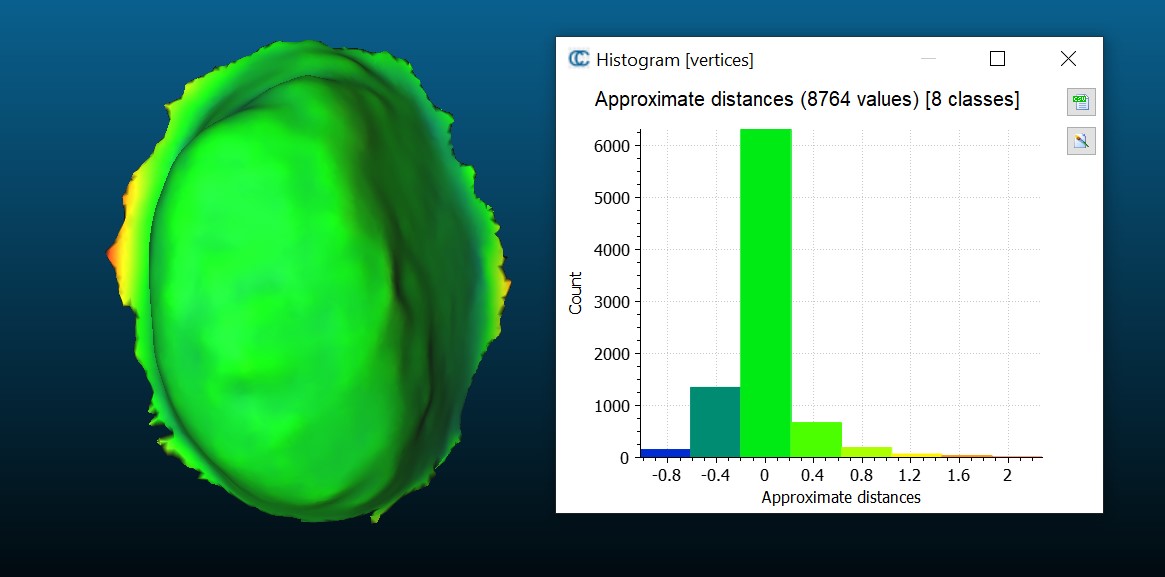


Bulb 5


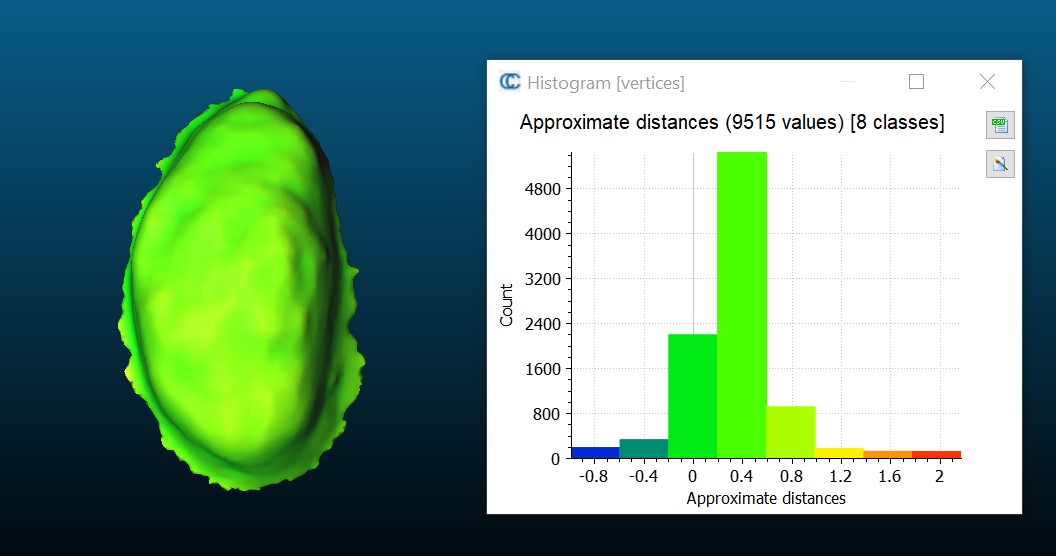


Bulb 6


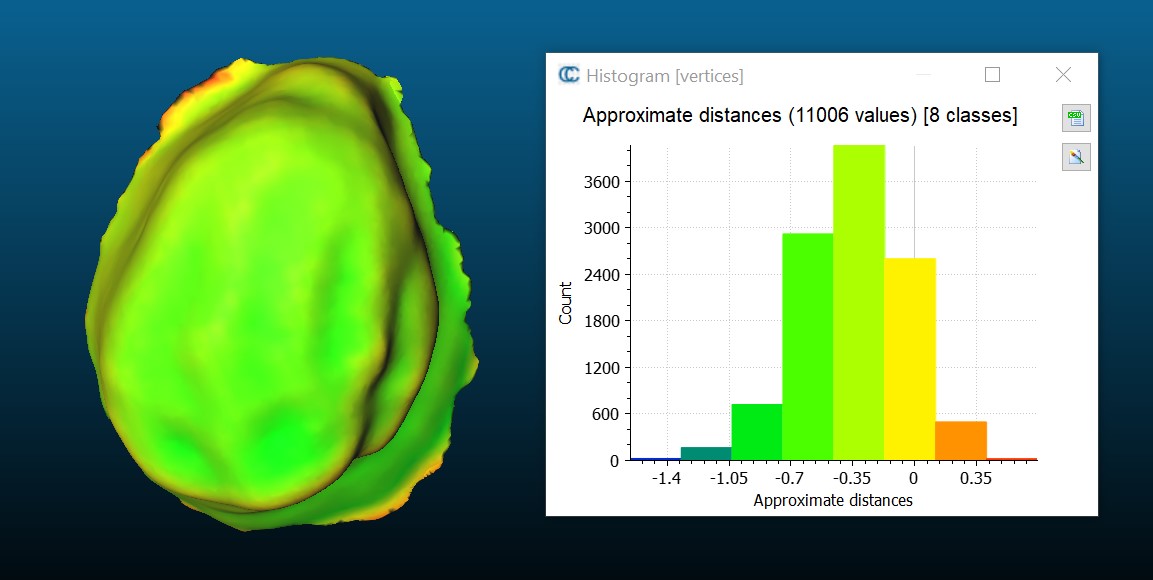


Bulb 7


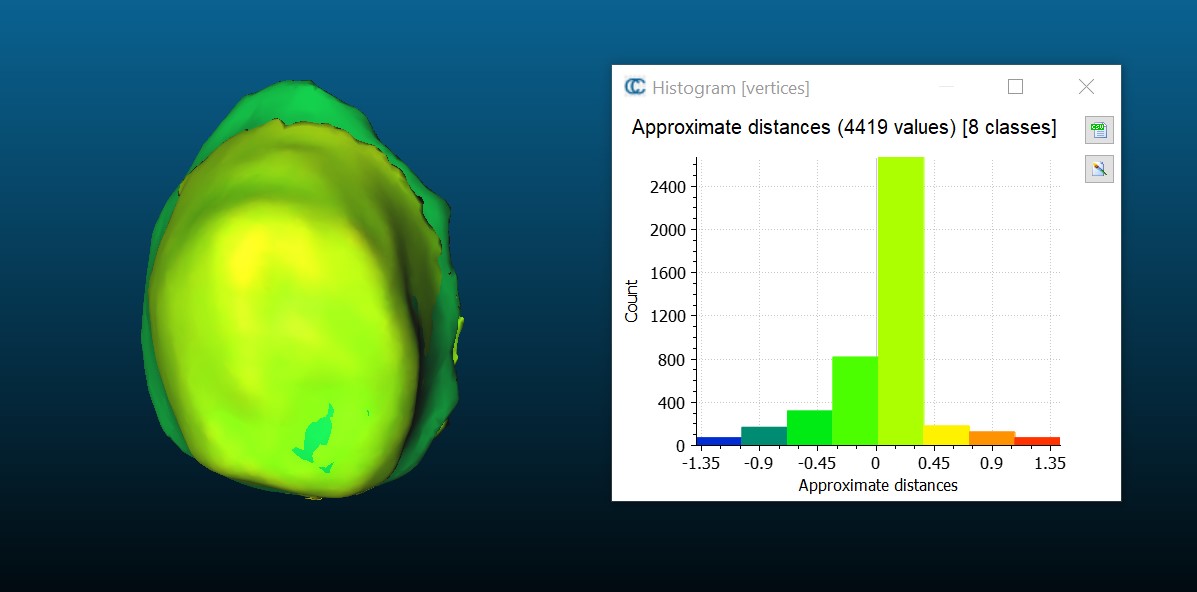


Bulb 8


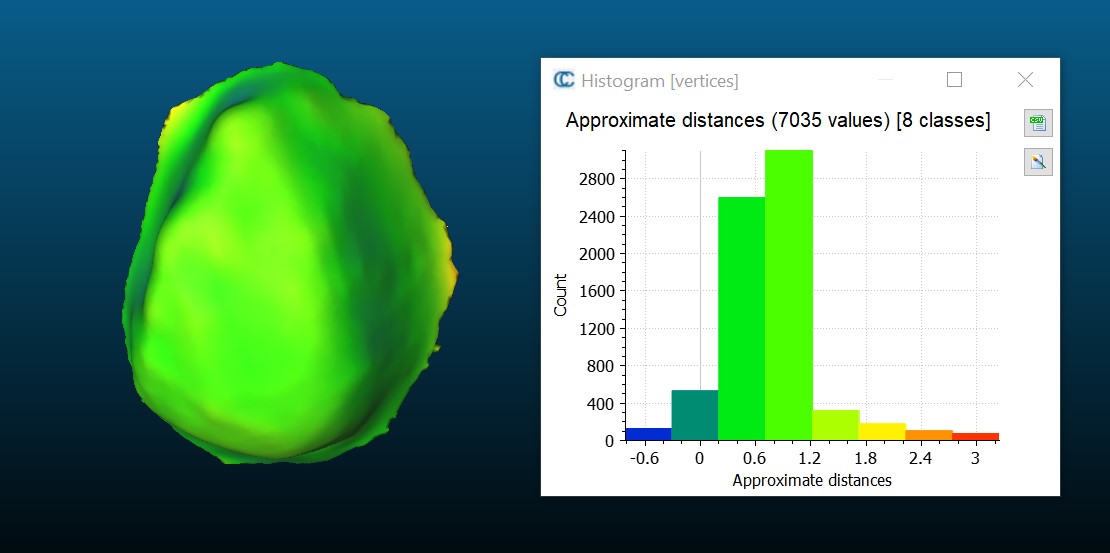


Bulb 9


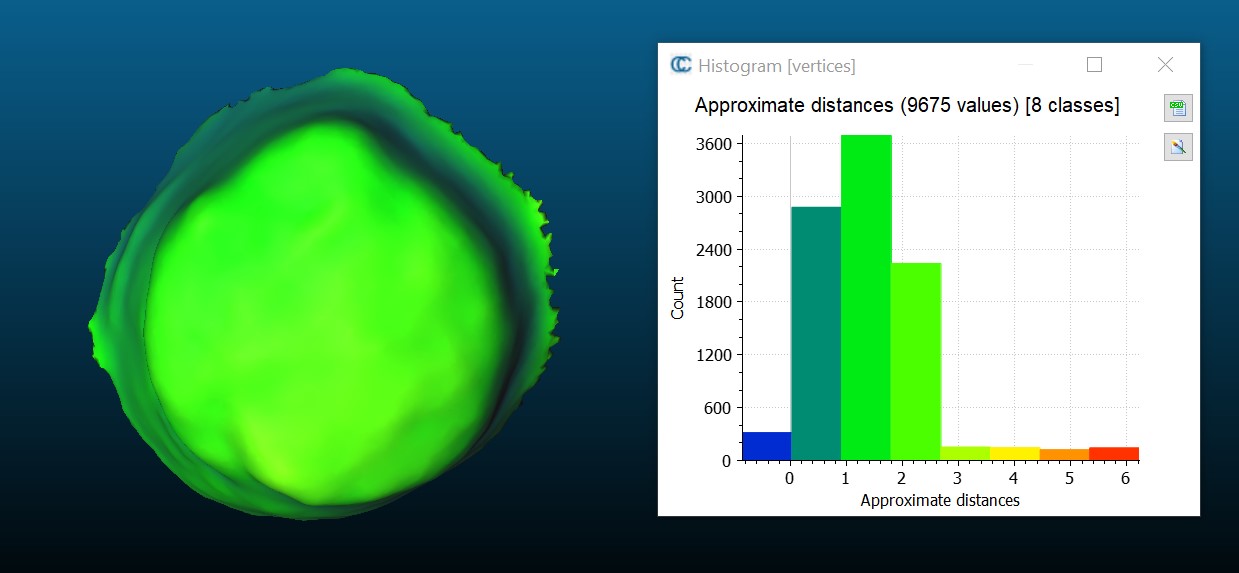


Bulb 10


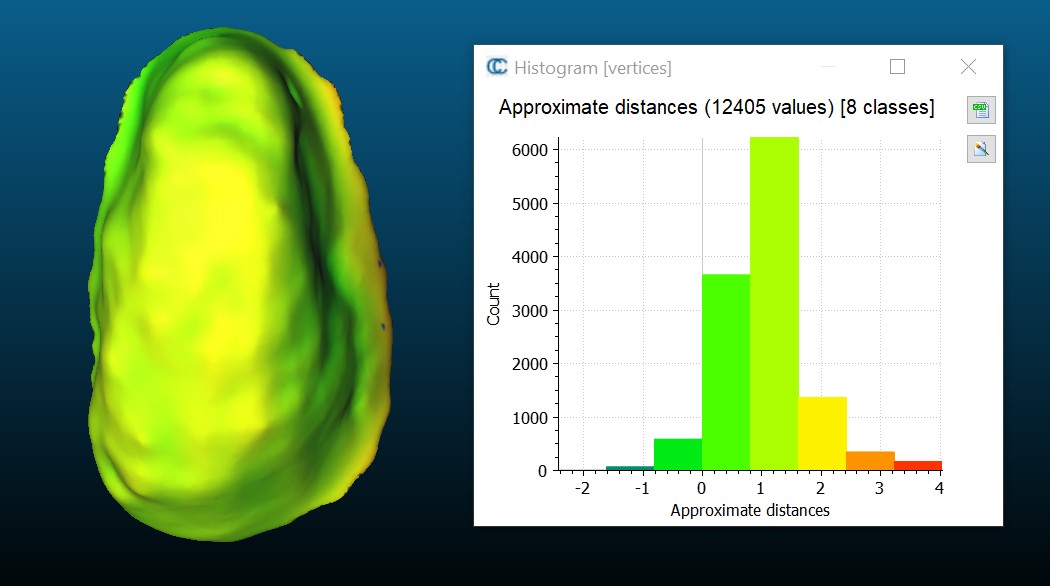


Bulb 11


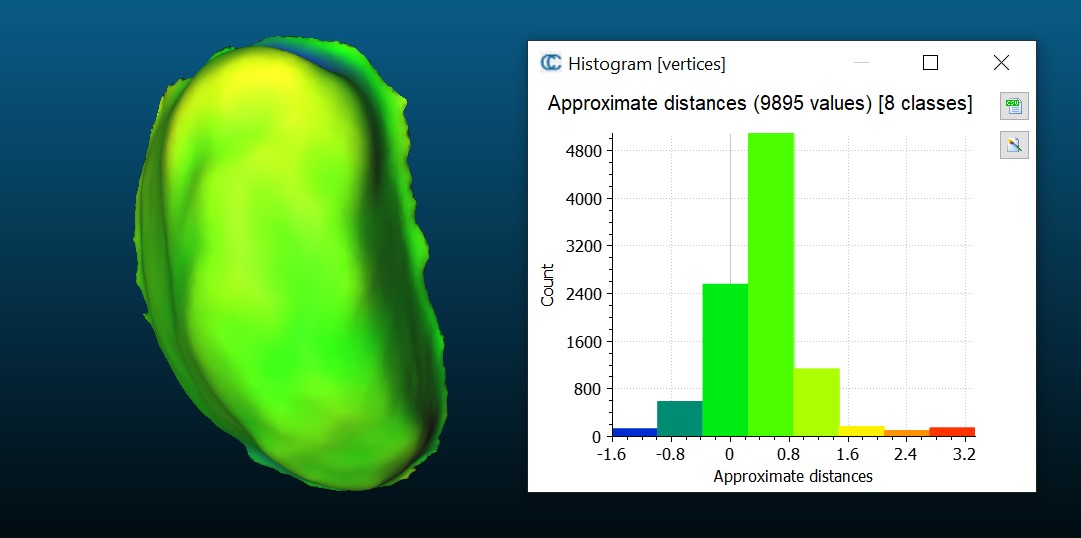


Bulb 12


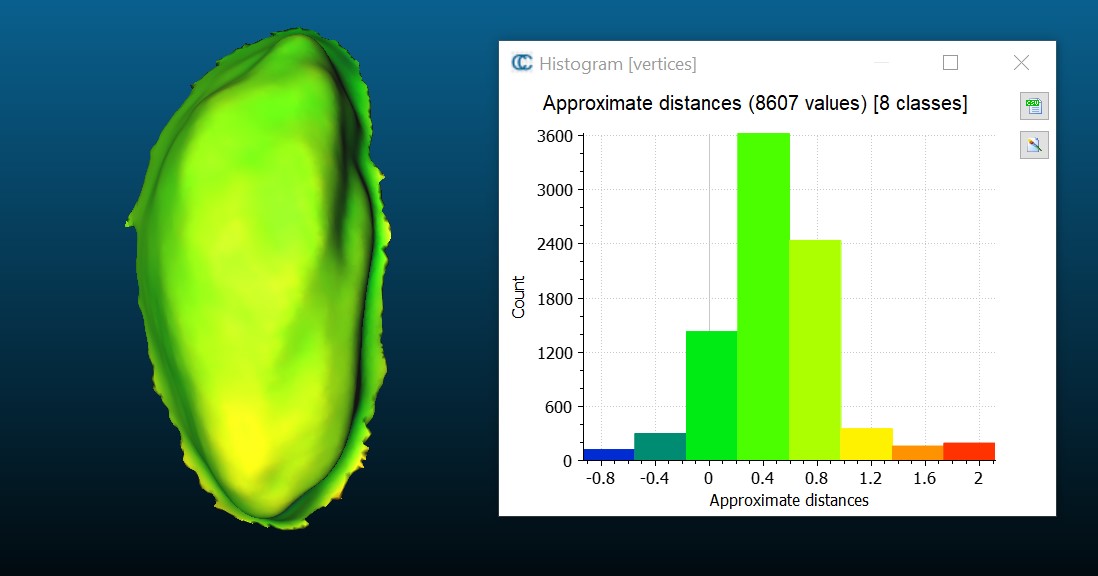


Bulb 13


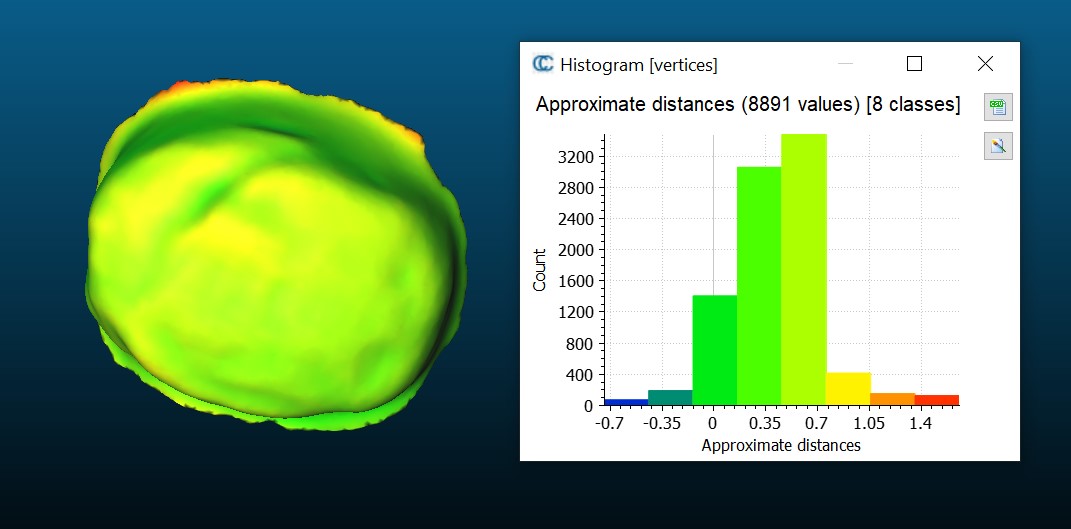


Bulb 14


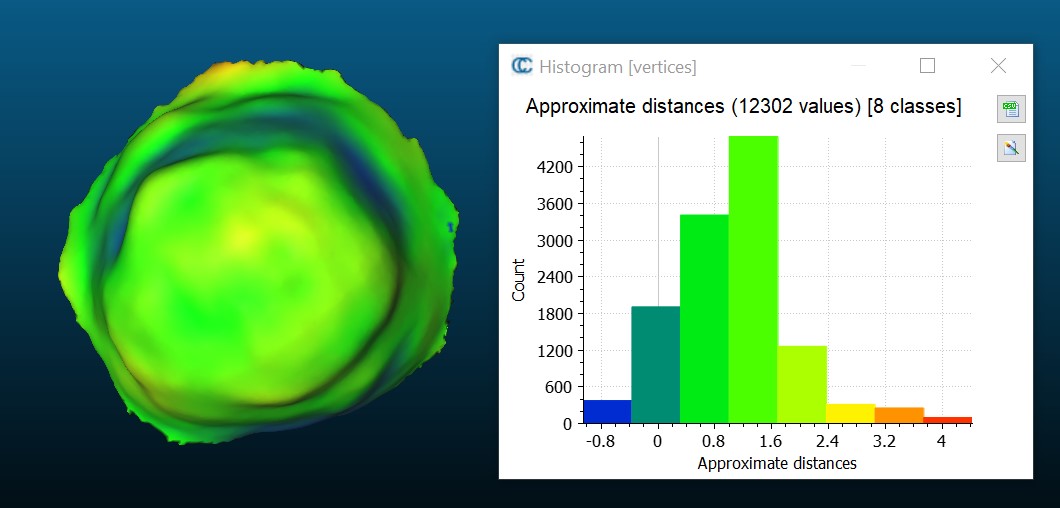


Bulb 15


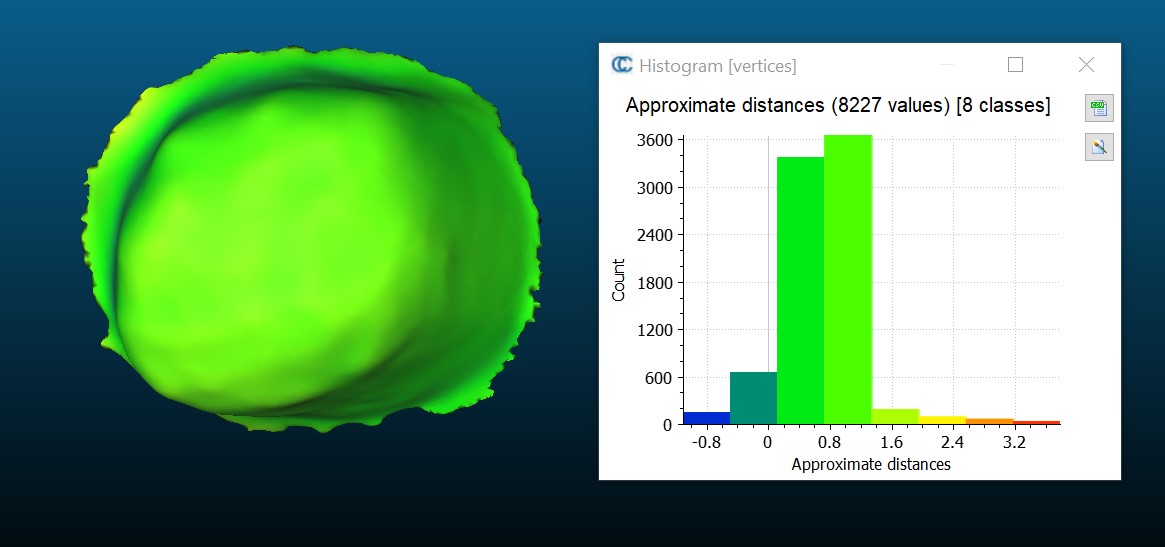


Bulb 16


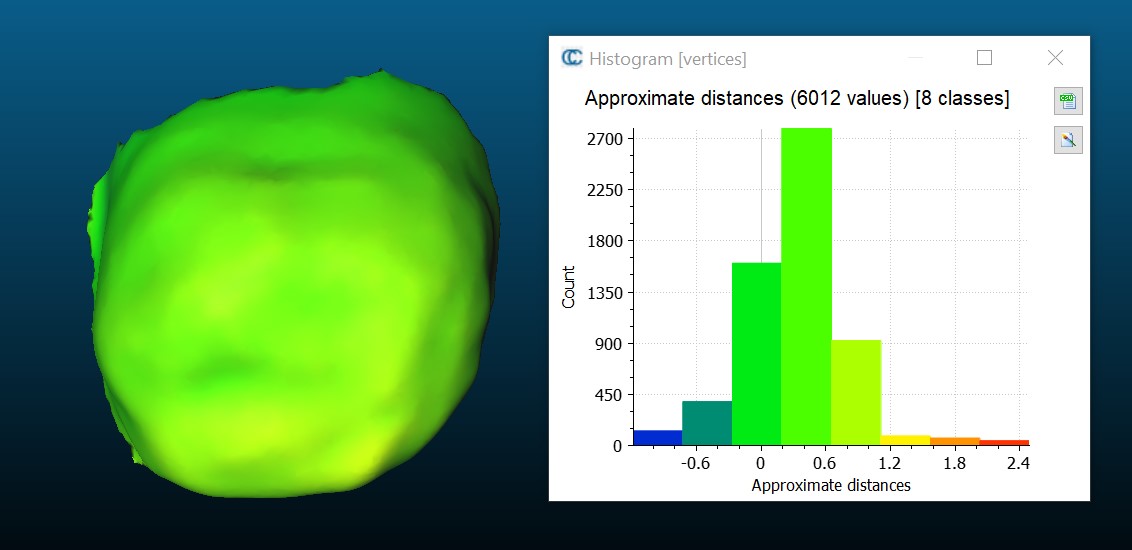


Bulb 17


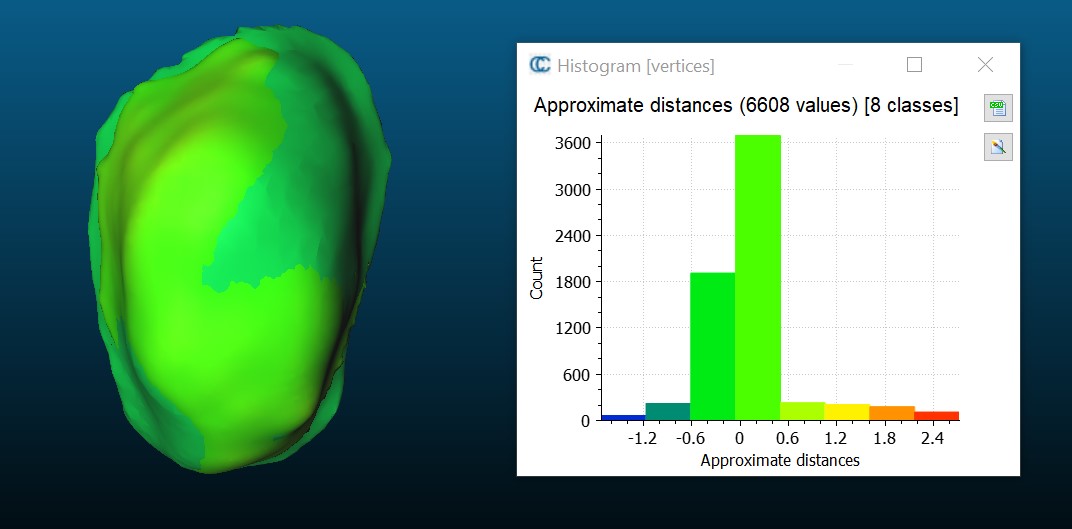


Bulb 18

### Set C


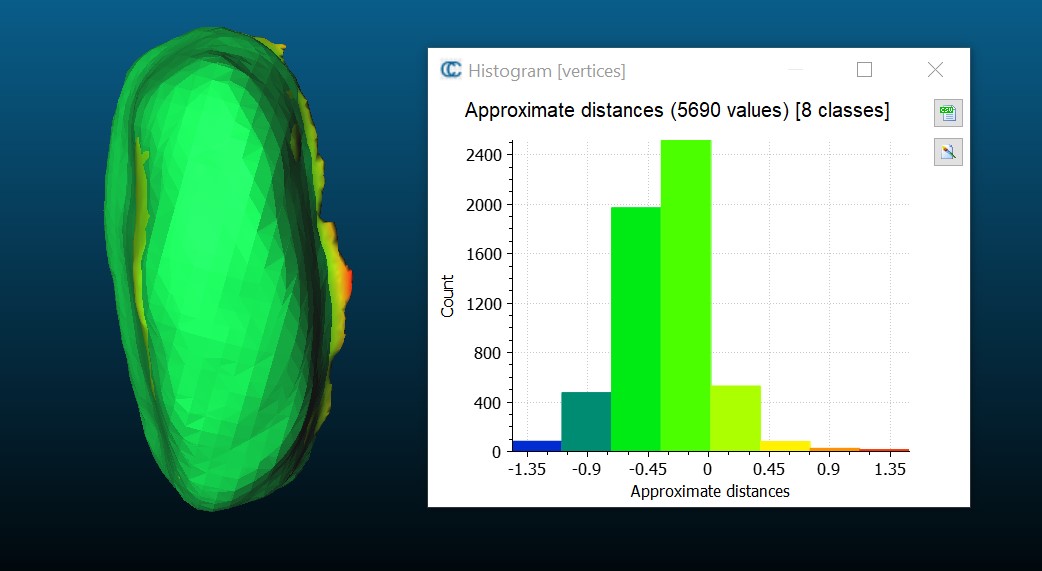


Bulb 1


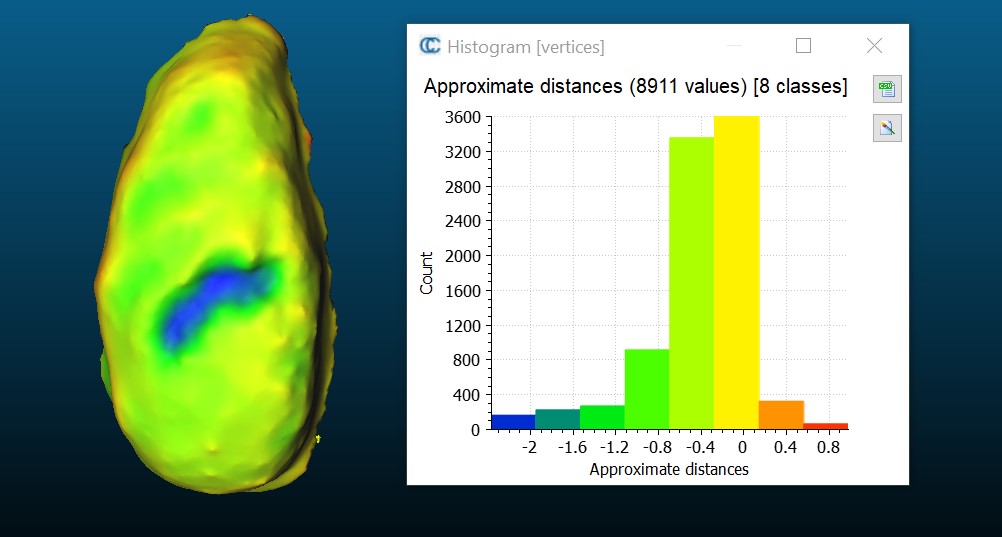


Bulb 2


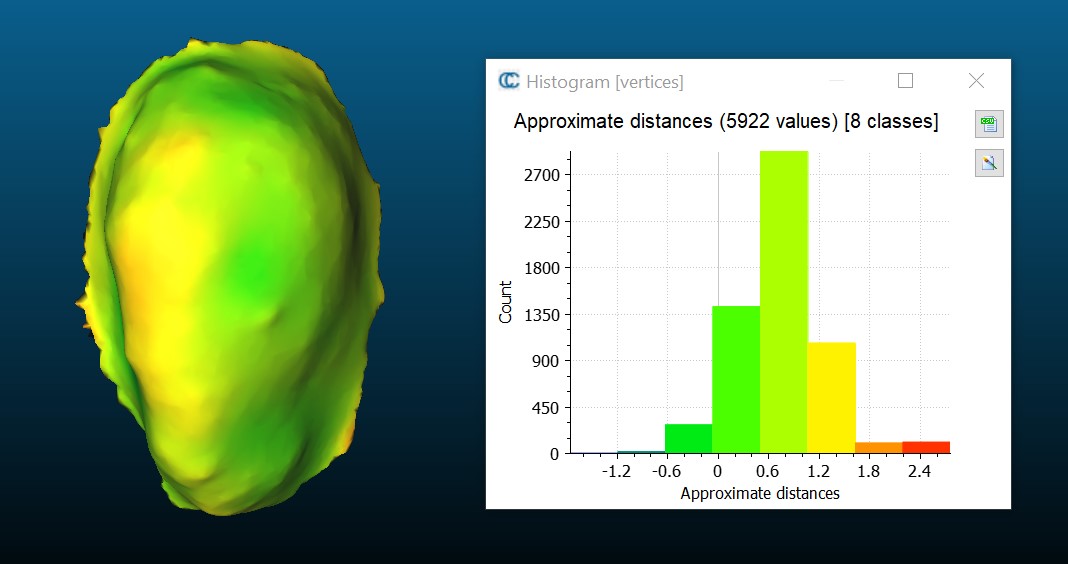


Bulb 3


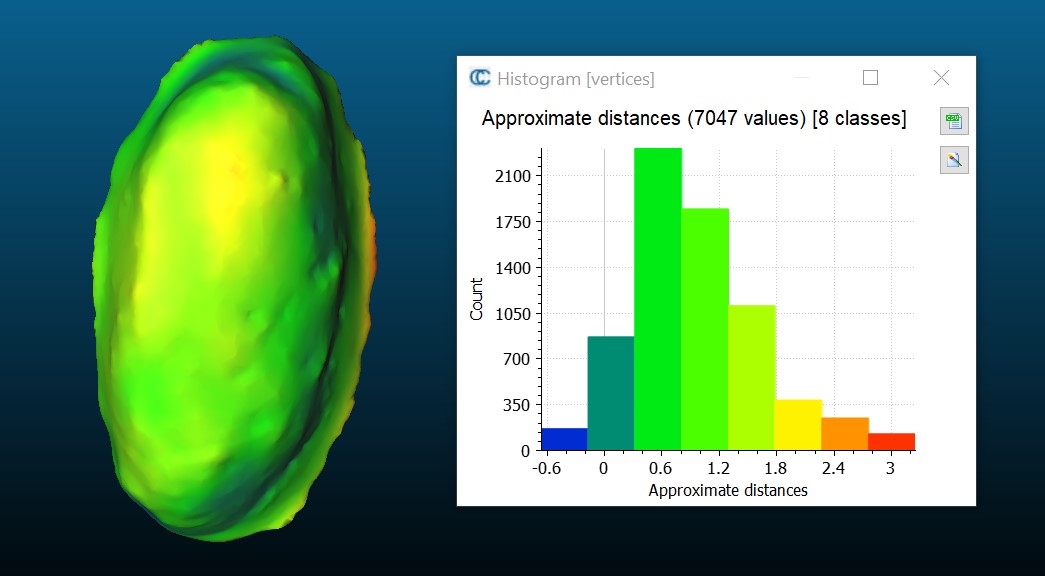


Bulb 4


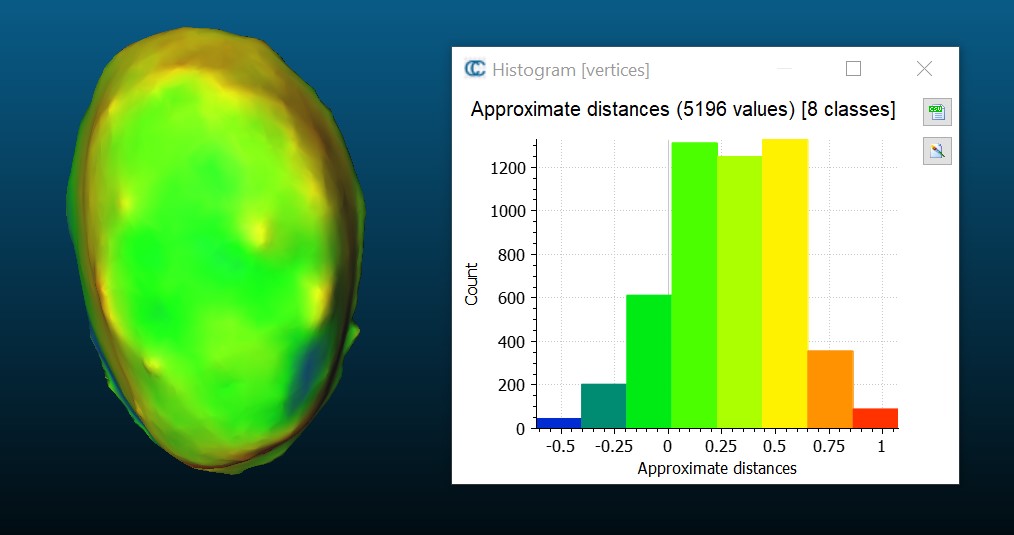


Bulb 5


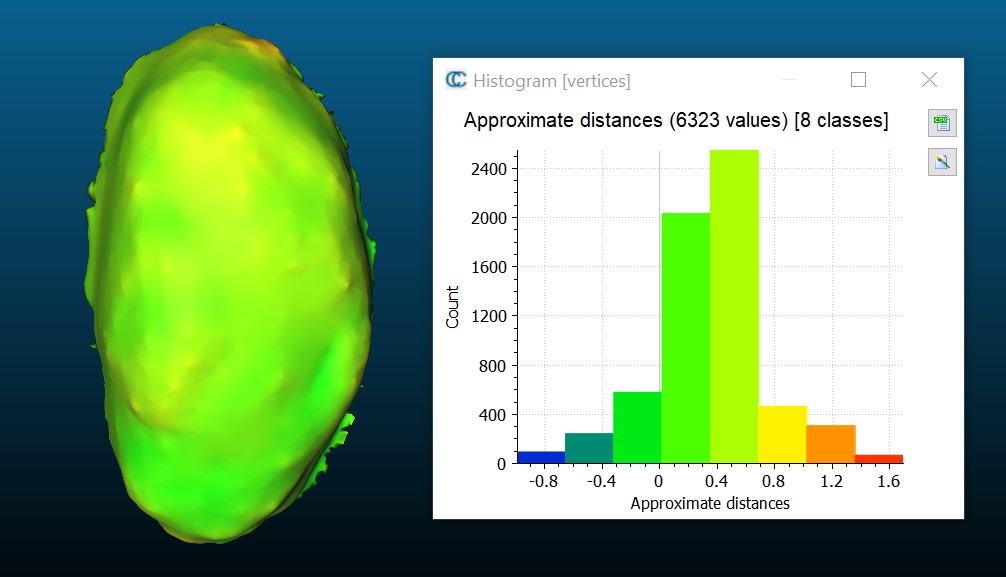


Bulb 6


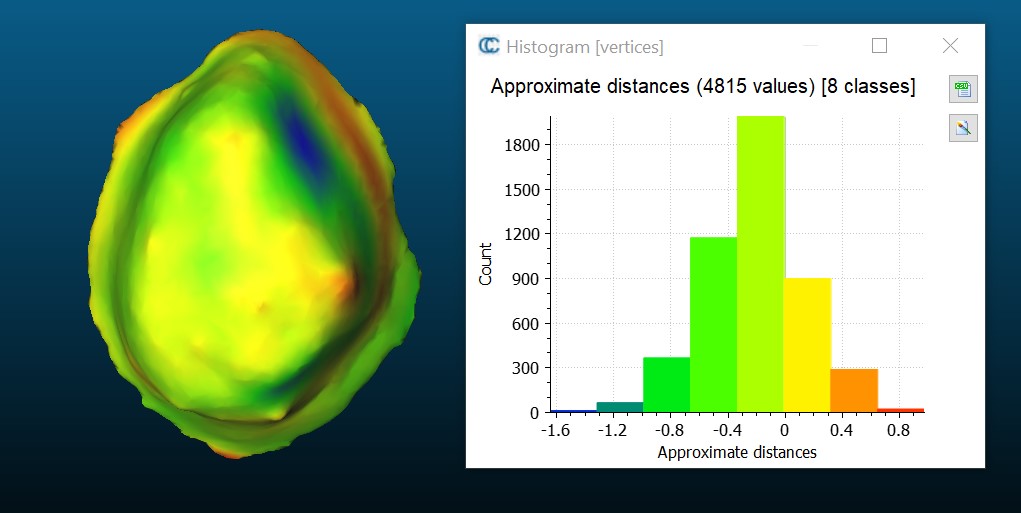


Bulb 7


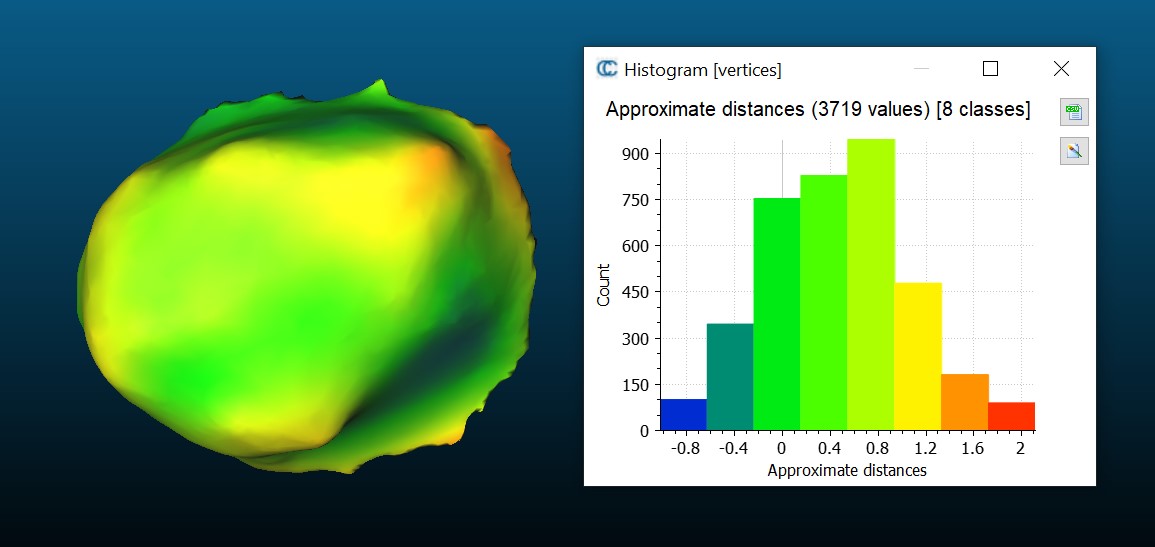


Bulb 8


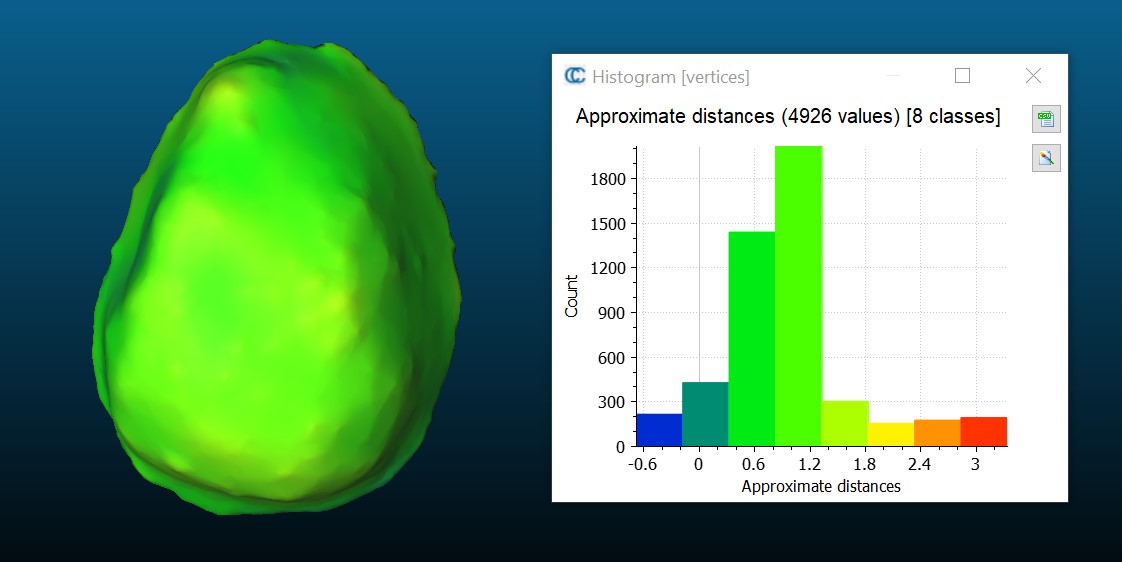


Bulb 9


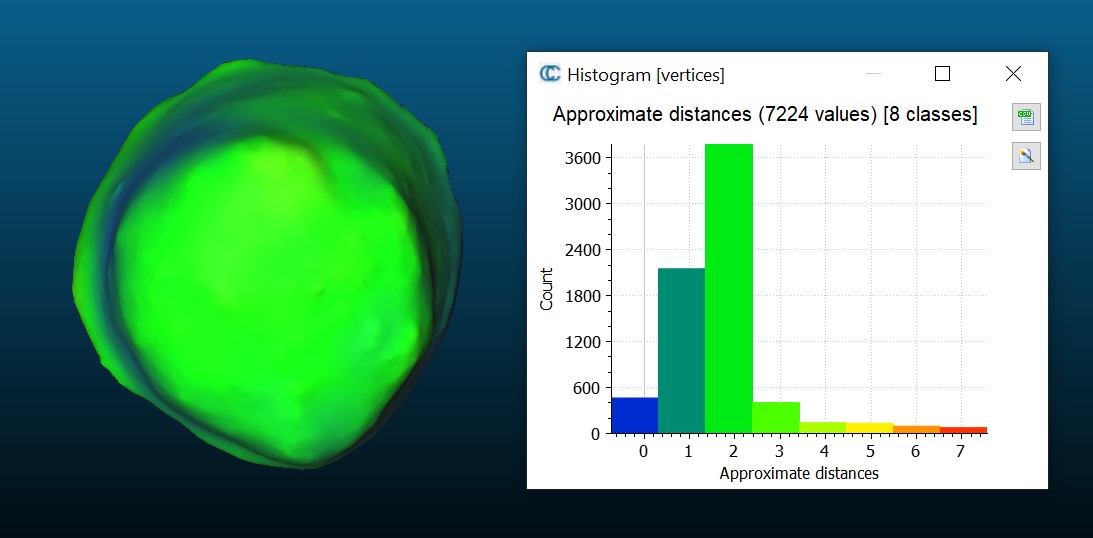


Bulb 10


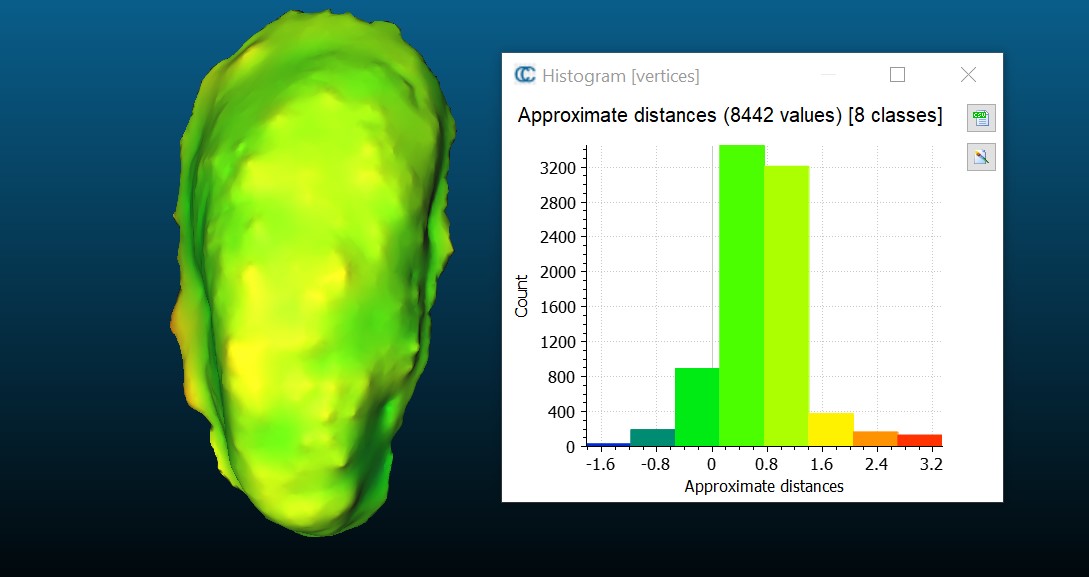


Bulb 11


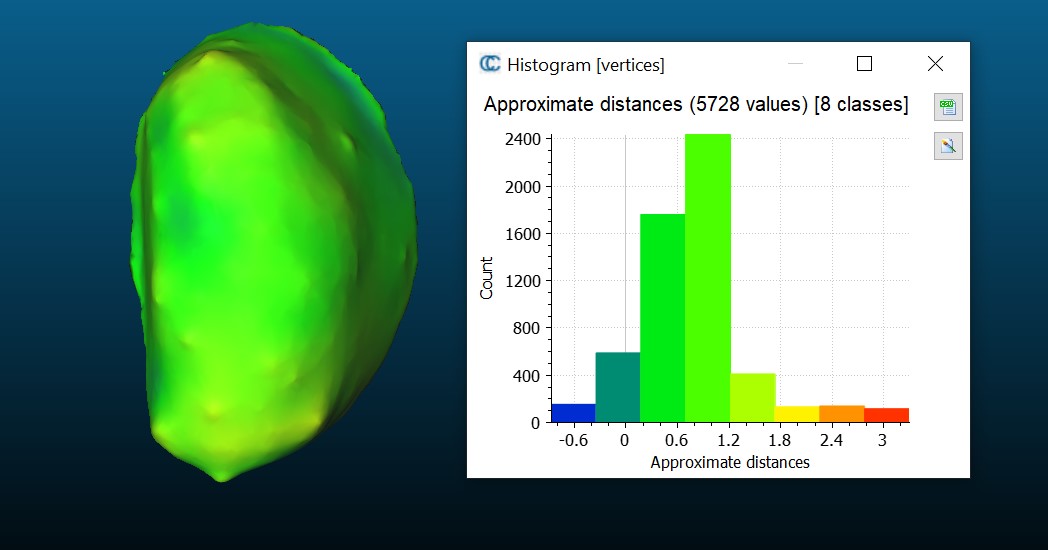


Bulb 12


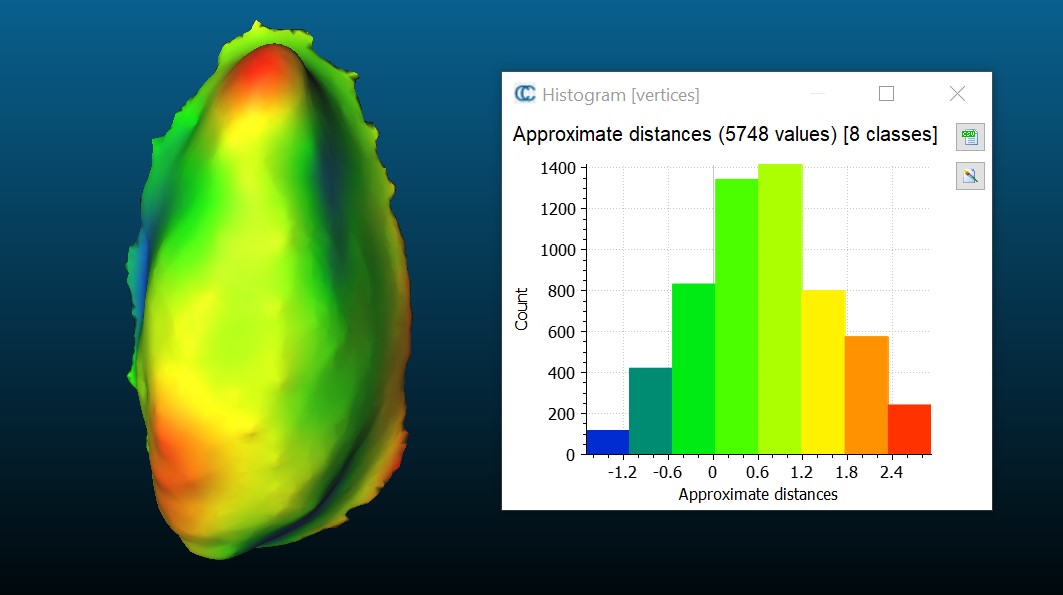


Bulb 13


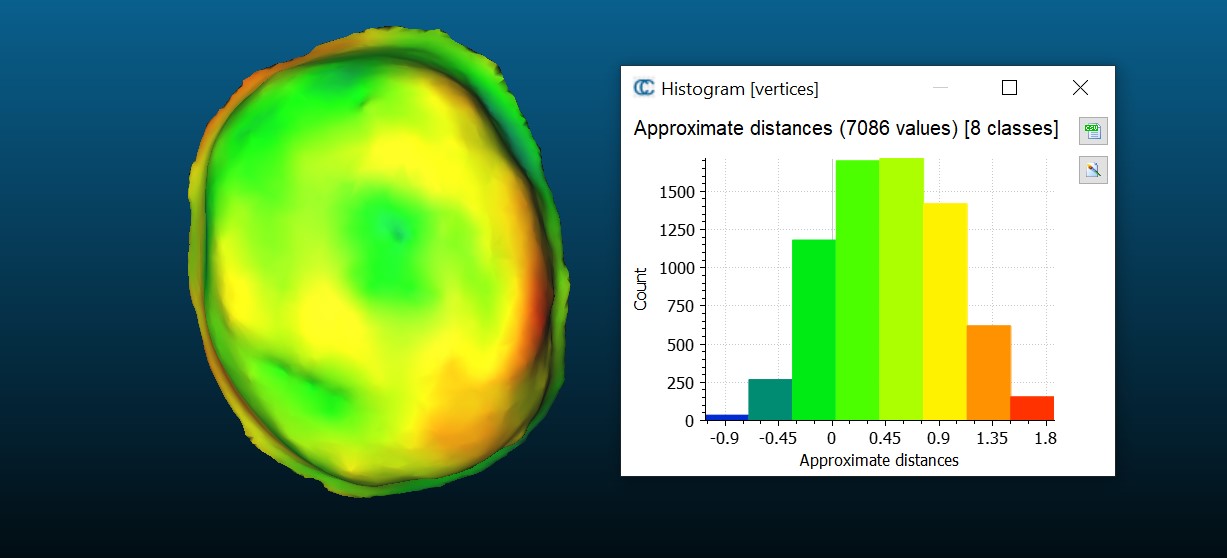


Bulb 14


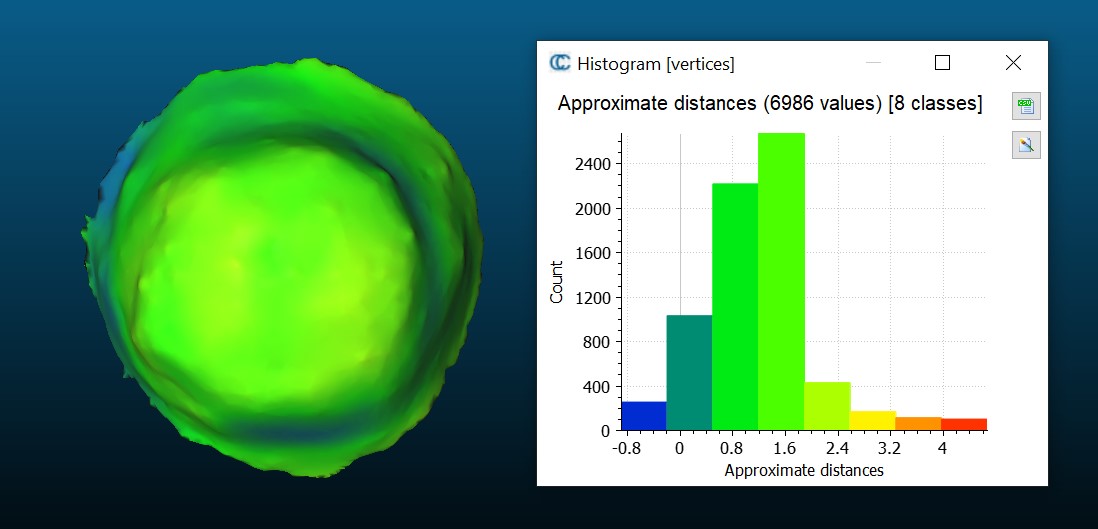


Bulb 15


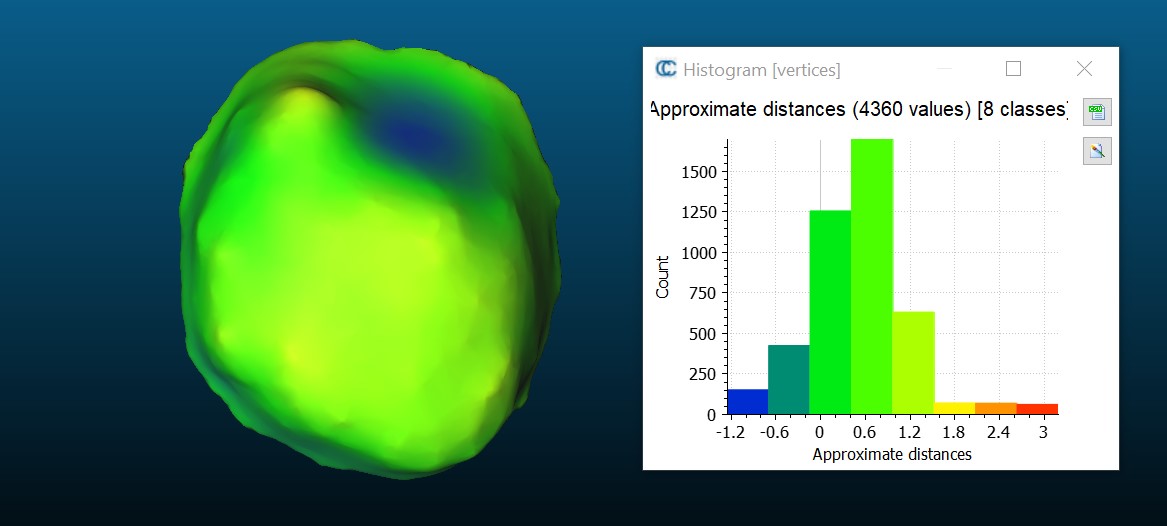


Bulb 16


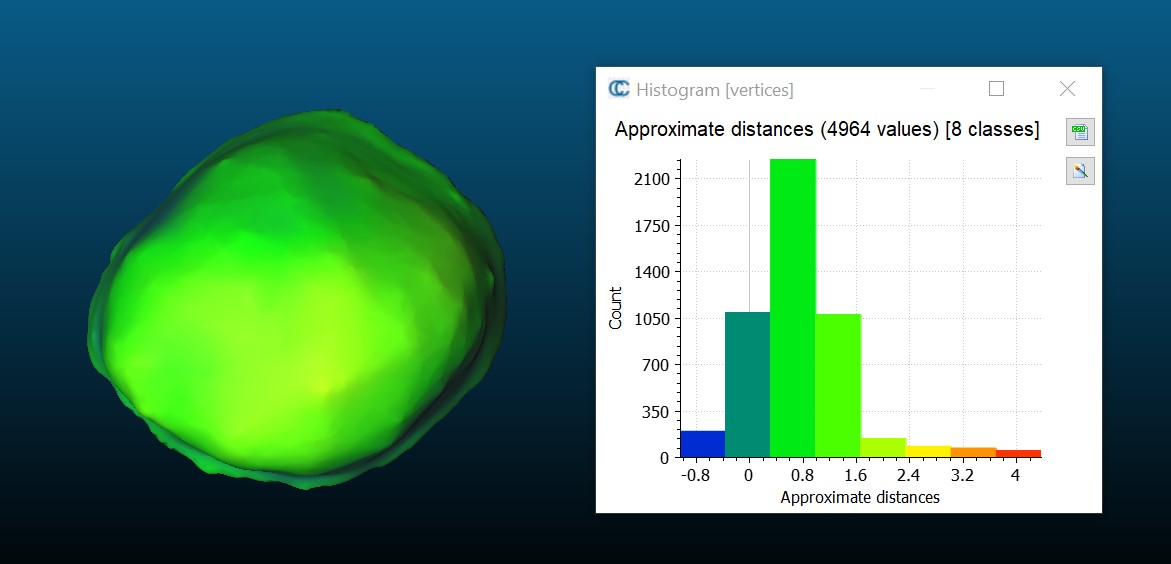


Bulb 17


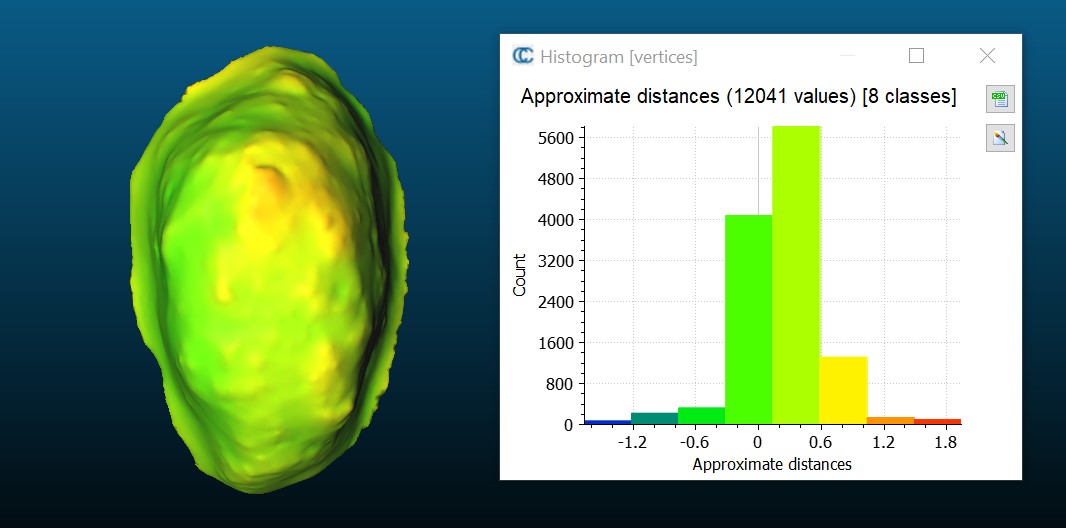


Bulb 18

## Table 10: Dice similarity co-efficient values for Set B and Set C

| **Model** | **Set B** | **Set C** |
| --- | --- | --- |
| 1 | 0.940564 | 0.895220 |
| 2 | 0.919263 | 0.909599 |
| 3 | 0.798616 | 0.783429 |
| 4 | 0.649046 | 0.700410 |
| 5 | 0.958510 | 0.927542 |
| 6 | 0.852215 | 0.857406 |
| 7 | 0.896962 | 0.908814 |
| 8 | 0.842428 | 0.788127 |
| 9 | 0.748963 | 0.710400 |
| 10 | 0.665085 | 0.617088 |
| 11 | 0.751299 | 0.795257 |
| 12 | 0.835595 | 0.785555 |
| 13 | 0.820349 | 0.754480 |
| 14 | 0.868628 | 0.850392 |
| 15 | 0.748630 | 0.736171 |
| 16 | 0.775267 | 0.808016 |
| 17 | 0.828130 | 0.755888 |
| 18 | 0.888142 | 0.893423 |

***DSC calculation breakdown:***

$$\frac{2*(A\cap B)}{A+B}$$

| **SET B** | **SET C** |
| --- | --- |
| Model 1: $\frac{2* 2668.28}{2975.47+2698.32}$ = 0.940564 | Model 1: $\frac{2* 2421.87}{2975.47+2435.20}$ = 0.895220 |
| Model 2: $\frac{2* 5866.19}{6868.75+5894.06}$ = 0.919263 | Model 2: $\frac{2* 5748.02}{6868.75+5770.23}$ = 0.909599 |
| Model 3:$\frac{2* 1769.16}{1847.84+2582.72}$ = 0.798616 | Model 3:$\frac{2* 1766.10}{1847.84+2660.80}$ = 0.783429 |
| Model 4:$\frac{2* 1501.27}{1529.83+3096.25}$ = 0.649046 | Model 4:$\frac{2* 1485.96}{1529.83+2713.29}$ = 0.700410 |
| Model 5:$\frac{2* 2501.43}{2639.87+2579.56}$ = 0.958510 | Model 5:$\frac{2* 2607.89}{2639.87+2983.36}$ = 0.927542 |
| Model 6:$\frac{2* 2275.93}{2519.89+2821.32}$ = 0.852215 | Model 6:$\frac{2* 2265.24}{2519.89+2764.05}$ = 0.857406 |
| Model 7:$\frac{2* 2131.19}{2611.89+2140.13}$ = 0.896962 | Model 7:$\frac{2* 2217.27}{2611.89+2267.59}$ = 0.908814 |
| Model 8:$\frac{2* 737.31}{928.03+822.41}$ = 0.842428 | Model 8:$\frac{2* 790.85}{928.03+1078.88}$ = 0.788127 |
| Model 9:$\frac{2* 1340.37}{1411.17+2168.10}$ = 0.748963 | Model 9:$\frac{2* 1363.00}{1411.17+2426.10}$ = 0.710400 |
| Model 10:$\frac{2* 2897.56}{3054.66+5658.69}$ = 0.665085 | Model 10:$\frac{2* 2950.66}{3054.66+6508.51}$ = 0.617088 |
| Model 11:$\frac{2* 3739.14}{3945.07+6000.74}$ = 0.751299 | Model 11:$\frac{2* 3606.98}{3945.07+5126.16}$ = 0.795257 |
| Model 12:$\frac{2* 2162.9}{2334.99+2841.92}$ = 0.835595 | Model 12:$\frac{2* 2258.19}{2334.99+3414.29}$ = 0.785555 |
| Model 13:$\frac{2* 1965.86}{2145.84+2646.90}$ = 0.820349 | Model 13:$\frac{2* 1806.01}{2145.84+2641.59}$ = 0.754480 |
| Model 14:$\frac{2* 2811.14}{2969.52+3503.08}$ = 0.868628 | Model 14:$\frac{2* 2812.30}{2969.52+3644.61}$ = 0.850392 |
| Model 15:$\frac{2* 3224.48}{3459.53+5154.82}$ = 0.748630 | Model 15:$\frac{2* 3316.47}{3459.53+5550.52}$ = 0.736171 |
| Model 16:$\frac{2* 2097.35}{2259.55+3151.1}$ = 0.775267 | Model 16:$\frac{2* 1923.68}{2259.55+2501.94}$ = 0.808016 |
| Model 17:$\frac{2* 1674.65}{1931.06+2113.35}$ = 0.828130 | Model 17:$\frac{2* 1729.07}{1931.06+2643.88}$ = 0.755888 |
| Model 18:$\frac{2* 3581.56}{3967.56+4097.72}$ = 0.888142 | Model 18:$\frac{2* 3581.46}{3967.56+4049.83}$ = 0.893423 |
